# Scoping review of patients’ attitudes about their role and behaviours to ensure safe care at the direct care level

**DOI:** 10.1111/hex.13117

**Published:** 2020-08-05

**Authors:** Lenora Duhn, Christina Godfrey, Jennifer Medves

**Affiliations:** ^1^ School of Nursing Queen’s University Kingston ON Canada

**Keywords:** direct care level, patient attitudes, patient behaviours, patient engagement, patient safety, scoping review

## Abstract

**Background:**

To improve harm prevention, patient engagement in safety at the direct care level is advocated. For patient safety to most effectively include patients, it is critical to reflect on existing evidence, to better position future research with implications for education and practice.

**Methods:**

As part of a multi‐phase study, which included a qualitative descriptive study (Duhn & Medves, 2018), a scoping review about patient engagement in safety was conducted. The objective was to review papers about patients’ attitudes and behaviours concerning their involvement in ensuring their safe care. The databases searched included MEDLINE, CINAHL and EMBASE (year ending 2019).

**Results:**

This review included 35 papers about “Patient Attitudes” and 125 papers about “Patient Behaviours”—indicative of growing global interest in this field. Several patterns emerged from the review, including that most investigators have focused on a particular dimension of harm prevention, such as asking about provider handwashing, and there is less known about patients’ opinions about their role in safety generally and how to actualize it in a way that is right for them. While patients may indicate favourable attitudes toward safety involvement generally, intention to act or actual behaviours may be quite different.

**Conclusion:**

This review, given its multi‐focus across the continuum of care, is the first of its kind based on existing literature. It provides an important international “mapping” of the initiatives that are underway to engage patients in different elements of safety and their viewpoints, and identifies the gaps that remain.

## 1 INTRODUCTION

The need to improve patient safety continues to be paramount. Patient safety is recognized as a serious global public health issue, with approximately one in 20 patients harmed while receiving medical care (primary, secondary and tertiary care settings).^1^ When considering patient safety, it is important to understand the perspectives of patients and their families, and to recognize the need for more effective involvement of patients and families across the continuum of care.^2^ As recipients of health‐care services, patients and their families are a valuable resource and contribute to our understanding of safety issues. To advance our knowledge about patient and family involvement in promoting health‐care safety, as distinct from their health generally, a multi‐phase study was conducted that included a qualitative study of patients’ perspectives about their knowledge, comfort level and behaviours in helping their safety while receiving health care.^3^ This scoping review was phase 2, and the aim was to describe the literature about patients’ and families’ attitudes toward their role in health‐care safety, as well as their reported behaviours in support of their safe care. A general multidimensional framework about patient and family engagement in health and health care underpinned this study.^4^

## 2 BACKGROUND

In preparation for the multi‐phase study, a preliminary examination of the literature, which included findings from previous unpublished work,^5^ provided an initial understanding of the evidence about patient perspectives regarding health‐care safety, and views on their involvement in safety processes. The findings from this preparatory work, which helped determine the need for a scoping review, included the following concepts.

### 2.1 Communication

Patients and/or their families specifically alluded to communication as a vital part of patient safety.^6^, ^7^, ^8^, ^9^, ^10^, ^11^, ^12^, ^13^, ^14^, ^15^, ^16^, ^17^, ^18^, ^19^, ^20^, ^21^, ^22^ Participant views on this theme were common across primary care, ambulatory, hospital and home settings. In a Canadian telephone survey of 1,500 adults, respondents perceived that communication was associated with preventable medical errors.^20^ In the primary care studies, safety problems were linked to miscommunication and/or a lack in communication of test results.^8^, ^9^, ^12^

### 2.2 Trust

The concept of trust in the health‐care system and providers was also a common theme related to patient safety as perceived by patients and families.^6^, ^9^, ^11^, ^13^, ^23^, ^24^, ^25^ The overwhelming need of “safety” in ill hospitalized patients was tempered by the knowledge of being able to trust staff.^11^ A group of 30 ambulatory patients receiving chemotherapy treatment recognized their own limitations in safety prevention, including deficits in knowledge, and therefore felt they had to trust the providers.^19^ In another example, some parents, who believed their children had experienced a medical error, expressed regret at having trusted a health‐care professional.^13^

### 2.3 Being trusted, seen and valued

Patients and their families view being trusted for their knowledge, respected and valued for their contribution as integral to safe patient care across hospital, outpatient and home settings.^8^, ^13^, ^16^, ^17^, ^26^, ^27^, ^28^ Reports of patients’ experiences in primary care echoed the related theme of difficulties in interpersonal relationships with providers, particularly noting disrespect and insensitivity.^12^ A study of 20 individuals with Parkinson's disease (or someone who spoke on their behalf—partner, wife, husband, daughter) and their experiences of medication errors most poignantly captures the lack of respect of patients’ knowledge related to their medication management and the de‐valuing of their insights—the cost of which can jeopardize a patient's safety.^27^

### 2.4 Patients as partners

Patients’ beliefs about participating in patient safety in different ways have also been investigated.^19^, ^24^, ^29^, ^30^, ^31^, ^32^, ^33^, ^34^ Positive attitudes about safety engagement have been identified, with qualifiers such as the type of action required by the patient, practitioners receiving this level of involvement by patients and their families, as well as the setting.^3^ Tactics that patients (or family members of patients) use to protect themselves when interacting with the health‐care system have been reported, including bringing family or friends to appointments and questioning unfamiliar medication.^8^, ^9^, ^18^, ^25^, ^35^, ^36^, ^37^ Patient involvement in ameliorating harm has also been documented.^19^, ^21^, ^38^, ^39^, ^40^ Generally, the most common approach to engage patients in health‐care safety has taken the form of patient “tips” or strategies outlined in patient information or educational materials. There is evidence that some of these tips have been created with input from patients; however, this is not true of all recommendations, with a need for evidence‐based content and evaluation.^41^, ^42^

Given the imperative of considering all approaches to reduce harm, as well as the importance of being patient‐ and family‐centred, the preliminary work identified gaps and additional questions (including do patients intentionally think about their involvement in safety at the direct care level, and what does safety mean to them) necessitating and providing rationale to explore international evidence in a more fulsome, comprehensive way related to patient and family involvement in safety at the direct care level.

## 3 METHODS

The scoping review was conducted to understand the breadth and depth of literature about patients’ engagement in safe care. The intention of the review was to gather as much relevant literature about patients’ attitudes toward having a role in preventing harm, as well as any safety behaviours they independently engage in or at the invitation of researchers or providers.

This scoping review was conducted using the search term “patient safety”, but other iterations were also considered given patients/families may understand it in different ways. Additionally, the preparatory work was informative in identifying that researchers typically investigate components of safety (eg handwashing), and therefore, these elements were part of the search strategy. A search of the JBI (Joanna Briggs Institute) Evidence Synthesis Journal and Cochrane database indicated that no scoping review had been conducted on this topic.

### 3.1 Research questions

(a) What are patients’ and families’ attitudes and beliefs about their participation role in ensuring they receive safe care? (b) What are the behaviours indicative of harm prevention that patients and families engage in independently or at the direction of others [researchers; health‐care providers]?

### 3.2 Research design

Peters and colleagues indicate the appropriateness of a scoping review when “a body of literature…exhibits a large, complex or heterogeneous nature not amenable to a more precise systematic review”.^43^ ^(p. 141)^ A generic methodology was taken to scope the literature on this topic, and this was overseen by the first author (LD) as part of her doctoral thesis, who was responsible for the management of data collection and analysis. Decisions were made primarily by the first author, and the thesis committee provided assistance if there were disagreements. Scoping reviews are unique in that they are typically about broader topics; often include many different study designs; and do not usually involve an assessment of the quality of studies that are included in the review. ^44^

### 3.3 Study inclusion criteria

The PCC format for scoping reviews is an acronym for P = population; C = concept; and C = context.^43^ The review was about a population which included all individuals engaged in the health‐care system as patients, family members of patients or health‐care consumers (no age criteria). The concepts were twofold, but both included the focus on safety actions as distinct from caring for one's health. First, it was the patients’ and families’ attitudes, opinions and beliefs about taking an active role to ensure safe care at the direct care level. Second, it was the patients’ and family members’ behaviours or actions that demonstrated active engagement and involvement to prevent harm at the direct care level. The safety behaviours or actions were either self‐determined or directed as part of a study intervention. The review included evidence of whether patients and family members performed requested safety behaviours, or if they demonstrated self‐determined strategies, to prevent harm while receiving health care. The context included all sectors of health care including acute care, ambulatory care and long‐term care. Home settings (people's own home) were seen as a separate context and as such were excluded, unless they were included as part of multiple sites in a relevant paper. Direct care level was defined as any clinical care interaction, as based on the study framework.^4^

### 3.4 Types of literature

This review included systematic reviews, quantitative studies, qualitative studies, mixed‐methods studies, scoping reviews, literature reviews, quality improvement projects, as well as opinion and discussion papers. Study or systematic review protocols were also included in order to give perspective on the interest in this topic and anticipated future results. It is acknowledged that some may not be actioned into full reviews but this does reflect current research activity. Studies published in abstract form (such as conference proceedings) were not included.

### 3.5 Search strategy

A three‐step search strategy was used that considered published and unpublished papers written in English. An initial limited search of MEDLINE, CINAHL and EMBASE was undertaken followed by analysis of the text words contained in the title and abstract and the index terms used to describe the article. A second search using all identified keywords and index terms was undertaken across all included databases. Third, the reference lists and bibliographies of select reports and articles were searched for additional studies, and selections made based on title. Papers written only as abstracts were excluded. Two separate search strategies for the two research questions were conducted given each had a different focus. The databases searched (during 2016‐2019) included the following: JBI (Joanna Briggs Institute) Evidence Synthesis (1998‐2019); PsycINFO (1806‐2019); CINAHL (1981‐2019); MEDLINE (1946‐2019); EMBASE (1947‐2019); and ProQuest Dissertations and Theses Global (1861‐2019). Table 1 is a list of the initial keywords used for the first search about patient attitudes and beliefs, as well as the second search on patient behaviours.

**Table 1:** Initial keywords for scoping review search strategy

Initial keywords used for the first search about Patient Attitudes			
Patients/Family	Attitudes	Involvement	Safety
*patients; *family; *siblings; *parents	attitude; beliefs; opinions; patient perception; patient perspective; values; understanding	patient participation; role; consumer participation; patient involvement; contribution to health care; patient advocacy; consumer advocacy; advocacy	safety; patient safety; medical errors; safety management; adverse events; vigilant
Initial keywords used for the second search about Patient Behaviours			
Patients/Family	Behaviours		Safety
*patients; *family; *siblings; *parents	behaviours; self‐efficacy; patient education; patient empowerment; patient participation; consumer participation; patient involvement; patient engagement; patient advocacy; speaking up		patient safety; health‐care error—prevention and control; safety management; adverse health‐care event; handwashing; medication safety; surgical safety; infection control—prevention and control; patient handoff; safeguarding

Note

The terms within each column were entered using 'OR', and the full sets of terms in each were combined across columns using “AND”. “*exp” used with these terms to provide full scope of definition.

The review also included searching of organizational, governmental and advocacy group websites, as well as communications with patient safety experts for relevant papers and texts. The websites of 44 organizations (13 local and provincial organizations; 11 national organizations; and 20 international organizations [eg Institute for Healthcare Improvement, World Health Organization]) were searched focusing on the study objectives (see Appendix S1 for the site listing). In the searching of websites, given their design, it was most effective to search using general terms, such as “patient safety” or “patient beliefs on role in safety”, or as based on the provided categories and headings.

### 3.6 Procedure

The associated university research ethics board granted ethics approval for the multi‐phase study, which included this scoping review (HSREB 6007637, NURS‐299‐12).

### 3.7 Data collection and extraction

A standard approach was used to extract quantitative, qualitative and textual data from the literature about patients’ and families’ attitudes and behaviours related to their role in ensuring safe care. For all papers, general items, such as author(s), location of first author, title of document, name of organization or association (as relevant) and year of publication, were extracted. As relevant, data extraction also included, although not limited to, methodology, methods (eg interventions, sample, setting, data collection methods) and findings.

### 3.8 Analysis

The analysis of the extracted data from the papers (formatted as tables) for this phase occurred in several ways. A descriptive approach was taken to determine the quantity of publications by type, author, location of author, year of publication and topic (ie attitudes versus behaviours; behaviours were further sub‐divided into specific categories). Additionally, and as applicable, the results from the obtained literature were examined as a collective (published and unpublished papers together) using a content analysis approach for overall themes or patterns. This analysis was guided by the process as outlined by Miles, Huberman and Saldana,^45^ including an initial coding process, followed by second cycle categorization into the larger groupings/themes. The results of this review are presented in narrative form. Study designs are reported as published by the author(s).

## 4 RESULTS

**Figure 1:**
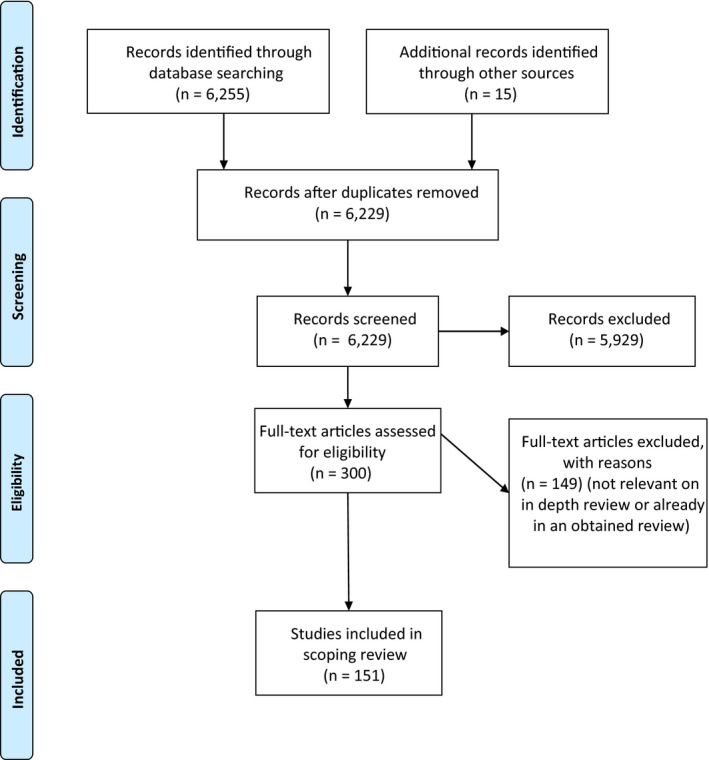
PRISMA diagram

This review includes a final set of 151 individual papers: 35 about Patient Attitudes and 125 about Patient Behaviours (Note: nine papers were in both sets given their multi‐focus). A PRISMA diagram,^46^ as reflective of the search strategy for this review, is presented in Figure 1.

Of the 151 papers included in the final set of this review, there were 13 systematic reviews; one scoping review; six systematic review or study protocols; 53 quantitative studies; 29 qualitative studies; eight mixed‐methods studies; eight literature reviews; 29 opinion or discussion papers; and five quality improvement projects. Table 2 depicts a listing of the number of publications by the first author's location, and Table 3 provides the distribution of the publication dates.

**Table 2:** Scoping review final set: by location of first author

Location	Number of publications (n = 151)
United States of America	52
United Kingdom	29
Australia	18
Canada	16
Switzerland	8
China	6
Denmark	4
Korea/Republic of Korea	3
Germany	2
Italy	2
Sweden	2
Belgium	1
Czech Republic	1
Finland	1
Iran	1
Israel	1
Japan	1
Norway	1
Singapore	1
Spain	1

**Table 3:** Scoping review final set: publication date distribution

Year of publication	Number of publications (n = 151)
2019	8
2018	14
2017	21
2016	11
2015	9
2014	17
2013	14
2012	10
2011	14
2010	12
2009	2
2008	2
2007	7
2006	3
2005	0
2004	3
2003	3
2002	1
2001	0
2000	0

### 4.1 Descriptive summary: Patient attitudes

Thirty‐five papers about patient attitudes or feelings about being involved to protect themselves when receiving health care were identified (see Appendix S2). This included three systematic reviews, eight quantitative studies, 13 qualitative studies, one mixed‐method study, one literature review, nine text and opinion pieces, and one study protocol. Publication dates ranged from 2004 to 2019 [most in: 2011 (n = 6)]. The countries of the first authors of the publications were as follows: United States of America (n = 13), United Kingdom (n = 9), Switzerland (n = 4), Canada (n = 3), Australia, Czech Republic, Denmark, Iran, Korea and Sweden (n = 1).

Of those 35 papers, three were systematic reviews.^47^, ^48^, ^49^ While the McVeety and colleagues review (n = 14 qualitative studies) was primarily about the patients’ and family members’ perspectives having encountered an adverse event, all three reviews addressed patients’ and family members’ feelings about safeguarding and beliefs about safety actions. The combined total of included studies for the three reviews was 79, with a total overlap of 24 studies. One literature review^50^ also contained some of the same articles (n = 4) as those in the Doherty and Stavropoulou systematic review.^47^ In the qualitative review by McVeety and colleagues,^48^ the number of participants ranged from 10 to 172 and included interviews (individual and focus group) in the hospital and home setting, as well as part of conference workshops. The review by Doherty and Stavropoulou^47^ included studies that had a range of participants from 4 to 2,078 patients, and included patients who were discharged from a hospital, as well as ambulatory patients. The Schwappach^49^ review sample sizes ranged from 72 to 2,078 participants and included a variety of methods including face‐to‐face interviews, focus groups, and mailed or telephone surveys. The remaining 31 papers in this set of 35 were not included in these three systematic reviews but are outlined as follows.

Thirty‐one papers about patient attitudes contained the following study designs. (a) *Nine quantitative studies*: The most common methodology was cross‐sectional exploratory studies using a survey with sample sizes ranging from 50 to 1,053 patients, including medical‐surgical patients, oncology patients and community dwelling seniors. (b) *Eleven qualitative studies*: Typically focus groups (range of 2‐8) or interviews were used, and participants included those with a recent overnight hospital admission as well as hospitalized oncology patients. Several ethnographic studies^51^, ^52^ that included observations as well as interviews were conducted. (c) *One mixed‐method study* was about patient attitudes.^53^ In this Canadian study, survey methods and focus groups were conducted with patients (217 survey responses and 10 in focus groups). (d) One study protocol for an exploratory qualitative study about patient and provider perspectives about the role of patients in safe care was also found.^54^ (e) *Nine text and opinion papers*: These items include books, opinion articles and a white paper. The authors are from the United Kingdom, United States of America, Canada and Switzerland. The publications are either authored by patients or reflect their stories and narratives using quotes.

### 4.2 Descriptive summary: Patient behaviours

One hundred twenty‐five papers related to patient behaviours, either generally or specific actions that patients are involved in, both independently or when directed by others (eg researchers; providers), to ensure their safe care. This included 12 systematic reviews [handwashing; patient behaviours—general; patient handover; medication safety; technology] [number of studies included in the reviews ranged from six to 68], 44 quantitative studies, 23 qualitative studies, eight mixed‐method studies, one scoping review, eight literature reviews, five systematic review or study protocols, 20 text and opinion pieces, and five quality improvement projects. The publication dates ranged from the years 2002 to 2019. The country of the first author of the papers was predominantly the United States of America, the United Kingdom, Australia or Canada.

The 125 papers related to patient behaviours were categorized according to specific topics for ease of reporting and to discern the range of study focus. Table 4 is a list of those categories, and the type of publication and date range for each. Appendix S3 is a detailed listing of the papers included in this subset. The findings between published and unpublished papers in many cases were complementary (dependent on topic), and although not included in this article, the retrieved grey literature about patient safety advisories/tips was immense.

**Table 4:** Scoping review subset—patient behaviours by study design

Category	Number in set	Publication data range	Study design or publication type								Quality improvement project
			Systematic review	Quantitative	Qualitative	Mixed‐methods	Scoping review	Literature review	Systematic review or study protocol	Opinion/white paper	
Handwashing	29	2006‐2019	2	22				1	1		3
Patient Behaviours*—*General	26	2002‐2018	5	4	3	2		2		10	
Patient Handover	15	2011‐2019	2	1	6	2			2	1	1
Medication Safety	13	2006‐2019	2	3	4	2		2			
Communication	8	2003‐2018		3	3					2	
Patient Reporting	5	2007‐2017		3		1			1		
Advocacy—Parents/Others	5	2004‐2018			4					1	
Ambulatory and Primary Care	4	2010‐2017					1	1		2	
Health Literacy and Safety Tips	4	2003‐2012		3						1	
Health‐care–associated Infections	3	2015‐2016		1	1						1
Surgical Safety	3	2010‐2011		1						2	
Technology	3	2003‐2019	1	1	1						
Rapid Response Team Activation	2	2014, 2018			1				1		
Diagnostic Imaging/X‐ray Imaging	2	2013, 2017		1				1			
Newborn Fall Prevention	1	2018		1							
Advocacy—General	1	2013								1	
Blood Transfusion	1	2011						1			
Total	125		12	44	23	7	1	8	5	20	5

## 5 OVERALL THEMES—PATIENT ATTITUDES AND BEHAVIOURS

### 5.1 Patient attitudes

Three patterns emerged from the 35 papers about patients’ and family members’ attitudes and beliefs toward having an active role in promoting their safe care.

#### 5.1.1 Lack of evidence about patient attitudes

The first pattern is the paucity of evidence specifically about patients’ attitudes toward their role generally in regard to safety at the direct care level. Most investigators have focused on a particular dimension of harm prevention, such as patients’ attitudes toward and comfort in asking providers about handwashing (eg four of 13 studies reported in Schwappach^49^ review; Davis et al^55^), or intervention strategies,^56^ and made inferences from those results to patient safety overall. Those primary studies with a more purposeful focus on understanding patients’ attitudes and thoughts about whether they have a role in safety at the direct care level were few in number,^24^, ^57^, ^58^, ^59^, ^60^ and were sometimes combined with general care activities,^59^, ^61^ or focused on parents.^62^ The general findings from these investigations included viewing their role in safety “as a right, not an obligation”,^58^ to seeing their role as a “shared responsibility” with providers.^60^ Most importantly, the mapping of this literature identified limitations in our current understanding in this regard, both in terms of depth of evidence and clarity of concepts.

#### 5.1.2 Patients’ opinions about their role vary

Second, a pattern emerging from the existing evidence indicates patients have varied opinions and perceptions about having an active role in error prevention.^47^, ^49^, ^51^ There is no consistent, unequivocal agreement among patients and family members about their level of involvement or role in safety.^47^, ^49^, ^51^ For some patients, they see their role as a passive one and that safety is not their responsibility. For others, they believe patients must participate to help with harm prevention.^47^, ^49^, ^51^ Generally, different factors, values, understanding and experiences influenced individuals’ attitudes toward their role and involvement in safety in different ways.

There are individuals who believe that safety is the obligation of the health‐care provider and that patients do not have that responsibility.^51^, ^63^ As found by some investigators, when the patient (philosophically) views their role as a passive one, they are not willing to be involved in safety and are described as having more submissive attitudes toward safety,^47^ as well as believe their only focus is to get well.^51^ In one qualitative study, participants expressed fear that the responsibility of safe care would shift to patients and that the factors contributing to errors were beyond their control, and therefore, they could have little influence.^56^

There are individuals who have a different view, believing that patients should be engaged in ensuring safe care,^49^, ^64^, ^65^ see the benefit of it,^63^ and that they can effectively contribute to safety.^53^, ^66^ Patients have expressed the viewpoint that they know themselves best, and therefore need to be engaged and involved in sharing that knowledge and ensuring it is not dismissed.^67^ In a 2011 cross‐sectional survey^68^ of 1,053 patients, 95% agreed that patients should be educated about how to prevent errors, while other investigators have reported that having the opportunity to discuss safety issues with care was rated favourably by patients.^69^ In a small sample of 50 internal medicine patients, 90% wanted to review their medication list for accuracy and 94% believed patient involvement and review of medications had the potential to reduce error.^70^ Even when patients had difficulty understanding the specifics of what safety engagement involved, for this group of participants, the willingness to participate, whatever the underlying motivation or inspiration, was evident.^51^

#### 5.1.3 Patient belief may differ from their action

The third pattern relates to the second, specifically to those patients and consumers who believe they have a role in promoting safe care. While patients may indicate support and favourable attitudes toward safety involvement generally, intention to act or actual behaviours may be different.^49^, ^60^ The Theory of Planned Behaviour^71^ has been used as a conceptual framework by several investigators when assessing patient attitudes about engagement in safety.^49^, ^60^, ^63^, ^68^ The premise of the theory is that an intention to act is influenced by the attitude toward that behaviour, the perceived influence of others and the perception of ease or difficulty of the behaviour.^71^ Behavioural intent is predictive of behaviour.^71^ Schwappach^49^ suggested that patient engagement in patient safety can be viewed as “…a special case of health‐promoting behaviour” (p. 122), as such, the theory can be a useful one in explaining and predicting involvement. In conceptualizing patient safety in this way, it is helpful in identifying that a patient's positive attitude about safety engagement does not necessarily equate to actual action. A number of investigators have demonstrated that only considering attitude is not sufficient and that other elements are influential, including why a patient will ask about their medications but not a provider about handwashing.^47^, ^49^, ^53^, ^60^ The 21‐item tool to measure views about safety tasks by Elder et al^72^ is an example of how investigators are not only asking individuals how comfortable they are with certain activities, but how often they have completed them. Overall, it is evident that one must be cautious of oversimplifying a positive attitude about patient engagement in patient safety without consideration and examination of other potential influencing factors and qualifiers.

### 5.2 Patient behaviours

Four patterns emerged reflecting patients’ and family members’ behaviours and actions in promoting their safe care.

#### 5.2.1 Escalation in patient engagement research

The first is the increased interest in this topic in recent years, and the evolving range and diversity of how patients are being involved to support the safety of their care. The action of patients asking providers whether they have washed their hands has been a predominant focus of research in this field.^49^ Davis and colleagues^73^ conducted a systematic review of the evidence on the effectiveness of strategies that increase patient participation in reminding providers about hand hygiene. Their review included a total of 28 articles, and while they reported a number of strategies being examined to increase this type of patient involvement, they found the studies lacked methodological rigour, and cautioned that because most studies were designed to examine patients’ intention about asking, their actual behaviour may be different.

In addition to asking providers about handwashing, researchers have investigated how patients can participate and take action to support safe care related to other care processes, including patient handover^74^, ^75^, ^76^, ^77^, ^78^, ^79^ and medication safety.^80^, ^81^, ^82^, ^83^, ^84^, ^85^ More recently, researchers have examined areas such as diagnostic imaging^86^ and primary care^87^, ^88^ to determine whether there are opportunities for patients to participate in safe care in these settings. It seems clear that, with the increasing attention given to patient and family engagement generally^4^, ^89^ and the continued focus on harm prevention, there is greater interest to determine how patients can engage and participate in safety‐specific practices.

#### 5.2.2 Patients are engaged

The second pattern was the indication that patients are engaging in behaviours that promote safe care, either independently in their own ways^56^, ^81^, ^90^, ^91^, ^92^ or as requested to varying degrees^80^, ^93^, ^94^, ^95^ and that they see and are aware of safety practices and strategies occurring in the health‐care environment.^96^ The literature is limited, but researchers have reported that patients have developed strategies to protect themselves, such as taking notes; asking a family member to ask a question on their behalf; seeking information from the Internet; talking with other patients; learning by listening to providers educate each other; and speaking up when concerned,^91^, ^92^, ^93^ and may not even recognize the activities they do as safety measures.^51^ Additional research is needed, but current evidence suggests that patients can and do perform actions to promote their safe care, and positive outcomes related to their involvement have been described.^97^ Australians Hor and colleagues^98^ believe that patients are already involved and that instead of asking whether patients *ought* to be involved with safety, we should be asking how we can make patient‐provider collaborations for safety more effective.

#### 5.2.3 A focus on influencing and impeding factors to engagement

A third pattern was patients’ and family members’ behaviours about promoting safe care. Investigators have examined factors that influence or impede patients’ participation in safety initiatives.^47^, ^94^ Factors that may support and enhance patient involvement in safety include the following: perceived risk, provider encouragement, perceived self‐efficacy^47^, ^73^ and health‐care setting^50^ (eg primary care is seen as key in developing trusting relations with implications to safety^99^ and is seen as more feasible to engage,^88^ yet seen as paramount for family members in hospital settings^62^). Issues that may negatively influence an individual's ability to engage in activities to support safe care include the following: severity of one's illness, perception of staff work pressure, lack of awareness of the benefit of their involvement, engaging in a task perceived as challenging authority (versus factual, information‐sharing tasks) and belief that one's role should be passive.^47^, ^49^, ^94^ There is recognition that there will be different motivators and inhibitors that will affect patients’ behaviours related to participating in the safety aspects of their care, and while understanding the motivators and reasons for engagement was not a focus of this review, further unpacking influencing factors such as whether one's preference to engage is anxiety or fear‐based that safe care could be compromised versus acting based on preference alone is warranted.

#### 5.2.4 The role of family

The final pattern was the role of family in advocating for and protecting the well‐being of another. Parents whose children are requiring health care report and demonstrate a vigilance and protectiveness to ensure they are safe.^36^, ^90^, ^100^, ^101^ Of 130 parents of hospitalized children, 63% agreed or strongly agreed that they needed to safeguard their child against potential errors.^101^ Hurst's^36^ critical ethnography of 12 mothers of hospitalized premature babies wrote of how mothers’ primary action was to watch over and observe their baby and the care processes. Parents advocated on behalf of their child and were prepared to ask about and challenge processes if needed^90^; however they were also sensitive and cautious as to what and how they questioned.^36^, ^62^ Parents viewed their role as a protector—a responsibility described as “both their right and their job” (p. 321).^62^ In the adult setting, the family role was equally important and members as engaged. Family members acted as observers, noted in the study by Kim,^102^ where family members report watching care practices, such as provider handwashing (63% report they agree/strongly agree that they observe provider handwashing; n = 173). Family members reported speaking up about worrying symptoms they see exhibited in their loved one and of their comfort^10^ when action is taken by the provider as a result.^103^ Patients also called on family members to assist them in ensuring safe care, including asking a family member to question providers on their behalf,^93^ and the role of family members increase if individuals are too ill or cognitively impaired.^103^ Investigators have included family members in studies about safety and error‐prevention strategies,^104^ though the investigation of family members and their behaviours related to ensuring safe care as a primary focus is limited. Though not the intended focus of this study, the responsibility of nurses to advocate for patients and to support and encourage them in their own advocacy is also an important consideration.

## 6 DISCUSSION

There are important considerations as a result of this review. First, this review provides confirmation of the *interest internationally*, in varying ways and degrees, about patient and family engagement in safety. The number of publications written in English in peer‐reviewed journals suggests that many are investigating, writing and commenting on patient involvement in safety. The results of this analysis are one indication, with 21 publications from 2000 to 2009, increasing to 130 from 2010 to 2019. It could be as a result of both overall efforts in patient engagement in health care generally, and in harm prevention. Those organizations that have patient safety as their mandate have also expanded their focus to include patient involvement, such as the World Health Organization's *Patients for Patient Safety* (an advocacy programme to elevate and profile the rights and viewpoints of patients and families regarding their health, and including collaboration with providers in advancing quality person‐centred health care^105^). Additionally, the advantage of mapping this topic provides insight into how patient safety engagement is being parsed, defined or promoted—not always leading to success. The most common focus regarding behaviour was handwashing and specifically, encouraging patients to ask providers whether they have washed their hands prior to providing care. Given substantive limitations in establishing consistent and sustained effectiveness and success rates, continued attempts to engage patients in this area are questionable, or at the very least, a focus on patient‐identified strategies may have more beneficial effects on the rates.

Second, there is a *need to better understand patients’ attitudes* and beliefs about engagement at the direct care level in safety holistically across the continuum of care and to include a more specific focus on family members’ opinions about how they feel about having a role in safeguarding. Investigators have made inferences from one or two safety practices (eg asking about handwashing) and have generalized to how patients feel on all aspects of personal safety. Also, there needs to be clarity, and consistency in meaning and approaches to distinguishing how patients *believe they should be, want to be, are willing to be, are able to be* and *need to be* involved in safety. This also includes how they report being and are observed to be involved in safety, and all of which may vary across the continuum of the patient's condition. Related to this is awareness that beliefs and attitudes may not translate to actions—saying one agrees philosophically with the premise does not mean they will action that behaviour in reality. Conversely, it may be that some consumers do not fundamentally agree that patients should have a role but engage because they have been asked to be part of the process. It is these complexities and nuances that make investigating this topic a difficult one. Overall, based on the review results, while there is the belief and attitude of many patients and family members that patients should and want to participate in partnering to ensure safe care, safety is principally seen as the responsibility of providers.

Third, most of the existing evidence is about hospitalized adult medical‐surgical patients, and *primary care has not been* extensively studied. Further, the patients’ and family members’ role in supporting safe care is different and evolves or changes during the continuum of care (eg from emergency to critical care to a medical unit) and in different settings (ambulatory versus inpatient) and this context is important to understand. Additionally, it is important to ensure the best study methodologies, dependent on the research study question(s), are used to investigate these complex topics, and not approached as an add‐on or out of convenience (eg survey)—an observation of some existing literature. It is offered that approaches such as an exploratory sequential mixed‐methods design or multi‐phased studies may best address this complexity, but integral to this work must be rigorous designs that capture the richness and depth of patient and family insights.

Fourth, the findings of the scoping review support the concept that there are *many issues to consider* when one examines patient engagement in patient safety at the direct care level. Additionally, these issues can change and evolve over time, and are unique to each patient. The review by Davis and colleagues^50^ about factors affecting participation in patient engagement in safety illustrated similar concepts to the qualitative study of this multi‐phase research,^3^ including patient‐related characteristics, illness‐related aspects, health‐care professional‐related characteristics, health‐care–related characteristics and task‐related characteristics. However, it does not address issues such as patient's temperament, the choices and decisions they are making about their circumstance or specific situations, or safety behaviours that patients engage in on their own. As well, previous experience with a safety incident did not consistently mean patients were engaged. Some investigators have found inconsistency in demographic characteristics,^47^, ^106^ while others suggest predictable influences.^29^

Finally, investigators have typically examined safety practices that they have identified, *without consideration of patient preferences and without using a participatory action research approach*. In most cases, the uptake of these behaviours has been variable and lacking consistency. Further, in many cases, an indirect finding has been that patients engage in strategies of their own. A more effective approach when considering patient engagement in patient safety may be to determine patients’ understandings and preferences at the point of contact with the health‐care system and strengthen and enhance those self‐identified strategies. An efficient, user‐friendly mechanism for determining this will be required. Vincent^107^ and Spath^33^ write of the complexity of involvement and the importance of discerning patient preferences, and it is offered that greater emphasis needs to be taken in this regard to most effectively involve patients and family members in ways that are right for them and in support of their safe care at the direct care level.

### 6.1 Study strengths and limitations

It is offered that, as a component of a larger investigation, the strength of this study is its depth and breadth, particularly related to the focus on both attitudes and behaviours across the continuum of care, and cross‐referenced with findings from the qualitative study phase.^3^ To our understanding, no other review has been conducted with this comprehensive approach. It provides perspective internationally about initiatives and efforts that are underway to engage patients in different elements of safety, and illuminated the gaps that remain. It is acknowledged that a study limitation may include the oversight of papers not identified in the applied search strategy.

## 7 CONCLUSION

This review was about mapping what is known of patients’ and families’ attitudes regarding their role in safety at the direct care level, as well as their reported behaviours in support of their safe care. The review included 151 papers, and among the findings included patients’ belief in having a role in safer care (although not for everyone), with indication of the need for further investigation in this regard, as well as degrees of variability in taking action. The review also provided perspective of the rapidly evolving interest in this topic, particularly as it relates to behaviours generally, and more specifically about the involvement of patients in asking providers about handwashing, although involving patients in the research process and specifically in patient‐identified engagement safety strategies is needed. We must appreciate that many patients are engaged while recognizing their own and system limitations, and better position ourselves as researchers, policymakers and providers in understanding and implementing approaches collaboratively as relevant and feasible.

## CONFLICT OF INTEREST

None of the authors have any conflict of interest to declare.

## Supporting information

Appendix S1

Appendix S2

Appendix S3

## Data Availability

Data sharing is not applicable to this article as no new data were created or analysed in this study.

## References

[1] Panagioti M , Khan K , Keers RN , et al. Prevalence, severity, and nature of preventable patient harm across medical care settings: systematic review and meta‐analysis. BMJ. 2019;366:l4185. 31315828 10.1136/bmj.l4185 PMC6939648

[2] Gandhi TK , Kaplan GS , Leape L , et al. Transforming concepts in patient safety: a progress report. BMJ Qual Safety. 2018;27(12):1019‐1026. 10.1136/bmjqs-2017-007756 PMC6288701 30018115

[3] Duhn L , Medves J . A 5‐facet framework to describe patient engagement in patient safety. Health Expect. 2018;21(6):1122‐1133. 30160006 10.1111/hex.12815 PMC6250877

[4] Carman KL , Dardess P , Maurer M , et al. Patient and family engagement: a framework for understanding the elements and developing interventions and policies. Health Affairs (Project Hope). 2013;32(2):223‐231. 23381514 10.1377/hlthaff.2012.1133

[5] Duhn L . A comparison of patient safety perspectives of patients, families and healthcare professionals. In: Queen's University; 2011.

[6] Bernstein M , Potvin D , Martin DK . A qualitative study of attitudes toward error in patients facing brain tumour surgery. Can J Neurol Sci. 2004;31(2):208‐212. 15198445 10.1017/s0317167100053841

[7] Burroughs TE , Waterman AD , Gallagher TH , et al. Patients' concerns about medical errors during hospitalization. Jt Comm J Qual Patient Saf. 2007;33(1):5‐14. 17283937 10.1016/s1553-7250(07)33002-x

[8] Dowell D , Manwell L , Maguire A , et al. Urban outpatient views on quality and safety in primary care. Longwoods Rev. 2005;3(1):2‐8. 10.12927/hcq.2005.17230 15828567

[9] Elder NC , Jacobson CJ , Zink T , Hasse L . How experiencing preventable medical problems changed patients' interactions with primary health care. Ann Fam Med. 2005;3(6):537‐544. 16338918 10.1370/afm.346 PMC1466948

[10] Entwistle VA , McCaughan D , Watt IS , et al. Speaking up about safety concerns: multi‐setting qualitative study of patients' views and experiences. Qual Safety Health Care. 2010;19(6):e33. 10.1136/qshc.2009.039743 21127092

[11] Hupcey JE . Feeling safe: the psychosocial needs of ICU patients. J Nurs Scholarsh. 2000;32(4):361‐367. 11140200 10.1111/j.1547-5069.2000.00361.x

[12] Kuzel AJ , Woolf SH , Gilchrist VJ , et al. Patient reports of preventable problems and harms in primary health care. Ann Fam Med. 2004;2(4):333‐340. 15335132 10.1370/afm.220 PMC1466690

[13] Mazor KM , Goff SL , Dodd KS , Velten SJ , Walsh KE . Parents' perceptions of medical errors. J Patient Saf. 2010;6(2):102‐107. 22130352 10.1097/PTS.0b013e3181ddfcd0

[14] National Patient Safety Foundation at the AMA . Public Opinion of Patient Safety Issues: Research Findings. In. Boston, MA1997.

[15] Rathert C , Brandt J , Williams ES . Putting the 'patient' in patient safety: a qualitative study of consumer experiences. Health Expect. 2011;15(3):327‐336. 21624026 10.1111/j.1369-7625.2011.00685.x PMC5060619

[16] Saxton M , Curry MA , Powers LE , Maley S , Eckels K , Gross J . “Bring My Scooter So I Can Leave You” A Study of Disabled Women Handling Abuse by Personal Assistance Providers. Violence Against Women. 2001;7(4):393‐417.

[17] Saxton M , McNeff E , Powers L , Curry MA . We're all little John Waynes: a study of disabled men's experience of abuse by personal assistants. J Rehabil. 2006;72(4):3.

[18] Schwappach DLB . "Against the silence": development and first results of a patient survey to assess experiences of safety‐related events in hospital. BMC Health Serv Res. 2008;8:59. 18366707 10.1186/1472-6963-8-59 PMC2279127

[19] Schwappach DLB , Wernli M . Am I (un) safe here? Chemotherapy patients' perspectives towards engaging in their safety. Qual Safety Health Care. 2010;19(5):e9. 10.1136/qshc.2009.033118 20427299

[20] Vanderheyden L , Northcott H , Adair C , et al. Reports of preventable medical errors from the Alberta Patient Safety Survey 2004. Healthcare Quart. 2005;8(sp):107‐114. 10.12927/hcq..17674 16334082

[21] Weingart SN , Price J , Duncombe D , et al. Patient‐reported safety and quality of care in outpatient oncology. Jt Comm J Qual Patient Saf. 2007;33(2):83‐94. 17370919 10.1016/s1553-7250(07)33010-9

[22] Wolosin RJ , Vercler L , Matthews JL . Am I safe here?: improving patients' perceptions of safety in hospitals. J Nurs Care Qual. 2006;21(1):30‐40. 16340686 10.1097/00001786-200601000-00008

[23] Pandhi N , Schumacher J , Flynn KE , Smith M . Patients' perceptions of safety if interpersonal continuity of care were to be disrupted. Health Expect. 2008;11(4):400‐408. 19076668 10.1111/j.1369-7625.2008.00503.x PMC2689380

[24] Rathert C , Huddleston N , Pak Y . Acute care patients discuss the patient role in patient safety. Health Care Manage Rev. 2011;36(2):134‐144. 21317659 10.1097/HMR.0b013e318208cd31

[25] Walrath JM , Rose LE . The medication administration process: patients' perspectives. J Nurs Care Qual. 2008;23(4):345‐352. 18521044 10.1097/01.NCQ.0000323287.64020.01

[26] Agoritsas T , Bovier PA , Perneger TV . Patient reports of undesirable events during hospitalization. J Gen Intern Med. 2005;20(10):922‐928. 16191139 10.1111/j.1525-1497.2005.0225.x PMC1490233

[27] Buetow S , Henshaw J , Bryant L , O'Sullivan D . Medication timing errors for Parkinson's disease: perspectives held by caregivers and people with Parkinson's in new zealand. Parkinson's Dis. 2010;2010:432983. 20975777 10.4061/2010/432983 PMC2957227

[28] Swahnberg K , Wijma B , Hearn J , Thapar‐Björkert S , Berterö C . Mentally pinioned: men's perceptions of being abused in health care. Int J Mens Health. 2009;8(1):60.

[29] Davis RE , Koutantji M , Vincent CA . How willing are patients to question healthcare staff on issues related to the quality and safety of their healthcare? An exploratory study. Qual Saf Health Care. 2008;17(2):90‐96. 18385400 10.1136/qshc.2007.023754

[30] Davis RE , Sevdalis N , Vincent CA . Patient involvement in patient safety: How willing are patients to participate? BMJ Qual Saf. 2011;20(1):108‐114. 10.1136/bmjqs.2010.041871 21228083

[31] Hibbard JH , Peters E , Slovic P , Tusler M . Can patients be part of the solution? Views on their role in preventing medical errors. Med Care Res Rev. 2005;62(5):601‐616. 16177460 10.1177/1077558705279313

[32] Marella WM , Finley E , Thomas AD , Clarke JR . Health care consumers' inclination to engage in selected patient safety practices: a survey of adults in Pennsylvania. J Patient Saf. 2007;3(4):184‐189.

[33] Spath PL . Safety from the patient’s point of view In: Spath PL, ed. Engaging Patients as Safety Partners: A Guide for Reducing Errors and Improving Satisfaction. Chicago, IL: Health Forum Incorporated; 2008:1‐40.

[34] Waterman AD , Gallagher TH , Garbutt J , Waterman BM , Fraser V , Burroughs TE . Brief report: Hospitalized patients' attitudes about and participation in error prevention. J Gen Intern Med. 2006;21(4):367‐370. 16686815 10.1111/j.1525-1497.2005.00385.x PMC1484719

[35] Foundation TKF . Update on consumers’ views of patient safety and quality information. 2008; http://www.kff.org/kaiserpolls/posr101508pkg.cfm

[36] Hurst I . Vigilant watching over: mothers' actions to safeguard their premature babies in the newborn intensive care nursery. J Perinat Neonatal Nurs. 2001;15(3):39‐57. 11785577 10.1097/00005237-200112000-00005

[37] Johnstone M‐J , Kanitsaki O . Engaging patients as safety partners: some considerations for ensuring a culturally and linguistically appropriate approach. Health Policy (Amsterdam, Netherlands). 2008;90(1):1‐7. 10.1016/j.healthpol.2008.08.007 18829130

[38] Jeffs L , Affonso DD , Macmillan K . Near misses: paradoxical realities in everyday clinical practice. Int J Nurs Pract. 2008;14(6):486‐494. 19126078 10.1111/j.1440-172X.2008.00724.x

[39] Parnes B , Fernald D , Quintela J , et al. Stopping the error cascade: A report on ameliorators from ASIPS collaborative. Qual Saf Health Care. 2007;16:12‐16. 17301195 10.1136/qshc.2005.017269 PMC2464918

[40] Unruh KT , Pratt W . Patients as actors: the patient's role in detecting, preventing, and recovering from medical errors. Int J Med Informatics. 2006;76(Suppl 1):S236‐244. 10.1016/j.ijmedinf.2006.05.021 16829180

[41] Entwistle VA , Mello MM , Brennan TA . Advising patients about patient safety: current initiatives risk shifting responsibility. Jt Comm J Qual Patient Saf. 2005;31(9):483‐494. 16255326 10.1016/s1553-7250(05)31063-4

[42] Weingart SN , Morway L , Brouillard D , et al. Rating recommendations for consumers about patient safety: sense, common sense, or nonsense? Jt Comm J Qual Patient Saf. 2009;35(4):206. 19435160 10.1016/s1553-7250(09)35028-x

[43] Peters MDJ , Godfrey CM , Khalil H , McInerney P , Parker D , Soares CB . Guidance for conducting systematic scoping reviews. Int J Evid Based Healthc. 2015;13(3):141‐146. 26134548 10.1097/XEB.0000000000000050

[44] Arksey H , O’Malley L . Scoping studies: towards a methodological framework. Int J Soc Res Methodol. 2005;8(1):19‐32.

[45] Miles MB , Huberman AM , Saldana J . Qualitative Data Analysis: A Methods Sourcebook, 3rd edn Thousand Oaks, CA: Sage Publications; 2014.

[46] Moher D , Liberati A , Tetzlaff J , Altman DG , Prisma G . Preferred reporting items for systematic reviews and meta‐analyses: the PRISMA statement. PLoS Medicine. 2009;6(7):e1000097. 19621072 10.1371/journal.pmed.1000097 PMC2707599

[47] Doherty C , Stavropoulou C . Patients' willingness and ability to participate actively in the reduction of clinical errors: a systematic literature review. Soc Sci Med. 2012;75(2):257‐263. 22541799 10.1016/j.socscimed.2012.02.056

[48] McVeety J , Keeping‐Burke L , Harrison MB , Godfrey C , Ross‐White A . Patient and family member perspectives of encountering adverse events in health care: a systematic review. JBI Database of System Rev Implement Rep. 2014;12(7):315‐373.

[49] Schwappach DLB . Review: engaging patients as vigilant partners in safety: a systematic review. Med Care Res Rev. 2010;67(2):119‐148. 19671916 10.1177/1077558709342254

[50] Davis RE , Jacklin R , Sevdalis N , Vincent CA . Patient involvement in patient safety: what factors influence patient participation and engagement? Health Expect. 2007;10(3):259‐267. 17678514 10.1111/j.1369-7625.2007.00450.x PMC5060404

[51] Martin HM , Navne LE , Lipczak H . Involvement of patients with cancer in patient safety: a qualitative study of current practices, potentials and barriers. BMJ Qual Saf. 2013;22(10):836‐842. 10.1136/bmjqs-2012-001447 23754594

[52] Garfield S , Jheeta S , Husson F , et al. The Role of Hospital Inpatients in Supporting Medication Safety: A Qualitative Study. PLoS One. 2016;11(4):e0153721. 27093438 10.1371/journal.pone.0153721 PMC4836703

[53] Bishop AC *Perceptions of Patient Safety: What Influences Patient and Provider Involvement?* Halifax, Nova Scotia, Dalhousie University; 2012.

[54] Chegini Z , Janati A , Bababie J , Pouraghaei M . The role of patients in the delivery of safe care in hospital: Study protocol. J Adv Nurs. 2019;75(9):2015‐2023. 31087572 10.1111/jan.14045

[55] Davis R , Anderson O , Vincent C , Miles K , Sevdalis N . Predictors of hospitalized patients' intentions to prevent healthcare harm: a cross sectional survey. Int J Nurs Stud. 2012;49(4):407‐415. 22098924 10.1016/j.ijnurstu.2011.10.013

[56] Pinto A , Vincent C , Darzi A , Davis R . A qualitative exploration of patients' attitudes towards the 'Participate Inform Notice Know' (PINK) patient safety video. Int J Qual Health Care. 2013;25(1):29‐34. 23175533 10.1093/intqhc/mzs073

[57] Schenk EC , Bryant RA , Van Son CR , Odom‐Maryon T . Perspectives on Patient and Family Engagement With Reduction in Harm: The Forgotten Voice. J Nurs Care Qual. 2019;34(1):73‐79. 29889721 10.1097/NCQ.0000000000000333

[58] Burrows Walters C , Duthie EA . Patients' Perspectives of Engagement as a Safety Strategy. Oncol Nurs Forum. 2017;44(6):712‐718. 29052666 10.1188/17.ONF.712-718 PMC5720142

[59] Ringdal M , Chaboyer W , Ulin K , Bucknall T , Oxelmark L . Patient preferences for participation in patient care and safety activities in hospitals. BMC Nurs. 2017;16:1‐8. 29200965 10.1186/s12912-017-0266-7 PMC5696683

[60] Walters CB .Perceptions of hospitalized oncology patients regarding involvement in their care as a patient safety strategy across a range of health literacy levels. 2013.

[61] Tobiano G , Bucknall T , Marshall A , Guinane J , Chaboyer W . Patients' perceptions of participation in nursing care on medical wards. Scand J Caring Sci. 2016;30(2):260‐270. 26036723 10.1111/scs.12237

[62] Rosenberg RE , Rosenfeld P , Williams E , et al. Parents' Perspectives on "Keeping Their Children Safe" in the Hospital. J Nurs Care Qual. 2016;31(4):318‐326. 27219828 10.1097/NCQ.0000000000000193

[63] Schwappach DLB , Wernli M . Predictors of chemotherapy patients' intentions to engage in medical error prevention. Oncologist. 2010;15(8):903‐912. 20682607 10.1634/theoncologist.2010-0117 PMC3228023

[64] Britnell M *In search of the perfect health system* . Palgrave Macmillan; 2015.

[65] Goeltz R , Hatlie MJ . Trial and error in my quest to be a partner in my health care: a patient's story. Crit Care Nurs Clin North Am. 2004;14(4):391‐399. 10.1016/s0899-5885(02)00033-3 12400630

[66] Leuthold M . Patients as partners for improving safety. World Hosp Health Serv. 2014;50(3):20‐22. 25985549

[67] Toronto PDicw, LHIN C . Meeting with patients: Their experiences and perspectives. In. Toronto, ON: Patient Destiny in collaboration with Toronto Central LHIN; 2011.

[68] Schwappach DLB , Frank O , Koppenberg J , Muller B , Wasserfallen J‐B . Patients' and healthcare workers' perceptions of a patient safety advisory. Int J Qual Health Care. 2011;23(6):713‐720. 21937585 10.1093/intqhc/mzr062

[69] Bartlova S , Tothova V , Brabcova I , Prokesova R , Kimmer D . The hospitalized patient as a partner in the survey on safe care in the Czech Republic. Neuroendocrinol Lett. 2014;35(Suppl 1):5‐10. 25433348

[70] Cumbler E , Wald H , Kutner J . Lack of patient knowledge regarding hospital medications. J Hosp Med. 2010;5(2):83‐86. 20013875 10.1002/jhm.566

[71] Ajzen I , Fishbein M . Understanding Attitudes and Predicting Social Behaviour. Upper Saddle River: Prentice‐Hall; 1980.

[72] Elder NC , Regan SL , Pallerla H , Levin L , Post D , Cegela DJ . Development of an instrument to measure seniors' patient safety health beliefs: the Seniors Empowerment and Advocacy in Patient Safety (SEAPS) survey. Patient Educ Couns. 2007;69(1–3):100‐107. 17851015 10.1016/j.pec.2007.07.007

[73] Davis R , Parand A , Pinto A , Buetow S . Systematic review of the effectiveness of strategies to encourage patients to remind healthcare professionals about their hand hygiene. J Hosp Infect. 2015;89(3):141‐162. 25617088 10.1016/j.jhin.2014.11.010

[74] Becker CA . Patient Perceptions of Bedside Shift Report: A Qualitative Case Study. Phoenix: Arizona, University of Phoenix; 2014.

[75] Drach‐Zahavy A , Shilman O . Patients' participation during a nursing handover: the role of handover characteristics and patients' personal traits. J Adv Nurs. 2015;71(1):136‐147. 24989868 10.1111/jan.12477

[76] Flink M , Hesselink G , Pijnenborg L , et al. The key actor: a qualitative study of patient participation in the handover process in Europe. BMJ Qual Saf. 2012;21(Suppl 1):i89‐i96. 10.1136/bmjqs-2012-001171 PMC3551200 23112290

[77] Jeffs L , Beswick S , Acott A , et al. Patients' views on bedside nursing handover: creating a space to connect. J Nurs Care Qual. 2014;29(2):149‐154. 24253179 10.1097/NCQ.0000000000000035

[78] McMurray A , Chaboyer W , Wallis M , Johnson J , Gehrke T . Patients' perspectives of bedside nursing handover. Collegian. 2011;18(1):19‐26. 21469417 10.1016/j.colegn.2010.04.004

[79] Wildner J , Ferri P . Patient participation in change‐of‐shift procedures: the implementation of the bedside handover for the improvement of nursing quality in an Italian hospice. J Hosp Palliat Nurs. 2012;14(3):216‐224.

[80] Heyworth L , Paquin AM , Clark J , et al. Engaging patients in medication reconciliation via a patient portal following hospital discharge. J Am Med Inform Assoc. 2014;21:e157‐162. 10.1136/amiajnl-2013-001995 PMC3957401 24036155

[81] Macdonald MT , Heilemann MV , MacKinnon NJ , et al. Confirming delivery: understanding the role of the hospitalized patient in medication administration safety. Qual Health Res. 2014;24(4):536‐550. 24598773 10.1177/1049732314524487

[82] McTier L , Botti M , Duke M . Patient participation in medication safety during an acute care admission. Health Expect. 2015;18(5):1744‐1756. 24341439 10.1111/hex.12167 PMC5060834

[83] Myhre TA . Medication Safety Practices: A Patient's Perspective. Lethbridge, AL: School of Health Sciences, University of Lethbridge; 2007.

[84] Schwappach DLB , Wernli M . Medication errors in chemotherapy: incidence, types and involvement of patients in prevention. A review of the literature. Eur J Cancer Care. 2010;19(3):285‐292. 10.1111/j.1365-2354.2009.01127.x 19708929

[85] Wright J , Emerson A , Stephens M , Lennan E . Hospital inpatient self‐administration of medicine programmes: a critical literature review. Pharm World Sci. 2006;28(3):140‐151. 17004024 10.1007/s11096-006-9014-x

[86] McDonald KM , Bryce CL , Graber ML . The patient is in: patient involvement strategies for diagnostic error mitigation. BMJ Qual Saf. 2013;22(Suppl 2):ii33‐ii39. 10.1136/bmjqs-2012-001623 PMC3786634 23893394

[87] Martin HM , Larsen J .Patient involvement in Patient Safety: A literature review about European primary care. The Danish Institute for Health Services Research for the Danish Society for Patient Safety and the LINNEAUS EURO‐PC project. 2012.

[88] Trier H , Valderas JM , Wensing M , Martin HM , Egebart J . Involving patients in patient safety programmes: A scoping review and consensus procedure by the LINNEAUS collaboration on patient safety in primary care. Pharm World Sci. 2015;21(Suppl):56‐61. 10.3109/13814788.2015.1043729 PMC4828601 26339838

[89] Carman KL , Dardess P , Maurer ME , Workman T , Ganachari D , Pathak‐Sen E . A roadmap for patient and family engagement in healthcare practice and research. *Palo Alto, CA2014 p Prepared by the American Institutes for Research under flagrant from the Gordon and Betty Moore Foundation, Dominick Frosch, Project Officer and Fellow*. 2014.

[90] Clarke JN , Fletcher PC . Parents as advocates: stories of surplus suffering when a child is diagnosed and treated for cancer. Soc Work Health Care. 2004;39(1–2):107‐127. 15774387

[91] Rance S , McCourt C , Rayment J , et al. Women's safety alerts in maternity care: is speaking up enough? BMJ Qual Saf. 2013;22(4):348‐355. 10.1136/bmjqs-2012-001295 23417732

[92] Wyer M , Jackson D , Iedema R , et al. Involving patients in understanding hospital infection control using visual methods. J Clin Nurs. 2015;24(11–12):1718‐1729. 25662176 10.1111/jocn.12779

[93] Seale H , Chughtai AA , Kaur R , et al. Ask, speak up, and be proactive: Empowering patient infection control to prevent health care‐acquired infections. Am J Infect Control. 2015;43(5):447‐453. 25952047 10.1016/j.ajic.2015.01.007

[94] Vaismoradi M , Jordan S , Kangasniemi M . Patient participation in patient safety and nursing input ‐ a systematic review. J Clin Nurs. 2015;24(5–6):627‐639. 25178172 10.1111/jocn.12664

[95] Weingart SN , Zhu J , Chiappetta L , et al. Hospitalized patients' participation and its impact on quality of care and patient safety. Int J Qual Health Care. 2011;23(3):269‐277. 21307118 10.1093/intqhc/mzr002 PMC3140261

[96] Clark PR *An Emergency Department Patient's Perception of Safety: A Dissertation* . San Antonio:TX, The University of Texas Health Science Center at San Antonio; 2010.

[97] Hall J , Peat M , Birks Y , et al. Effectiveness of interventions designed to promote patient involvement to enhance safety: a systematic review. Qual Saf Health Care. 2010;19(5):e10. 10.1136/qshc.2009.032748 20427301

[98] Hor SY , Godbold N , Collier A , Iedema R . Finding the patient in patient safety. Health. 2013;17(6):567‐583. 23349385 10.1177/1363459312472082

[99] Kingston‐Riechers J , Ospina M , Jonsson E , Childs P , McLeod L , Maxted J . Patient safety in primary care. *Edmonton AB: Canadian Patient Safety Institute and BC Patient Safety and Quality Council*. 2010.

[100] Sandlin‐Leming D . Pediatric patient safety: educating parents. J Perianesth Nurs. 2010;25(2):116‐118. 20359649 10.1016/j.jopan.2010.01.009

[101] Tarini BA , Lozano P , Christakis DA . Afraid in the hospital: parental concern for errors during a child's hospitalization. J Hosp Med. 2009;4(9):521‐527. 19653281 10.1002/jhm.508

[102] Kim M‐K , Nam EY , Na SH , et al. Discrepancy in perceptions regarding patient participation in hand hygiene between patients and health care workers. Am J Infect Control. 2015;43(5):510‐515. 25752956 10.1016/j.ajic.2015.01.018

[103] Rainey H , Ehrich K , Mackintosh N , Sandall J . The role of patients and their relatives in 'speaking up' about their own safety ‐ a qualitative study of acute illness. Health Expect. 2015;18(3):392‐405. 23332029 10.1111/hex.12044 PMC5060780

[104] See L‐C , Chang Y‐H , Chuang K‐L , et al. Animation program used to encourage patients or family members to take an active role for eliminating wrong‐site, wrong‐person, wrong‐procedure surgeries: preliminary evaluation. Int J Surg. 2011;9(3):241‐247. 21167326 10.1016/j.ijsu.2010.11.018

[105] Organization WH . Patient safety: Patients for patient safety. 2011; http://www.who.int/patientsafety/patients_for_patient/en/index.html

[106] Bergal LM , Schwarzkopf R , Walsh M , Tejwani NC . Patient participation in surgical site marking: can this be an additional tool to help avoid wrong‐site surgery? J Patient Saf. 2010;6(4):221‐225. 21500609

[107] Vincent C . Patient Involvement in Patient Safety Patient Safety. Oxford, UK: Wiley‐Blackwell; 2010:290‐306.

