# Supplementary material for: Scoping review of patients’ attitudes about their role and behaviours to ensure safe care at the direct care level
Source: Health Expect. 2020 Aug 5;23(5):979–91. doi: 10.1111/hex.13117 (PMC7696111; doi:10.1111/hex.13117)
Supplement: Supplementary file 1 — Appendix S1 [file HEX-23-979-s001.docx]

**Appendix A**

Organizations Reviewed for Relevant Grey Literature

| **#** | **Source Name** |
| --- | --- |
|  | Southeastern Ontario Local Health Integration Network (LHIN) (Canada) |
|  | Ontario Hospital Association (Canada) |
|  | Ontario Ministry of Health and Long-term Care (Canada) |
|  | Health Quality Ontario (HQO) (Canada) |
|  | The Change Foundation (Ontario, Canada) |
|  | Manitoba Institute for Patient Safety (MIPS) (Canada) |
|  | British Columbia Patient Safety & Quality Council (Canada) |
|  | Government of BC - Health - HealthLinkBC (Canada) |
|  | Health Quality Council of Alberta (HQCA) (Canada) |
|  | Alberta Health Services (Canada) |
|  | Saskatchewan Health Quality Council (HQC) (Canada) |
|  | Quebec Health and Welfare Commissioner (Canada) |
|  | New Brunswick Health Council (Canada) |
|  | Patients Canada (Canada)  *(formerly the Patients’ Association of Canada)* |
|  | Canadian Association for Person-Centred Health |
|  | Accreditation Canada (Canada) |
|  | Health Council of Canada *– website exists but council no longer operating.* (Canada) |
|  | HealthCare*CAN – formed from the merger of the Association of Canadian Academic Healthcare Organizations & the Canadian Healthcare Association in 2014.* (Canada) |
|  | Canadian Foundation for Healthcare Improvement (CFHI) *(previously Canadian Health Services Research Foundation).* (A not-for-profit organization funded by Health Canada) (Canada) |
|  | Health Canada (Canada) |
|  | Canadian Institutes of Health Research (CIHR) (Canada) |
|  | Canadian Patient Safety Institute (CPSI)  *including* Patients for Patient Safety Canada (PPSC) (Canada) |
|  | Canadian Institute for Health Information (CIHI) (Canada) |
|  | Institute for Safe Medication Practices Canada (& main site in United States (US)) |
|  | World Health Organization (WHO). Note: the WHO established the World Alliance for Patient Safety which was renamed WHO Patient Safety in 2009. |
|  | World Health Organization - Europe |
|  | International Society for Quality in Health Care (ISQua) (based in Dublin) |
|  | International Alliance of Patients’ Organizations (IAPO) |
|  | Partnership for Patient Safety (p4ps) (US) |
|  | National Patient Safety Foundation (NPSF) (US) – including the Lucian Leape Institute (LLI) |
|  | Agency for Healthcare Research & Quality (AHRQ); & AHRQ Patient Safety Network (US) |
|  | The Joint Commission (US) |
|  | Institute for Healthcare Improvement (IHI) (US) |
|  | American Institutes for Research (US) |
|  | Consumers Advancing Patient Safety (US) |
|  | The Commonwealth Fund (US) |
|  | The Beryl Institute (US) |
|  | Institute for PFCC (US) |
|  | Institute of Medicine (US) |
|  | The Australian Commission on Safety & Quality in Health Care |
|  | National Institute for Health Research (UK) |
|  | National Health Service (NHS) England  *[& includes reference to previous National Patient Safety Agency (NPSA)]* |
|  | The Health Foundation (UK) |
|  | Learning from International Networks about Errors and Understanding Safety in Primary Care (LINNEAUS EURO-PC) |
