# Supplementary material for: Scoping review of patients’ attitudes about their role and behaviours to ensure safe care at the direct care level
Source: Health Expect. 2020 Aug 5;23(5):979–91. doi: 10.1111/hex.13117 (PMC7696111; doi:10.1111/hex.13117)
Supplement: Supplementary file 2 — Appendix S2 [file HEX-23-979-s002.docx]

**APPENDIX B: Patient Attitudes** (n=35). Organized by publication year.

| **Author(s) & Year** | **Study Objective(s)** | **Design** | **Sample** | **Key Findings of Relevance** |
| --- | --- | --- | --- | --- |
| **Relevant Systematic Reviews** | | | | |
| ^1^McVeety, Keeping-Burke, Harrison, Godfrey, & Ross-White  (2014)  (Canada) | Analyze & synthesize evidence on perspectives of patients/family members who encountered adverse event in healthcare. | Systematic Review | 14 qualitative studies included in final review. | Patient/family member perspectives of experiencing an adverse event is complex and varied. Patients/family appreciated apologies from providers. Emotional and psychological consequences were typical and could be long-lasting. Patients express fear of reprisal/interference with care if they speak up about an adverse event. Patients’ feeling that a problem was unfixable and that they had no control over it, leading to no action or giving up. Patients/families act in ways to protect themselves – safeguarding. Patients described needing to advocate and intervene in certain circumstances. |
| ^2^*Doherty & Stavropoulou (2012)  (UK) | Review evidence of factors that enable and dissuade patients from being willing and able to participate in error prevention. | Systematic Review | 68 studies were included overall. They included studies involving investigation of their participation or patients’ perceptions of being involved. | Data in regard to age, gender, and education were inconclusive as consistent predictors regarding patients’ willingness or ability to engage. Cause of inability to participate may be related to illness. Generally, those perceiving a risk were more likely to engage. Self-efficacy shown to increase willingness to act. If believed patients’ role was to be passive, they were unwilling to engage. Patients less willing and able to engage if poor relationship with clinicians. Perception of staff work pressure seen as barrier to engagement. They summarise the main factors for engaging patients into 4 categories: illness; individual cognitive characteristics; the clinician-patient relationship; & organizational factors. |
| ^3^*Schwappach (2010)  (Switzerland) | Review evidence of patients’ attitudes toward engagement in safety & the effectiveness of strategies to engage patients. | Systematic Review | 21 studies were included overall. 13 publications of 11 unique studies were retained for inclusion about patients’ attitudes. | General positive attitude toward engagement, but intentions and actual behaviours vary. Theory of Planned Behaviour used as conceptual framework. (Note: one article in review is noted as “anonymous”.). |
| **Author(s) & Year** | **Study Objective(s)** | **Design** | **Sample** | **Key Findings of Relevance** |
| **Additional Publications of Relevance** | | | | |
| ^4^Chegini, Janati, Bababie, & Pouraghaei  (2019)  (Iran) | A protocol about a study regarding patient and health care provider perspectives about the role of patients in ensuring safe care in hospital. | Study Protocol for qualitative exploratory design | Patients and healthcare providers (convenience & stratified purposive sampling). Anticipate 25-30 from each group. | Not applicable. |
| ^5^Schenk, Bryant, Van Son, & Odom-Maryon  (2019)  (USA) | To explore the perceptions & attitudes of patients, family, nurses, physicians, pharmacist, & physical therapists about patient engagement in reducing preventable harm & safety risks in the hospital. | Exploratory qualitative study | 8 focus groups at 2 non-profit hospitals: including patients and families (n=14), registered nurses (n=9), physicians (n=6), pharmacists & physical therapists (n=8) | Generally, all participants agreed that engaging patients in harm prevention is important. Patients/families, although supportive of engagement, were unsure of how this should be actioned. They found that staff attitudes and availability influenced their engagement. “The thematic ideas characterizing the findings are as follows: (1) when families are around, it is safer, (2) the hospital environment is intimidating, and (3) communication is essential, but I am not being heard.” (p. 75). |
| ^6^An, Kim, Park, Moon, & Park (2017)  (Korea) | To evaluate the effects of safety education on patient perceptions and attitudes toward engagement in safety. | Quasi-experimental study (using survey) | Hospitalized military personnel; 308 participants (patients who did not receive education) & 285 participants (patients who received education) were in the study; 483 surveys completed, 252 of the respondents had safety education. | Scores for perception and attitude were greater in the intervention group. There were increased scores in perceptions for patients with higher clinical severity. The attitude scores were higher in patients with lower clinical severity conditions. They suggest that “education might have improved perceptions, but not enough to motivate speciﬁc actions, because a change in attitude requires more powerful motivation” (p. 395). |
| **Author(s) & Year** | **Study Objective(s)** | **Design** | **Sample** | **Key Findings of Relevance** |
| ^7^Bittner, Routh, Folchert, Woessner, Kennedy, & Parks  (2017)  (USA) | To determine patients and their families about attitudes toward reminding health care workers to disinfect their hands (willingness, feeling comfortable, & feeling responsible) & the implications of patient cognitive impairment. | Cross-sectional study  (Note: waiver of informed consent granted) | 143 inpatients enrolled (medical-surgical units & an ICU); 25 family responses | “Only 94 (65.7%) patients were willing and able to complete the St. Louis University Mental Status Examination; 20 had normal mental status. Of those 20 patients, 9 indicated that they were willing to give reminders, were comfortable giving reminders, and felt it their responsibility to do so.” “For a patient to remind staff to perform hand hygiene, a patient must have adequate cognitive capability and an attitude consistent with an inclination to give reminders. Such patients comprised only 6.3% of our population. Taking into account both patient and family suitability to provide reminders, reminders could be expected in only 11.2% of encounters.” (p. 81) |
| ^8^Burrows Walters & Duthie  (2017)  (USA) | To explore patients’ perceptions regarding their engagement in their care as a patient safety strategy. | Qualitative, descriptive approach using grounded theory | 13 hospitalized surgical patients with cancer; semi-structured interviews | Three themes found:   - the word “patient” obscures the message – there is a disconnect between the phrase used in health care “patient safety” and that which emerged as meaningful to participants (i.e. safety); - safety is a shared responsibility among the patient, hospital administrator, and HCP; & - involvement in safety is a right – HCP openness to communication about safety & HCP’s flexibility regarding patients changing level of engagement is required for involvement.   “Patients may be willing to accept some responsibility for ensuring their safety by engaging in behaviors that are intuitive or that they are clearly instructed to do; however, they described their involvement in their safety as a right, not an obligation.” (p. 712) |
| ^9^*Ringdal, Chaboyer, Ulin, Bucknall, & Ocelmark  (2017)  (Sweden) | To explore hospitalised patients’ preferences about participation in their care & safety activities. | Exploratory qualitative study | 20 participants who were admitted to one of four medical wards within 2 hospitals; semi-structured interviews | “…patients wanted to be active participants in their care & safety activities by having a voice & being a part of the decision-making process, sharing information & possessing knowledge about their conditions…However, a number of barriers hampered participation, such as power imbalances, lack of patient acuity & patient uncertainty. Patients’ participation in care & patient safety activities seemed to determine whether patients were feeling safe or ignored…Promoting patient participation begins by understanding the patients’ unique preferences and needs for care, establishing a good relationship & paying attention to each patient’s ability to participate despite their illness.” (p. 1) |
| **Author(s) & Year** | **Study Objective(s)** | **Design** | **Sample** | **Key Findings of Relevance** |
| ^10^****Garfield, Jheeta, Husson, Lloyd, Taylor, Boucher, Jacklin, Bischler, Norton, Hayles, & Franklin  (2016)  (UK) | To explore hospital inpatients’ involvement with medication safety-related behaviours, facilitators and barriers to this involvement, as well as electronic prescribing impact. | Ethnographic study using observations and interviews (two hospital organizations) | Interviews with 12 patients & 6 patient carers (as well as nurses, doctors & pharmacists); observation during specific events ranged from 30 patients to 226 patients | In 4 of 247 (2%) cases, paper or electronic medication records were shown to patients. “Both healthcare professionals & patients identified that patients’ knowledge & beliefs could affect their involvement. Both groups thought that some patients were more knowledgeable & interested in their medication than others & that some were more assertive & others more passive. Some patients described having ‘blind faith’ in healthcare professionals to manage their medication & did not think that their involvement was necessary. Other patients expressed concern that they may upset healthcare professionals & that their care would be affected if they challenged healthcare professionals.” (p. 9) |
| ^11^*****Rosenberg, Rosenfeld, Williams, Silber, Schlucter, Deng, Geraghty, & Sullivan-Bolyai,  (2016)  (USA) | To explore parents’ perspectives about their involvement in safety for their hospitalized children. | Qualitative, descriptive study | 10 mothers & 2 fathers (recruited from the medical-surgical unit of a 109-bed children’s service); interviews & observations | “Four consistent themes emerged from analysis: (1) Parents identify potential risks to safety, including behavioral, communication, & environmental lapses; (2) parents describe their hospital role as an extension of their “home” role as protectors; (3) parental participation in safety practice varies by individual (exhaustion, familiarity with US health care social norms) & organizational (hospital culture) factors; & (4) parents continually consider a balance between ensuring child comfort & safety, & speaking up & risking damage to relationships with clinicians that could affect care or interactions for child and family.” (p. 320) “Parents viewed their primary role in the hospital as protectors…This responsibility is both their right & their job.” (p. 321) |
| ^12^*Tobiano, Bucknall, Marshall, Guinane, & Chaboyer  (2016)  (Australia) | To examine hospitalized patients’ perceptions of participating in nursing care, & participation barriers & facilitators. | Interpretative qualitative study (as part of a larger ethnographic study) | 20 patient participants from 4 medical wards in 2 metropolitan hospitals; semi-structured interviews | “Four categories were uncovered….First, valuing participation showed patients’ willingness to participate, viewing it as a worthwhile task. Second, exchanging intelligence was a way of participating where patients’ knowledge was built & shared with health professionals. Third, on the lookout was a type of participation where patients monitored their care, showing an attentive approach towards their own safety. Fourth, power imbalance was characterised by patients feeling their opportunities for participation were restricted.” (p. 260) |
| ^13^Britnell  (2015)  (UK) | *“Patients as Partners: Renewable Energy”* (Chapter 31). In *“In Search of the Perfect Health System”.* | Text | Not applicable. | Britnell describes his personal healthcare journey in his chapter on “Patients as Partners”. Key points he writes include: patients do not report feeling empowered contrary to what leaders of healthcare organizations believe; involving patients in system design; the benefits of patient empowerment are considerably untapped. He cites the work of Coulter & Ellins (2007) in arguing the benefits to improved safety when patients are engaged. |
| **Author(s) & Year** | **Study Objective(s)** | **Design** | **Sample** | **Key Findings of Relevance** |
| ^14^Bartlova, Tothova, Brabcova, Prokesova, & Kimmer  (2014)  (Czech Republic) | Extent to which patients are involved in decisions regarding treatment & if they are interested in ensuring safety during hospitalization. | Patient opinion surveys using structured interview technique | 514 patients who had been admitted to hospital for a minimum of 3 days | 54% of patients positively rated a chance for patients or families to discuss safety issues with care. Many participants were not aware of safety aspects of care. 27.5% did not want greater involvement in decisions about care. 73.5% stated shyness or fear did not stop them from asking nursing staff questions. 18.7% acknowledged feelings of apprehension related to challenging a medical decision or behaviour. |
| ^15^Leuthold  (2014)  (Switzerland) | Patients as partners in safety. | Opinion paper | Not applicable. | The author notes many patients are confident that they can actively participate in helping to prevent adverse events (p.20). She notes that patients are usually the only ones who are always present during the course of treatment and care, placing them in a unique positon. They need to learn what kinds of observations are important. She highlights research that indicates believing and acceptance of participation does not always translate to action. |
| ^16^National Patient Safety Foundation’s Lucian Leape Institute  (2014)  (USA) | “*Safety is Personal: Partnering with Patients and Families for the Safest Care”.* | Opinion/  white paper | Not applicable. | Two roundtables with over 40 individuals from various patient safety organizations and advocacy groups. The document includes quotes and stories from participants, current initiatives in patient safety and patient engagement, and a checklist to help engage patients (with more focus on organizational/policy level than direct care specifics). |
| ^17^Martin, Navne, & Lipczak  (2013)  (Denmark) | To study practices for patient involvement in patient safety. | Ethnographic study | Observations from 4 hospital units and interviews with 25 patients, 11 doctors, 10 nurses, 4 general practitioners, and 2 gynecologists in private practice. | Patient safety not identified as topic for patients. Patients associated patient safety with the notion of “being in good hands” (p. 839). Patients did not necessarily identify actions as safety-related actions. Patients expressed willingness to engage but difficulty was identified as to how this was operationalized. Many saw patients’ role and responsibility - depending on capability and health - to get well, but noted safety as healthcare provider responsibility. Barriers included knowledge deficit related to safety, and concern of negative impact on clinician relationship. |
| **Author(s) & Year** | **Study Objective(s)** | **Design** | **Sample** | **Key Findings of Relevance** |
| ^18^**Pinto, Vincent, Darzi, & Davis (2013)  (UK) | Patient attitudes toward ‘Participate, Inform, Notice, Know (PINK)’ video on patient involvement in safety. | Qualitative semi-structure interviews | 36 inpatients | Video viewed favourably: raises awareness about involvement. However, some patients did not perceive the video as an effective error-prevention strategy primarily due to the fact that they believed errors were made based on factors beyond their control. Divided opinion on activities such as asking providers about handwashing. Most indicated they would notify a provider if something was wrong with their care even before the video. Some patients worried it would shift responsibility to the patient. |
| ^19^Walters  (2013)  (USA) | Explore perceptions of hospitalized oncology patients about their involvement in their care as a patient safety strategy. | Qualitative study – semi-structured interviews (grounded theory methods used to analyze) | 13 hospitalized patients | 3 themes: safety is a shared responsibility; the word ‘patient’ confuses the message (‘safety’ more easily described); & involvement in safety is “a right” (not obligation). Barriers to involvement: participants’ illness; concerns about being a burden to providers. Participants expressed positive attitudes toward engaging in safety but intention to act depended on nature of behaviour, healthcare providers’ acceptance, their knowledge of their condition and behaviour, and visual reminder cues in environment. Health literacy did not seem to account for differences in perspectives regarding involvement. |
| ^20^Bishop  (2012)  (Canada) | Determine the relationship between perceptions of patient safety and likelihood of patient participation in safety practices (factual & challenging actions) | Mixed methods (quantitative survey methods and qualitative focus group methods) | 217 patient & 113 provider survey responses; 10 patients participating in focus groups (2) & 27 nurses participating in focus groups (4) | Patients more willing to engage in factual safety actions such as asking questions of their provider, as opposed to challenging actions such as asking a provider about their handwashing. Perceptions of benefits versus barriers, threat, and self-efficacy were found to be determinants of patient involvement (factual and challenging actions). Patients expressed the need to have “safeguards” related to their care such as having an advocate present or taking notes. Patients expressed that they themselves can help protect themselves. |
| ^21^Davis, Anderson, Vincent, Miles, & Sevdalis  (2012)  (UK) | Examine predictors of patients’ intention to engage in: reminding staff to wash their hands, & notifying staff if they are not wearing hospital ID. | Cross-sectional survey | 80 medical and surgical patients | Patients displayed lower intention to ask staff about handwashing than notifying providers of a lack of hospital ID. Asking about handwashing was viewed as a less beneficial safety measure. Twenty of the 80 patients (25%) had prior experience of error (10 patients were unsure). Patients with experience of error had greater intention to ask about staff handwashing. |
| **Author(s) & Year** | **Study Objective(s)** | **Design** | **Sample** | **Key Findings of Relevance** |
| ^22^Coulter  (2011)  (UK) | *“Ensuring Safer Care”* (Chapter 6). In *“Engaging Patients in Healthcare”.* | Text | Not applicable. | In this chapter, A. Coulter considers ways of ensuring safer care, including the patients’ contribution. While commenting that there have been few quality studies about the impact related to their involvement, she does cite advocacy work done by patients/carer groups, and notes patients’ perspectives/concerns in a progress report done for the Department of Health in England (Carruthers and Phillip, 2006), the authors of which argue that the most successful organization in improving safety involve patients. Building on her earlier work (Coulter & Ellins, 2006) she examines in depth ways that patients can be involved: “choosing a safe healthcare provider; helping to reach an accurate diagnosis; participating in treatment decision-making; contributing to safe medication use; participating in infection control initiatives; checking the accuracy of medical records; observing and checking care process; identifying and reporting treatment complications and adverse events; practising effective self-care and monitoring treatments; and providing feedback and advocacy to focus attention on safety issues” (p. 110-111). |
| ^23^Davis, Sevdalis, & Vincent  (2011)  (UK) | Patients’ perceived willingness to participate in safety-related behaviours, and impact of providers’ encouragement on willingness. | Cross-sectional exploratory study using survey | 80 medical and surgical patients (note: patients who were healthcare professionals were excluded) | Willingness to participate was affected by the action required. For some action, whether the patient was interacting with a nurse versus a doctor made a difference related to engagement. Patients less willing to engage in challenging behaviours. Provider encouragement to ask challenging questions was suggestive of increasing patients’ willingness. No other consistent findings. |
| ^24^+Hovey, Dvorak, Burton, Worsham, Padilla, Hatlie, & Morck  (2011)  (USA) | To reconceptualise patient-centred healthcare practice for enhanced patient safety. | Qualitative (“guided by philosophical hermeneutics” (p. 664) | 21 participants who were attending the Chicago Patient Safety Workshop | One participant spoke of how important it is to feel empowered as a patient, and that the knowledge they have is important and valued. Another talked about the need to ensure providers do not feel like patients are trying to take something away from them. Another spoke of guilt and frustration of not being able to protect her child. |
| ^25^Patient Destiny in collaboration with Toronto Central LHIN  (2011)  (Canada) | *Meeting with Patients: Their Experiences and Perspectives.* | Discussion paper with quotes from 6 patients |  | Six participants agreed to participate for purposes of the report, but 40 were part of the half day workshop. Report summarizes the findings of a Dec 7, 2011, meeting with patients in collaboration with the Toronto, Canada, Central Local Health Integrated Network (LHIN). Patient narratives included: “*He* [doctor] *was very ‘put out’ that I wanted to see someone else” (p. 5). “Being involved and engaged has worked well for me*” (p. 6). *“Providers need to listen to patient – we know ourselves best”* (p. 6). |
| **Author(s) & Year** | **Study Objective(s)** | **Design** | **Sample** | **Key Findings of Relevance** |
| ^26^Rathert, Brandt, & Williams  (2011a)  (USA) | Consumer perceptions of patient safety. | Qualitative study (using interpretive analytical approach) | 39 with recent overnight hospital stay participate in 1 of 4 focus group interviews | Delays or lack of information was aligned with safety as opposed to quality. Agreement that patients need family as advocates, and generally believed that if family is present, they may be able to prevent a problem. To feel safe, participants wanted open, timely and accurate communication. Participants asked question about medications. |
| ^27^Rathert, Huddleston, & Pak  (2011b)  (USA) | Patient beliefs about their role in safety. | “Qualitative study” (p.134) using mailing methodology of patients who had overnight stay in past 90 days | Of 1,040 respondents, 491 gave response to open-ended question regarding patient role in safety. | Most prominent of responses noted that patient’s role is to follow instructions of care providers. Also, patients should ask questions and know about their condition and treatment. Second most frequent comment category – patients should expect competent care. |
| ^28^***Schwappach, Frank, Koppenberg, Muller, & Wasserfallen (2011)  (Switzerland) | Attitudes toward and experience with safety advisory. | Cross-sectional survey  (All patients admitted to study units received booklet that encourages patient in safety through vigilance, communication & cooperation; Takes 15 minutes to read). (Survey given at discharge) | 1053 patients; 275 healthcare workers at three hospitals | 75% of patients responding reported to have read the entire booklet.  95% of patients agreed that hospital should educate patients how to prevent errors. The survey is based on the Theory of Planned Behaviour, focusing on their intention to engage (& not whether they actually did). Overall, patients had a high level of perceived behavioral control & had strong intentions to apply the advice. More than one quarter did notify staff of a potential error. Of 5 recommended actions, the mean scores for acceptance and actual adoption were 6.0, and 4.3 respectively (p<0.001) (actual adoption was highest in asking staff the purpose of a medication & inform staff the medication they usually took). However, 15% of patients strongly disagreed that providers would expect them to apply the recommendations. Fear of negative staff reactions was reported as the main barrier to applying the advised actions. (Notably, the advisory was developed with input from patient/relatives focus groups.). |
| ^29^Cumbler, Wald, & Kutner  (2010)  (USA) | Assess hospitalized patients knowledge of their hospital medications & attitudes toward involvement in medication safety while hospitalized. | Cross-sectional survey | 50 adult internal medicine patients (completed list of their current prescribed medications & survey of attitude toward involvement) | 90% wanted to review their medication list for accuracy and 94% of participants felt patient participation and review of hospital medications had the potential to reduce errors. |
| **Author(s) & Year** | **Study Objective(s)** | **Design** | **Sample** | **Key Findings of Relevance** |
| ^30^+Hovey, Morck, Nettleton, Robin, Bullis, Findlay, & Massfeller  (2010)  (USA & Canada) | The of the patient in patient safety is explored by the Calgary Health Region’s Patient and Family Safety Council perspective and the Calgary Health Region. | Interpretative phenomenology approach | Patient and Family Safety Council members involved (n=4 who acted as co-researchers) & 11 involved in reading & providing thoughts on two articles about involvement. | Patient comments include that patient involvement is meant to complement not replace healthcare provider care. Another individual notes it is really about partnership. Another person believes if patient safety does not begin with and include the patient, an important piece is lost. |
| ^31^Schwappach & Wernli  (2010b)  (Switzerland) | Examined patients’ attitudes, norms, behavioural control, and intentions to engage in error prevention. | Quantitative, cross-sectional survey | 479 chemotherapy patients r/t drug administration safety. | Patients see the benefit of error monitoring and reporting, though some did reject intention to engage in safety. They anticipate positive outcomes related to involvement. Trust in one’s ability was the major predictor of intention to monitor safety. Their beliefs about the process of engaging in safety was not viewed as favourably. The authors note that if patients believe interaction with staff is needed for error prevention but also believe interaction opportunity is limited, intention to engage decreases. |
| ^32^Vincent  (2010)  (UK) | *“Patient Involvement in Patient Safety”* (Chapter 15)*.* In *“Patient Safety”.* | Text |  | In his book about Patient Safety, C. Vincent addresses the issue of patient engagement in safety. He provides evidence from studies wherein authors examined patients’ beliefs about what they thought they would do or were prepared to do, with responses varying based on the action required. He identifies evidence of patients’ ability to identify error. He comments on their role at the bedside and beyond, summarizing that it is a complex issue discerning the right role for patients in patient safety yet positive results have been seen. |
| ^33^Meyers  (2008)  (USA) | Reflections on Patient and Family Advisors related to quality and safety. | Discussion paper | Select patient narratives and provider opinions. | The author provides examples of hospitals where patient- and family centred care is a philosophy, and where patient advisors are integrated into the setting. Family member story of care is shared. The patient ‘voice’ is advocated at a direct care to a system level to enhance safety & quality. |
| **Author(s) & Year** | **Study Objective(s)** | **Design** | **Sample** | **Key Findings of Relevance** |
| ^34^Spath  (2008)  (USA) | *“Safety from the Patient’s Point of View”* (Chapter 1). In *“Engaging Patients as Safety Partners”.* | Text | Not applicable. | In her book, P. Spath, together with other authors, discuss the issue of patient safety from the patient’s perspective and the patient’s role in safety, as well as opportunities and barriers to this engagement. She suggests that “most agree that creating safety partnerships with consumers is the right thing to do” (p. xxviii). Notably, she provides some evidence that patients can recognize error & so their involvement is of value, but acknowledges that it is important to consider the opinions/preferences of patients/families. |
| ^35^*Davis, Jacklin, Sevdalis, & Vincent  (2007)  (UK) | Factors affecting the participation of the patient in safety. | Literature review | Selectively reviewed evidence on both direct & indirect factors (limited detail on search process). | Five categories emerged: Patient-related (knowledge & beliefs; demographic characteristics; emotional experiences & coping styles); Illness-related (stage & severity of illness; illness symptoms, treatment plan and patients’ health outcomes; other illness-related factors: prior experience of illness and/or prior experience of patient safety incidents); Health care professional-related (knowledge & beliefs; interactions with patients; health care professionals’ professional role); Health care setting-related (i.e. primary, secondary, or tertiary); Task-related factors (e.g. confronting healthcare provider versus keeping record of medical history). |
| ^36^Elder, Regan, Pallerla, Levin, Post, & Cegela (2007)  (USA) | Development & testing of a survey instrument to measure seniors’ views about patient safety tasks. | Pilot test | 143 community dwelling seniors. | A 21-item tool is presented. Example of statements/questions include: *I feel comfortable changing doctors if I think my health concerns are not being met; How strongly do you believe that teaching yourself about your own health problems and medicines will improve your overall health?; How confident are you that you could call the doctor’s office if you haven’t receive the results of laboratory or X-ray tests? How often do you ask your doctors questions about your health problems, lab tests and medicines?* |
| ^37^Goeltz & Hatlie  (2004)  (USA) | “*Trial and Error in My Quest to Be a Partner in My Health Care: A Patient’s Story”* (Chapter 14)*.* In the *“The Patient Safety Handbook”.* | Text | Not applicable. | In this chapter, R. Goeltz’s personal narrative is shared, with commentary from M. Hatlie. R. Goeltz’s story of her health care experiences are poignant, and in reflecting on one experience, she recounts how she had to take the initiative in order to enable a system process – she ponders the potential time lost had she not done this, and its possible negative implication. She emphasizes the lessons she has learned, particularly related to a family member’s death, and the importance of active participation in the care of her health. |

+This study is detailed for its significance but is not counted in the final set as it is captured in a review.

*This study is included in Patient Behaviours – General table given items of relevance.

**This study is included in Patient Behaviours – Speaking Up table given items of relevance.

***This study is included in Patient Behaviours – Health Literacy/Safety Tips given items of relevance.

****This study is included in Patient Behaviours – Medication Safety given items of relevance.

*****This study is included in Patient Behaviours – Advocacy-Parents given items of relevance.

References

1. McVeety J, Keeping-Burke L, Harrison MB, Godfrey C, Ross-White A. Patient and family member perspectives of encountering adverse events in health care: a systematic review. *JBI Database of Systematic Reviews and Implementation Reports.* 2014;12(7):315-373.

2. Doherty C, Stavropoulou C. Patients' willingness and ability to participate actively in the reduction of clinical errors: a systematic literature review. *Social Science & Medicine.* 2012;75(2):257-263.

3. Schwappach DLB. Review: engaging patients as vigilant partners in safety: a systematic review. *Medical care research and review : MCRR.* 2010;67(2):119-148.

4. Chegini Z, Janati A, Bababie J, Pouraghaei M. The role of patients in the delivery of safe care in hospital: Study protocol. *J Adv Nurs.* 2019;75(9):2015-2023.

5. Schenk EC, Bryant RA, Van Son CR, Odom-Maryon T. Developing an Intervention to Reduce Harm in Hospitalized Patients: Patients and Families in Research. *Journal of Nursing Care Quality.* 2019;34(3):273-278.

6. An J, Kim SJ, Park S, Moon KT, Park EC. The effects of patient education on patient safety: can we change patient perceptions and attitudes?: Lessons from the Armed Forces Capital Hospital in Korea. *Int J Qual Health Care.* 2017;29(3):392-398.

7. Bittner MJ, Routh JM, Folchert MD, Woessner NE, Kennedy SJ, Parks CC. Hand Hygiene Among Health Care Workers: Is Educating Patients and Families a Feasible Way to Increase Rates? *WMJ.* 2017;116(2):79-83.

8. Burrows Walters C, Duthie EA. Patients' Perspectives of Engagement as a Safety Strategy. *Oncology Nursing Forum.* 2017;44(6):712-718.

9. Ringdal M, Chaboyer W, Ulin K, Bucknall T, Oxelmark L. Patient preferences for participation in patient care and safety activities in hospitals. *BMC Nursing.* 2017;16:1-8.

10. Garfield S, Jheeta S, Husson F, et al. The Role of Hospital Inpatients in Supporting Medication Safety: A Qualitative Study. *PloS one.* 2016;11(4):e0153721.

11. Rosenberg RE, Rosenfeld P, Williams E, et al. Parents' Perspectives on "Keeping Their Children Safe" in the Hospital. *Journal of nursing care quality.* 2016;31(4):318-326.

12. Tobiano G, Bucknall T, Marshall A, Guinane J, Chaboyer W. Patients' perceptions of participation in nursing care on medical wards. *Scandinavian Journal of Caring Sciences.* 2016;30(2):260-270.

13. Britnell M. *In search of the perfect health system.* Palgrave Macmillan; 2015.

14. Bartlova S, Tothova V, Brabcova I, Prokesova R, Kimmer D. The hospitalized patient as a partner in the survey on safe care in the Czech Republic. *Neuroendocrinology Letters.* 2014;35 Suppl 1:5-10.

15. Leuthold M. Patients as partners for improving safety. *World Hospitals & Health Services.* 2014;50(3):20-22.

16. National Patient Safety Foundation’s Lucian Leape Institute. Safety is Personal: Partnering with Patients and Families for the Safest Care. Boston, MA; 2014.

17. Martin HM, Navne LE, Lipczak H. Involvement of patients with cancer in patient safety: a qualitative study of current practices, potentials and barriers. *BMJ Quality & Safety.* 2013;22(10):836-842.

18. Pinto A, Vincent C, Darzi A, Davis R. A qualitative exploration of patients' attitudes towards the 'Participate Inform Notice Know' (PINK) patient safety video. *International Journal for Quality in Health Care.* 2013;25(1):29-34.

19. Walters CB. Perceptions of hospitalized oncology patients regarding involvement in their care as a patient safety strategy across a range of health literacy levels. 2013.

20. Bishop AC. *Perceptions of Patient Safety: What Influences Patient and Provider Involvement?* Halifax, Nova Scotia, Dalhousie University; 2012.

21. Davis R, Anderson O, Vincent C, Miles K, Sevdalis N. Predictors of hospitalized patients' intentions to prevent healthcare harm: a cross sectional survey. *International journal of nursing studies.* 2012;49(4):407-415.

22. Coulter A. *Engaging patients in healthcare.* McGraw-Hill Education (UK); 2011.

23. Davis RE, Sevdalis N, Vincent CA. Patient involvement in patient safety: How willing are patients to participate? *BMJ quality & safety.* 2011;20(1):108-114.

24. Hovey RB, Dvorak ML, Burton T, et al. Patient safety: a consumer's perspective. *Qualitative health research.* 2011;21(5):662-672.

25. Toronto PDicw, LHIN C. Meeting with patients: Their experiences and perspectives. In. Toronto, ON: Patient Destiny in collaboration with Toronto Central LHIN; 2011.

26. Rathert C, Brandt J, Williams ES. Putting the 'patient' in patient safety: a qualitative study of consumer experiences. *Health expectations : an international journal of public participation in health care and health policy.* 2011;15(3):327-336.

27. Rathert C, Huddleston N, Pak Y. Acute care patients discuss the patient role in patient safety. *Health care management review.* 2011;36(2):134-144.

28. Schwappach DLB, Frank O, Koppenberg J, Muller B, Wasserfallen J-B. Patients' and healthcare workers' perceptions of a patient safety advisory. *International journal for quality in health care : journal of the International Society for Quality in Health Care.* 2011;23(6):713-720.

29. Cumbler E, Wald H, Kutner J. Lack of patient knowledge regarding hospital medications. *Journal of hospital medicine.* 2010;5(2):83-86.

30. Hovey RB, Morck A, Nettleton S, et al. Partners in our care: patient safety from a patient perspective. *Quality & safety in health care.* 2010;19(6):e59.

31. Schwappach DLB, Wernli M. Predictors of chemotherapy patients' intentions to engage in medical error prevention. *The oncologist.* 2010;15(8):903-912.

32. Vincent C. Patient involvement in patient safety (pp. 290-306). In: *Patient Safety.* Oxford, UK: Wiley-Blackwell; 2010.

33. Meyers S. Take heed. How patient and family advisors can improve quality. *Trustee : the journal for hospital governing boards.* 2008;61(4):14-11.

34. Spath PL. Safety from the patient’s point of view. In: Spath PL, ed. *Engaging patients as safety partners: a guide for reducing errors and improving satisfaction.* Chicago, IL: Health Forum Incorporated; 2008:1-40.

35. Davis RE, Jacklin R, Sevdalis N, Vincent CA. Patient involvement in patient safety: what factors influence patient participation and engagement? *Health expectations : an international journal of public participation in health care and health policy.* 2007;10(3):259-267.

36. Elder NC, Regan SL, Pallerla H, Levin L, Post D, Cegela DJ. Development of an instrument to measure seniors' patient safety health beliefs: the Seniors Empowerment and Advocacy in Patient Safety (SEAPS) survey. *Patient Educ Couns.* 2007;69(1-3):100-107.

37. Goeltz R, Hatlie MJ. Trial and error in my quest to be a partner in my health care: a patient's story. In: Hatlie BJYMJ, ed. *The Patient Safety Handbook.* Vol 14. Sudbury, MA: Jones and Bartlett Publishers; 2004:pp. 225-240.
