# Supplementary material for: Scoping review of patients’ attitudes about their role and behaviours to ensure safe care at the direct care level
Source: Health Expect. 2020 Aug 5;23(5):979–91. doi: 10.1111/hex.13117 (PMC7696111; doi:10.1111/hex.13117)
Supplement: Supplementary file 3 — Appendix S3 [file HEX-23-979-s003.docx]

**APPENDIX C**

Table A

***Patient Behaviours*** *– General (n=26)*

| **Author(s) & Year** | **Study Objective(s)** | **Design** | **Sample** | **Relevant Findings** |
| --- | --- | --- | --- | --- |
| **Systematic Reviews & Literature Reviews** | | | | |
| ^1^Berger, Flickinger, Pfoh, Martinez, & Dy  (2014)  (USA) | “This systematic review focuses on the definition and implementation of patient engagement as part of selected hospital-based Patient Safety Practices (PSPs) with the primary intent of increasing patient/family involvement to improve patient safety” (p. 549). | Systematic Review | 6 articles of effectiveness on patient engagement; 12 articles on implementing patient engagement as part of selected broader PSPs. | Authors suggest evidence is lacking related to the types of patients who feel comfortable engaging and in what context. |
| ^2^Vaismoradi, Jordan, & Kangasniemi (2014/2015)  (Norway) | Synthesize research on how patients participate in patient safety initiatives. | Systematic Review | 17 articles (4 qualitative; one mixed method; 12 quantitative). | All evidence found suggests patient can participate in safety initiatives. Strategies to engage patients should accommodate patients’ abilities and health beliefs, personal illness coping and past experiences in healthcare. |
| ^3^*Doherty & Stavropoulou (2012)  (UK) | Review evidence of factors that enable and dissuade patients from being willing and able to participate in error prevention. | Systematic Review | 68 studies were included overall. They included studies involving investigation of their participation or patients’ perceptions of being involved. | Data in regard to age, gender, and education were inconclusive as consistent predictors regarding patients’ willingness or ability to engage. Cause of inability to participate may be related to illness. Generally, those perceiving a risk were more likely to engage. Self-efficacy shown to increase willingness to act. If believed patients’ role was to be passive, they were unwilling to engage. Patients less willing and able to engage if poor relationship with clinicians. Perception of staff work pressure seen as barrier to engagement. They summarise the main factors for engaging patients into 4 categories: illness; individual cognitive characteristics; the clinician-patient relationship; & organizational factors. |
| **Author(s) & Year** | **Study Objective(s)** | **Design** | **Sample** | **Relevant Findings** |
| ^4^Hall, Peat, Birks, Golder, on behalf of the PIPS Group: Entwistle, Gilbody, Mansell, McCaughan, Sheldon, Watt, Williams, & Wright  (2010)  (UK) | Review of effectiveness of interventions that promote patient involvement in patient safety in healthcare. | Systematic Review | 14 individual experimental and quasi- experimental plus one systematic review (related to long-term oral anticoagulation therapy). | All studies were related to enhancing medication safety. Not possible to draw decisive conclusions, except related to self-management of anticoagulation (favoured interventions that involved patients). The authors acknowledge that the review did not sufficiently address all potential areas of patient involvement. |
| ^5^*Schwappach (2010)  (Switzerland) | Review evidence of patients’ attitudes toward engagement in safety & the effectiveness of strategies to engage patients. | Systematic Review | 21 studies were included overall. 13 publications of 11 unique studies were retained for inclusion about patients’ attitudes. | General positive attitude toward engagement, but intentions and actual behaviours vary. Theory of Planned Behaviour used as conceptual framework. (Note: one article in review is noted as “anonymous”.). |
| ^6^Scobie & Persaud (2010)  (Canada) | Barriers and facilitators to patient engagement in safety. | Literature review & commentary | Not applicable. | The authors suggest patient engagement in safety is aimed at “increasing the awareness and participation of patients in error prevention” (p. 43). Barriers to engagement include lack of awareness about safety issues; patients do not see a role for themselves; acute illness. Facilitators include patient instruction; knowledge of how to prevent error. Patients indicated willingness. |
| ^7^*Davis, Jacklin, Sevdalis, & Vincent  (2007)  (UK) | Factors affecting the participation of the patient in safety. | Literature Review | Selectively reviewed evidence on both direct and indirect factors (limited detail on search process). | Five categories emerged: Patient-related (knowledge & beliefs; demographic characteristics; emotional experiences & coping styles); Illness-related (stage & severity of illness; illness symptoms, treatment plan and patients’ health outcomes; other illness-related factors: prior experience of illness and/or prior experience of patient safety incidents); Health care professional-related (knowledge & beliefs; interactions with patients; health care professionals’ professional role); Health care setting-related (i.e. primary, secondary, or tertiary); Task-related factors (e.g. confronting healthcare provider versus keeping record of medical history). |
| **Author(s) & Year** | **Study Objective(s)** | **Design** | **Sample** | **Relevant Findings** |
| **Additional Articles of Relevance** | | | | |
| ^8^Liberatore  (2018)  (USA) | To evaluate engagement of Pennsylvania patients in common safety practices and inform future patient safety efforts. | Survey (telephone) | 606 adults in Pennsylvania | Surveys were previously conducted in 2006 & 2013. Respondents were likely to ask for further explanation, question medication, and question procedures. Willingness to engage in practices declined from 2013-2018. “Comparing the poll results from 2006 to 2018, statistically significant improvements were observed in the likelihood of patients asking about healthcare-worker handwashing and patient identification. Comparing the poll results from 2013 to 2018, statistically significant declines were observed in the likelihood of engagement in healthcare-worker handwashing and questioning procedures.” (p. 8) |
| ^9^*Ringdal, Chaboyer, Ulin, Bucknall, & Oxelmark  (2017)  (Sweden) | To explore hospitalised patients’ preferences about participation in their care & safety activities. | Exploratory qualitative study | 20 participants who were admitted to one of four medical wards within 2 hospitals; semi-structured interviews | “…patients wanted to be active participants in their care & safety activities by having a voice & being a part of the decision-making process, sharing information & possessing knowledge about their conditions…However, a number of barriers hampered participation, such as power imbalances, lack of patient acuity & patient uncertainty. Patients’ participation in care & patient safety activities seemed to determine whether patients were feeling safe or ignored…Promoting patient participation begins by understanding the patients’ unique preferences and needs for care, establishing a good relationship & paying attention to each patient’s ability to participate despite their illness.” (p. 1) |
| ^10^Thomas, Silver, Rathe, Robinson, Wald, Bell & Harel  (2016)  (Canada) | To determine the feasibility of using a hemodialysis safety checklist (*Hemo Pause* safety checklist) during every hemodialysis session for 3 months. | Single-center, prospective time series study | n=14 nurses; n=22 prevalent in-center hemodialysis volunteer patients  “A key element of the checklist is the ‘time out’ section, whereby nurses engage in a conversation with the patient and the patient has an opportunity to ask questions about their treatment plan and correct any errors or omissions.” (p. 336) | There were 799 hemodialysis treatments pre-intervention & 757 post-intervention. The checklist was completed for 556 of the 757 (73%) treatments. Among the hemodialysis nurses, 13 of 14 agreed that the checklist was easy to use & 11 of 14 agreed it should be expanded to other patients. Among the hemodialysis patients, 16 of 22 agreed that the checklist made them feel safer & should be expanded to other patients.  “Patients noted that their initial reluctance to participate in safety initiatives was not substantiated at the end of the study. Their concerns centered on how their feedback would be perceived by nurses, as well as treatment delays from the new process. At the end of the study, the patients appreciated the opportunity to work collaboratively with nurses on a shared purpose. They felt their input was valued and important, which outweighed any small disruptions to treatment duration.” (p. 340) |
| **Author(s) & Year** | **Study Objective(s)** | **Design** | **Sample** | **Relevant Findings** |
| ^11^*Tobiano, Bucknall, Marshall, Guinane, & Chaboyer  (2016)  (Australia) | To examine hospitalized patients’ perceptions of participating in nursing care, & participation barriers & facilitators. | Interpretative qualitative study (as part of a larger ethnographic study) | 20 patient participants from 4 medical wards in 2 metropolitan hospitals; semi-structured interviews | “Four categories were uncovered….First, valuing participation showed patients’ willingness to participate, viewing it as a worthwhile task. Second, exchanging intelligence was a way of participating where patients’ knowledge was built & shared with health professionals. Third, on the lookout was a type of participation where patients monitored their care, showing an attentive approach towards their own safety. Fourth, power imbalance was characterised by patients feeling their opportunities for participation were restricted.” (p. 260) |
| ^12^Spruce  (2015)  (USA) | Presents ideas for perioperative nurses to think about engaging patients in patient safety. | Opinion paper | Not applicable. | The author argues that prevention of error can be helped by involving patients in care planning and delivery. She provides a case study, where a 35 year old woman has an adverse event during a surgical procedure, and she feels guilty for not having told staff about other medications she was taking. The author provides the reader with benefits/strategies to engage with patients/families. |
| ^13^Sahlstrom, Partanen, & Turunen (2014)  (Finland) | To describe patients’ experiences of patient safety during their most recent period of care and the degree to which they viewed themselves as participating in safety. | Cross-sectional survey | 175 patients | 20% of patients had experienced errors at some time during their care. Patients who had experienced errors and those treated at inpatient units versus day surgery unit were most critical toward patient participation, specifically that they were not being involved. |
| ^14^Gillespie (2013)  (Australia) | Profile of the work of Professor Wendy Chaboyer regarding partnering with patients for safety. | Researcher Profile | Not applicable. | The author suggests there is little evidence about types of strategies to be used to promote partnering with patients for safety. She profiles the work of Professor Chaboyer who is leading a series of studies about patient participation in safety activities (3 year study). This work includes obtaining patient perceptions of barriers and enablers to participation in safety activities. The final phase is development of a framework to promote participation. |
| **Author(s) & Year** | **Study Objective(s)** | **Design** | **Sample** | **Relevant Findings** |
| ^15^Hor, Godbold, Collier, & Iedema  (2013)  (Australia) | Explore data from three studies (two ongoing & one recently complete) to show how patients & carers are contributing to their own safety. | Opinion paper | Not applicable. | The authors argue that “it is no longer sufficient to discuss *if* patients should be involved with ensuring their own safety” (p. 567). They also note that the voice of patients has largely been missing from the safety research and programs. They draw information from 3 studies (two ongoing & one recently completed as of this article publication date). From their developing data, they provide some patient quotes, specifically related to patients taking safety action already, and sometimes because safety expectations had not been met. |
| ^16^World Health Organization: Regional Office for Europe  (2013)  (Denmark) | Exploring patient participation in reducing health-care-related safety risks. | Opinion paper | Not applicable. | Extensive opinion paper (190 pages), with evidence, in considering patient participation in safety. They considered blood transfusion, hand hygiene, surgical safety, and primary care. In concluding remarks, offers that patient involvement is essential and that on a general level patients “support an active role for patients in error prevention” (p. 147). They acknowledge the preferred role may be more of a traditional one such as information sharing, rather than a challenging one. They note many patients view their role as a passive one, and that patients do not see safety as their responsibility. |
| ^17^Birks, Hall, McCaughan, Peat, & Watt (2011)  (UK) | Report of 3 phases of linked studies examining how patients might promote their own safety. | 3-phased study using mixed methods (literature review; collecting patient views: development & piloting a potential patient involvement strategy. | Patients included at different phases (e.g. 5 patients in phase three; number of those in phase two not clear, only that they were across six different groups) | In broad terms, involvement in safety was acceptable. Participants could identify with a number of roles through which they might enhance safety, but there was no consistency. They identified a range of factors that affected their willingness or ability to raise care concerns. |
| ^18^Weingart, Zhu, Chiappetta, Stuver, Schneider, Epstein, David-Kasdan, Annas, Fowler, & Weissman  (2011)  (USA) | To understand the extent to which hospitalized patients participate in their care and the association of that participation with safety and quality. | Survey (telephone) | 2025 recently hospitalized adults. | 99.9% of patients reported positively to at least one of seven measures of participation in safety behaviours. There was an inverse relationship between patient participation and adverse events. Participation was associated with favourable judgements of hospital quality. |
| **Author(s) & Year** | **Study Objective(s)** | **Design** | **Sample** | **Relevant Findings** |
| ^19^Clark  (2010)  (USA) | Emergency Department patients’ perspectives of safety. (Thesis) | Qualitative - ethnographic | 14 participants completed 1 in-person interview | Safe care for patients is involvement in their care and making decisions with providers. Safe care is protecting patient physical safety by removing obstacles, following proper hand hygiene standards, and patient ID practices. Patient examples are given whereby patients describe seeing glove use, masking, etc., suggestive that they are aware of safety practices. |
| ^20^Davis  (2009)  (UK) | Hospital patients’ participation in safety-related aspects of care. (Doctoral Thesis) | Mixed methods | 580 medical and surgical inpatients | Data collected from 580 medical and surgical inpatients using mixed method approach to determine patient preferences for taking an active role in safety. Patients hesitant to engage in behaviours that may be perceived as challenging. |
| ^21^Jorm, Dunbar, Sudano, & Travaglia (2009)  (Australia) | Make patient safety more patient centred. | Opinion paper | Not applicable. | Authors suggest emphasis on “patient-centeredness as an essential characteristic of safe and high quality care” (p. 390). They propose six areas of action to make patient safety more patient centred. They suggest patient safety programs need to be informed by patients and consumers. Use of patient narratives and experiences is encouraged. They also encourage patient empowerment and involvement of patients in care and safety, thereby becoming more patient centred. |
| ^22^**Marella, Finley, Thomas, & Clarke  (2007)  (USA) | To assess consumers’ inclination to engage in selected patient safety practices. | Survey (telephone) | 856 randomly selected adults in Pennsylvania. | Consumers willing to ask for explanations, question unexpected procedures, or unfamiliar drugs, and seek second opinions but are less inclined to ask for confirmation of their identity before a procedure or to ask a provider if they have washed their hands. |
| ^23^Entwistle (2007)  (UK) | Considers the differing perspectives about patient participation in patient safety. | Opinion paper |  | The authors emphasizes that *relying on* patients and *involving* patients are two different things, and that participation should be considered in this way. She provides a clinical example to demonstrate how providers can encourage engagement through education by explaining to patients why certain activities/procedures are done as part of helping patient understand the safety rationale and thereby gaining perspective in a new way. |
| ^24^Lyons  (2007)  (UK) | Considers the role patients should have in safety. | Opinion paper |  | The author provides a list of pros and cons to patient involvement in safety. She suggests that pros include patients know their backgrounds & as such progression of symptoms; self-interest; availability and proximity meaning they are the first “link in the chain” (p. 140). Cons include patient vulnerabilities or limitations and whether it is unreasonable to burden them with this role. |
| **Author(s) & Year** | **Study Objective(s)** | **Design** | **Sample** | **Relevant Findings** |
| ^25^Coulter  (2006)  (UK) | The role patients can have in patient safety. | Opinion paper |  | She notes the literature is sparse in this area, and in particular notes the gap of knowing to what extent patients want to take a role and in what ways. |
| ^26^+Unruh & Pratt  (2007)  (USA) | Examine patient’s role in detecting, preventing and recovering from error in outpatient oncology | Qualitative  (part of larger study) | 5 cases | Patients are engaged in activities that prevent error. They alert staff and observe care processes. They stop actions that could led to error. They are attentive to procedural information (e.g. patient described not wanting to get ‘stuck twice’). |
| ^27^Entwistle (2004)  (UK) | Explores whether clinical monitoring by families is part of the solution to nursing shortage and safety problems. | Opinion paper |  | She highlights, as a visitor in the USA while writing the paper, the frequency of messaging she sees about bringing a family member while in hospital to provide safeguarding or patient vigilance against errors. She questions the practical and ethical issues of patient involvement in safety. She cautions that research is needed to support such advances. |
| ^28^Vincent & Coulter  (2002)  (UK) | Discussing patient involvement in patient safety. | Opinion paper |  | The authors provide strategies for involving patients in promoting safety. They caution against placing the burden on patients who are seriously ill or anxious, but also suggest that when they are ill it may be even more important to engage them and understand their perspectives/opinions. |

+This study found in Doherty and Stavropoulou (2012) Systematic Review is noted for information but not repeated in final set count.

*This study is included in Patient Attitudes table given items of relevance but only counted once in final set count.

**Study found in Davis, Parand, Pinto, & Buetow (2015) Systematic Review on Handwashing so not repeated in final set count.

Table B

***Patient Behaviours*** *– Patient Reporting (n=5)*

| **Author(s) & Year** | **Study Objective(s)** | **Design** | **Sample** | **Relevant Findings** |
| --- | --- | --- | --- | --- |
| ^29^Lawton, O’Hara, Sheard, Armitage, Cocks, Buckley, Corbacho, Reynolds, Marsh, Moore, Watt, & Wright  (2017)  (UK) | To evaluate the efficacy of the **P**atient **R**eporting and **A**ction for a Safe **E**nvironment (PRASE) intervention | Multicentre cluster randomised controlled trial | 33 wards (16 control & 17 intervention) | Patients are willing to provide feedback about the safety of their care.  “Intervention uptake and retention of wards was 100% and patient participation was high (86%). We found no significant effect of the intervention on any outcomes at 6 or 12 months. However, for new harms (i.e, those for which the wards were directly accountable) intervention wards did show greater, though non-significant, improvement compared with control wards. Analyses also indicated that improvements were largest for wards that showed the greatest compliance with the intervention.” (p. 622) |
| ^30^Sheard, O’Hara, Armitage, Wright, Cocks, McEachan, Watt, & Lawton (2014)  (UK) | Assess the efficacy of the **P**atient **R**eporting and **A**ction for a **S**afe **E**nvironment (PRASE) intervention on improvement in safety over 12-month period. (Study Protocol) | Multi-centre, cluster randomised controlled trial, randomising units to intervention or control in 1:1 ratio; with a qualitative process evaluation | Will involve 32 hospital units in 3 NHS Hospital Trusts in North of England. Plan to recruit 2,400 patients. | Patient component will involve asking patients to complete the Patient Measure of Safety (PMOS) (44-item: 8 domains including communication & team working, organization & care planning, access to resources, unit type & layout, information flow, staff roles & responsibilities, equipment & delays); Patient Safety Incident Reporting Tool (PIRT) (to report any safety concerns or positive experiences of care); 4 additional questions on whether involvement in decisions about care/treatment was what they wanted; if they could find someone to talk to about concerns if they had them; if they were given enough privacy; & whether they would recommend the unit to family/friends. |
| ^31^Davis, Sevdalis, Neale, Massey, & Vincent  (2013)  (UK) | Investigate hospital patients’ reports of undesirable events in their health care. | Cross-sectional mixed methods | 80 medical & surgical patients (interviewed post-discharge over the phone using survey with 31 items [yes/no/do not know response option]; with 1 open ended question for any comments). Also, 3 questions on willingness to report errors. Medical records were also reviewed. | 258 undesirable events reported (rate of 3.2 per person). Patients identified events that were not captured in medical record (n=27 of which reviewers believed should have been recorded). Patients identified 30 of the 36 errors recorded in the medical record. Patients were more willing to report events to a researcher than a local or national reporting system. |
| **Author(s) & Year** | **Study Objective(s)** | **Design** | **Sample** | **Relevant Findings** |
| ^32^Hasegawa, Fujita, Seto, Kitazawa, & Matsumoto  (2011)  (Tokyo) | Compare patients’ and healthcare staff’s identification and reporting of unsafe events. | Survey | 1,506 outpatients;  1,738 inpatients while hospitalized (or could mail in after discharge). Included 3 university teaching hospitals, 2 community hospitals & 1 care-mix hospital. | 8.7% of outpatients & 10.9% of inpatients experienced uneasy-dissatisfying or unsafe events. Only 38 (30.4%) of outpatients and 62 (33.5%) of inpatients reported the uneasy-dissatisfying or unsafe events to staff. Reasons for not reporting included: believed they were self-evident; the potential negative impact on their treatment; & not expecting report to bring improvement. |
| ^33^Wasson, MacKenzie, & Hall  (2007)  (USA) | Investigate how an automated health assessment system can be used to identify adverse events. | Cross-sectional survey of patient perceptions of adverse events experienced during previous year. | 44,860 adults in USA who responded during a 2 year timeframe. | 1.4% of patients report possible adverse events. Eight times higher for those with greatest burden of illness. Authors conclude patients will use an internet survey to identify adverse events. |

Table C

***Patient Behaviours*** *– Healthcare-associated Infections (n=3)*

| **Author(s) & Year** | **Study Objective(s)** | **Design** | **Sample** | **Relevant Findings** |
| --- | --- | --- | --- | --- |
| ^34^Ball, George, Duval, & Hedrick (2016)  (USA) | To design a joint quality improvement/patient engagement activity to decrease bloodstream infection (BSI) rates in an effort to improve quality of care, engage patients in their own care, & reduce morbidity & mortality secondary to infection. (p. S7) | Quality improvement (included survey tool for patients that included reminders about ‘speaking up’ if staff fail to glove/masks/etc.; & questioned whether staff followed these procedures); educational puzzles also provided between surveys; a follow-up survey was also done regarding the educational component | “Dialysis facilities were ranked utilizing 2014 National Healthcare Safety Network (NHSN) data. Selection included 20% of Network 13 facilities (n=58) with the highest BSI rates, which captured 31% of the patient population.” (p. S7) | “Over the course of the six months of this QI activity, statistically significant (P < 0.001) improvement was achieved in the reduction of BSIs; increase in patient engagement in the infection control process; and, correct completion of hand hygiene audits.” (p. S10)  “A follow-up survey on the effectiveness of educational resources provided to patients was conducted at the conclusion of the activity. There were 1028 (28%) patients who responded to the survey out of the 3700 patients included in this activity.” (p. s11) Responses overall were positive. |
| ^35^Wyer, Jackson, Iedema, Hor, Gilbert, Jorm, Hooker, O’Sullivan, & Carroll  2015  (Australia) | Explores patients’ experiences, comprehension & actions of Infection Prevention and Control (IPC). | Video-reflexive ethnography (study was nested with within a large multi-method study) | 14 participants were videotaped & 8 of those then took part in the reflexive sessions of whom six had experienced a healthcare associated infection). Reflexive sessions were either audio or videotaped. | Most were not aware of the extent to which MRSA was in the hospital, and had not thought of their own risk of acquiring an infection. In watching the videos, patient came to see gloves as representing safety. Two patients initially stated they had no role to play [one stated, “Me? I can’t really do much”], but it became clear through discussing the videos that they had practiced IPC – they reported washing their hands, maintaining personal hygiene, following rules, & speaking up when seeing substandard IPC practices. There were participants who wondered about spreading infection but did not ask questions, and not everyone questioned inconsistent practices when they say them. Fear of offending, possible negative repercussions, or feeling their attempts to engage would be negated. Participants developed their own strategies to protect themselves – learning from the Internet; talking with other patients; listening to nurses educate each other; observing practices and forming ideas based on that. IPC was of lesser concern for some, but others were more vigilant. |
| **Author(s) & Year** | **Study Objective(s)** | **Design** | **Sample** | **Relevant Findings** |
| ^36^Seale, Chughtai, Kaur, Crowe, Phillipson, Novytska, & Travaglia (2015)  (Australia) | Pilot study to examine receptiveness of patients toward empowerment tool to increase their awareness and participation in preventing health-care associated infections. | Prospective controlled intervention study.  (Intervention included one-to-one consultation & brochure given with slogan of ask questions, speak up, and be proactive. | 60 postoperative patients.  Patients were surveyed twice (baseline and after discharge), and questions included their willingness to ask providers questions. | At baseline, 38 of 60 participants reported they would be highly willing to be involved with a program to prevent infections. They were also more willing to ask factual questions as opposed to challenging ones. In the follow-up survey, 31 of 48 (remaining 12 either refused or not able to contact) felt encouraged to tell staff about concerns; & 68% agreed with the statement that they were confident they can help prevent or reduce hospital infections– there were no differences between groups in either regard. Further, 23 participants reported notifying staff of a concern (e.g. redness around wound), only 3 reported asking about provider handwashing. Fifteen reported asking a family member to ask a question on their behalf; 10 had taken notes; 8 asked staff about infections - there were no difference between groups in regard to these behaviours. From the intervention group, only 9 recounted the message about encouraging patients to play a role, the remaining speaking only about the need for infection control. |

Table D

***Patient Behaviours*** *– Blood Transfusion (n=1)*

| **Author(s) & Year** | **Study Objective(s)** | **Design** | **Sample** | **Relevant Findings** |
| --- | --- | --- | --- | --- |
| ^37^Davis, Vincent, & Murphy (2011)  (UK) | Examine evidence related to patient involvement to reduce blood transfusion errors. | Literature Review | 15 articles of relevance. | The authors noted opportunities exist for patient involvement, but what remains unclear “how able and willing” patients would be to be engaged in this way. They do acknowledge some evidence of patients admitting that they did check the unit of blood they were given to see if it was the correct blood group, while another example was provided wherein a patient was protecting his IV site and watching the transfusion drip to ensure his vein did not collapse. |

Table E

***Patient Behaviours*** *– Advocacy – General (n=1)*

| **Author(s) & Year** | **Study Objective(s)** | **Design** | **Sample** | **Relevant Findings** |
| --- | --- | --- | --- | --- |
| ^38^World Health Organization (2013) (Switzerland) | *“Patients for Patient Safety – Partnerships for Safer Health Care”.* | Opinion paper | The document is to promote patient engagement in safety. They acknowledge that “patient safety is a global health issue” (p. 3), and that “every year millions of patients are harmed by unsafe health care” (p. 3). Patient and community engagement remains a core priority of the WHO Patient Safety. They argue that patients and families are an untapped resource, and are the only people present through the continuum of care, giving them unique insight and knowledge. Patient advocates are profiled. | |
| ^39^+Ocloo  (2010)  (UK) | Examines patients’ views about medical harm and patient safety reforms. | Participatory Action research (observations; interviews; questionnaires) | Self-help groups (14) (Medical Harm Self-Help Network-MHSHN; & 21 participants from 2-day residential program (Break Through Programme) | MHSHN participants had strong views on doing something but did not feel involved with reform. The network chose to focus on lobbying for change. A noted theme, once harm had occurred, despite their diversity, was to get answers and explanations. The participants’ stories in the Break Through Programme (aimed at enabling individuals develop ways of managing psychological effects of medical harm) are shared, and the researcher notes how they tool wanted to see more political strategies for change. The immediate harm of the incident as well as the after effects of the incident and how individuals felt toward the system. |
| ^40^+Kovacs Burns  (2008)  (Canada) | To share the stories of 25 Canadian patients & family members who are Canadian Patient Safety Champions. | Discussion paper with patient/family stories. | 25 Patient Safety Champions | The author details select personal patient/family stories using direct quotes. Care after an adverse event is described. The author suggests that generally there is growing interest for patients and families in becoming actively involved in discussions and actions related to safety, & the descriptions of the stories of these patient/family Safety Champions & how they are engaged as advocates is indication of their involvement at policy level. |

+This study found in Doherty and Stavropoulou (2012) Systematic Review noted for information but not repeated in final set count.

Table F

***Patient Behaviours*** *– Advocacy – Parents/Others (n=5)*

| **Author(s) & Year** | **Study Objective(s)** | **Design** | **Sample** | **Relevant Findings** |
| --- | --- | --- | --- | --- |
| ^41^Anderson, Bradford, & Clark  (2018) (Australia) | To understand and describe the lived experiences of parents of children with cancer who received treatment for fever with confirmed or suspected neutropenia. | Qualitative using descriptive phenomenological concepts | 9 participants (5 children treated in the tertiary treating center and 4 treated in smaller regional towns) | Parents’ experiences were related to the level they needed to advocate for their child’s care. Familiarity with HCP increased confidence and improved the parents experience. Important themes included:   - Being heard - Anticipated distress and uncertainty - Confidence in healthcare professionals     Interviews demonstrated parents developed expertise on managing their child’s cancer. |
| ^42^Oyesanya & Bowers  (2017)  (USA) | To describe family caregivers’ experience of visitors while their family member (the patient) (with moderate-to-severe traumatic brain injury) is hospitalized. | Qualitative study using grounded theory | 16 family caregivers; every participant interviewed at least once (n=8 were interviewed twice) | Caregivers oversaw ‘welcome’ and ‘unwelcome’ visitors to protect the patient’s physical and emotional safety, as well as to conserve their own energy. There was limited staff involvement with unwelcomed visitors. Managing unwelcome and welcome visitors took a significant amount of energy from family caregivers. “Protecting the patient’s physical safety focused on preventing re-injury, fatigue, or overstimulation; protecting the patient’s emotional safety focused on stabilizing the patient’s emotional state.” (p. 282) |
| ^43^*Rosenberg, Rosenfeld, Williams, Silber, Schlucter, Deng, Geraghty, & Sullivan-Bolyai  (2016)  (USA) | To explore parents’ perspectives about their involvement in safety for their hospitalized children. | Qualitative, descriptive study | 10 mothers & 2 fathers (recruited from the medical-surgical unit of a 109-bed children’s service); interviews & observations | “Four consistent themes emerged from analysis: (1) Parents identify potential risks to safety, including behavioral, communication, & environmental lapses; (2) parents describe their hospital role as an extension of their “home” role as protectors; (3) parental participation in safety practice varies by individual (exhaustion, familiarity with US health care social norms) & organizational (hospital culture) factors; & (4) parents continually consider a balance between ensuring child comfort & safety, & speaking up & risking damage to relationships with clinicians that could affect care or interactions for child and family.” (p. 320) “Parents viewed their primary role in the hospital as protectors…This responsibility is both their right & their job.” (p. 321) |
| **Author(s) & Year** | **Study Objective(s)** | **Design** | **Sample** | **Relevant Findings** |
| ^44^Sandlin-Leming  (2010)  (USA) | Discussion paper, with patient story, about pediatric patient safety. | Discussion paper | The author shares the story of Josie King (18 month old), admitted for burns. She died two days before she was supposed to go home, from extreme dehydration and misused opioids. Her mother’s voiced objectives to the nurse giving her opioids was not heeded. Josie’s mother had asked questions and did note everything, yet providers had not listened. In the aftermath, Josie’s parents donated a portion of the settlement money to the hospital to create a safety program in her name (Josie King Foundation). Josie’s mother does some public speaking about this tragic event and has written a book which supports the Foundation. The author also provides strategies for empowering parents to be vigilant. | |
| ^45^+Tarini, Lozano, & Christakis (2009)  (USA) | Determine proportion of parents concerned about errors during child’s hospitalization and association with self-efficacy with physician interactions. | Cross-sectional survey | 130 parents of children admitted to general medicine service of tertiary care hospital. (Surveyed within 48 hours of child’s admission but after speaking with care team). | 63% agreed/strongly agreed that they felt the need to watch over their child’s care to make sure mistakes are not made. A parent’s self-efficacy interacting with physicians was associated with less parental concern for error. |
| ^46^Clarke & Fletcher  (2004)  Canada) | Study of parent experiences with a child diagnosed with cancer. | Qualitative study (telephone interviews by first author whose own daughter had cancer) | 29 parents of children who had been diagnosed with cancer. (sample is described as a non-representative volunteer & quota sample. | Interviews varied in length from one to four hours. One major theme identified was ‘surplus suffering’ which was equated to system problems. Parents perceived mistakes or delays in diagnosis, carelessness or lack of kindness. This resulted in the parents viewing the need to be on constant guard & intervening at times. Illustration of findings: “I was prepared for a fight actually, saying ‘she needs to have blood drawn’”. |
| ^47^+Hurst  (2001)  (USA) | Exploring mothers’ actions to safeguard their hospitalized baby. | Critical ethnography (observations of participants & open-ended interviewing) | 12 mothers with hospitalized premature baby. (tertiary level NICU) | 448 hours of observation. “First and foremost among mothers’ actions was vigilant watching over” (p. 43). Seven actions were identified that mothers used to provide for safety and protect against harm, including “judicious use of challenging institutional authority” (p. 44), as well as supportive relationships with other mothers (e.g. information, validation); seeking out a person of higher organizational authority; uses her direct observation of provider for learning when questioning or asking. |

+This study found in Doherty and Stavropoulou (2012) Systematic Review is noted for information but not repeated in final set count.

*This study is included in Patient Attitudes table given items of relevance but only counted once in final set count.

Table G

***Patient Behaviours*** *– Handwashing (n=29)*

| **Author(s) & Year** | **Study Objective(s)** | **Design** | **Sample** | **Relevant Findings** |
| --- | --- | --- | --- | --- |
| **Systematic Reviews & Literature Reviews** | | | | |
| ^48^Alzyood, Jackson, Brooke, & Aveyard (2018)  (UK) | “The aim of this review was to review patients’ and healthcare providers’ perceptions towards patient involvement in promoting hand hygiene compliance in the hospital setting?” (p. 1330) | Integrative literature review | 19 papers included in this review of 240 identified.  (data from Europe, Australia, North America, and Asia) | - Patients were willing to remind health care professionals (especially nurses) to wash their hands - Some patients will prompt HCPs but many were reluctant to do so suggesting patients are less willing to ask challenging questions - Cross-cultural variations were reported - Although some healthcare providers are accepting of patient involvement in hand hygiene, others felt negatively about being asked to perform hand hygiene   This strategy of patient engagement can be challenging for patients and HCP but needs further exploration. |
| ^49^Butenko, Lockwood, & McArthur  (2017)  (Australia) | “To determine the best available evidence in relation to the experiences of the patient partnering with healthcare professionals for hand hygiene compliance” (p. 1645). | Qualitative systematic review | 5 studies retrieved with 3 of these studies included for qualitative synthesis | Two final findings discovered:   - Organizational structures enable partnering between healthcare professionals and patients for hand hygiene compliance, however the culture, beliefs, and behaviours of HCP and patients for not fully support this partnership.   Patients have differing levels of knowledge and balance partnering hand hygiene against possible detrimental impacts on the caring relationship provided by healthcare professional, out of concern for their own well-being, health outcomes, treatment, and/or recovery. |
| ^50^Butenko, Lockwood, & McArthur (2015)  (Australia) | Protocol for a systematic review to understand the experience of partnership between health professionals and patients related to hand hygiene compliance in acute adult hospital setting. | Systematic review protocol |  | See same author team (2017) for completed review. |
| **Author(s) & Year** | **Study Objective(s)** | **Design** | **Sample** | **Relevant Findings** |
| ^51^Davis, Parand, Pinto, & Buetow (2015)  (UK) | Review of the evidence on effectiveness of strategies aimed at increasing patient involvement in reminding healthcare providers about hand hygiene. | Systematic Review | 28 articles (23 on evaluations of the effectiveness of developed patient-focused strategies & 5 on patients’ attitudes toward hypothetical strategies). | A number of strategies available to encourage patients to question providers about handwashing. Healthcare provider encouragement seemed to be the most effective strategy for their engagement in this practice. Methodological quality generally weak. The authors also caution that most of the findings were based on intention versus actual behaviour, and intention does not always lead to action when confronted with the reality of the situation. |
| **Additional Articles of Relevance** | | | | |
| ^52^Li, Liu, Zeng, Chen, Mo, & Yuan  (2019)  (China) | To understand patients’ practice of hand hygiene (HH) & their knowledge and attitudes. | Cross-sectional survey study | Inpatients and their family members or caregivers (single centre).  310 completed the survey | “A total of 62.3% of patients washed their hands <5 times a day and 49.0% spent <1 min every time. With regards to the seven steps of handwashing, 96.45% of the respondents adhered to the first step (washing the palms), but only 20.6% adhered to the fifth step (thumbs) and 17.7% to the sixth step (fingertips). Most respondents washed their hands only when visibly dirty. Few patients washed their hands before drinking fluids, and before and after interacting with visitors. HH compliance was lower among intensive care unit patients than medical patients.” (p. 1) |
| ^53^Sande-Meijide, Lorenzo-Gonzalez, Mori-Gamarra, Cortes-Gago, Gonzalez-Vazquez, Moure-Rodriguez, & Herranz-Urbasos (2019)  (Spain) | To examine the attitudes & perceptions of patients & health care workers (HCWs) & to understand the method they consider most suitable for implementing an effective patient participation program to improve hand hygiene (HH). (p. 46) | Cross-sectional survey study | 337 patients & families (&  196 healthcare workers) | “More than half of surveyed patients and their family members (58%) answered that they normally observe whether HCWs or their caregivers at home wash their hands. Most of them would feel better if they knew that HCWs had performed HH. Finally, 49.9% (48% of patients and 53% of family members) would be willing to ask HCWs if they had cleaned their hands before administering care.” (p. 46-47) “The preferred method for patient participation in HH promotion was to place posters on wards (86% of patients & their family members & 74% of HCWs).” (p. 47-48) |
| **Author(s) & Year** | **Study Objective(s)** | **Design** | **Sample** | **Relevant Findings** |
| ^54^Knighton, Dolansky, Donskey, Warner, Rai, & Higgins  (2018)  (USA) | “…Test the influence that a novel verbal electronic audio reminder [EAR] would have to an educational patient hand hygiene bundle to improve patient hand hygiene in older adults hospitalized for elective lower extremity orthopedic or podiatry surgery...” (p. 611) | 2-group comparative effectiveness study | 75 participants in total  41 were exposed to bundle with EAR; 34 were exposed to bundle without EAR | The addition of a novel verbal electronic audio reminder to a patient hand hygiene bundle resulted in a significant increase in patient hand hygiene performance. Such an intervention can improve patient hand hygiene while minimizing an increase in health care worker workload. “Although participants that received the verbal electronic reminder did use more alcohol-based hand sanitizer product, both groups demonstrated some level of hand hygiene behavior, indicating that patients are capable of practicing themselves.” (p. 613) |
| ^55^Cheng, Wong, Wong, Chau, So, Wong, Chen, Lee, Tai, Chau, Lo & Yuen  (2017)  (China) | Conducted a pilot study to assess the knowledge on hand hygiene, perception, and acceptance of patient empowerment in hand hygiene promotion among hospitalized patients and health care workers. | Pilot patient empowerment program | 202 patients (pre) & 167 patients (post) | A positive response from the health care workers was reported in 70 (93.3%) of 75 patients who reminded health care workers to clean hands as part of the empowerment program. A significant increase in volume of alcohol-based handrub consumption was observed during the intervention period compared with baseline.  HCWs supportive of the program had significantly better knowledge of hand hygiene practice (147 out of 149 [98.7%] vs 21 out of 42 [50.0%]; P < .001). Among hospitalized patients, 196 (97.0%) patients had never asked HCWs to clean their hands, but 103 of 196 patients (52.6%) showed support for the program. The number of patients willing to be empowered increased significantly to 127 (64.8%; P < .001 by McNemar’s test) after encouragement by ICNs.  During the intervention period, 502 patients were hospitalized in the intervention wards. Two hundred twenty-three patients were eligible and 167 (74.9%) of these patients agreed to participate in the program.  During the empowerment program, 75 patients reminded HCWs to clean their hands, and 70 of 75 patients (93.3%) reported a positive response from the HCWs. Postintervention interviews of 114 HCWs reported a 73.7% (n = 84) use of visual aids and 19.3% (n = 22) use of verbal reminders by the empowered patients. |
| **Author(s) & Year** | **Study Objective(s)** | **Design** | **Sample** | **Relevant Findings** |
| ^56^Desai, Rezmovitz, Manson, Callery, & Vearncombe  (2017)  (Canada) | “The purpose of this QI study at Sunnybrook Health Sciences Centre (Sunnybrook) Family Practice was twofold: 1. To investigate whether it was feasible to use patients as observers who could observe all four moments of HH as observation of moments 2 and 3 by auditors can be challenging and 2. To determine if target HH compliance rates of 95% were being achieved in the Family Practice Unit using said patients as observers.” (p. 152) | Quality Improvement (4 cycles) | Range of 137-242 surveys were completed. The range for surveys being completed correctly by the patient observers were 79.6% to 97.9%. | Since the start of the patients as observers program, hand hygiene compliance was found to be 94-97%, which is maintaining the target of 95%. Involving patients as observers is a feasible and beneficial way to monitor hand hygiene compliance in an ambulatory care setting. May help reduce costs, engage patients in their healthcare, and improve communication between patients and providers. |
| ^57^Doyle, Xiang, Zaman, Neiman-Hart, Maroon, Arghami, Durani, Salana, Komakula, & King  (2017)  (USA) | To examine hand-washing rates in an outpatient clinic and explore whether a “co-washing” approach would be a benefit. | Quality Improvement study (2 phases/cycles) with a quasi-experimental design | 384 questionnaires (184 from phase 1 and 200 from phase 2) | Patients endorsed hand washing and participated in hand washing 83.7% of the time and there was no significant different in clinicians hand washing after the co-washing began. There were more positive responses to “did the nurse or doctor encourage you to wash your hands?” and “did you wash your hands?” from patients after the co-washing. |
| ^58^Haverstick, Goodrich, Freeman, James, Kullar, & Ahrens  (2017)  (USA) | To improve patients’ hand hygiene through the promotion and use of hand washing with soap and water, hand sanitizer, or both and improve patients’ education to reduce hospital acquired infections. | Quality improvement project – pre and post intervention survey | 33 staff responses  Patients: 16 responses before the intervention, 39 responses 1 months after intervention, 63 responses 2 months after intervention, and 54 responses 3 months after the intervention. | After the intervention, rates of infection declined and patients reported staff offering more opportunities for and encouraging hand hygiene.  “After the intervention, patients’ knowledge about the importance of hand hygiene and the availability of hand hygiene supplies increased. Patients’ responses indicated that they were offered the opportunity to wash their hands before meals and after using the restroom with increasing frequency in the months following the intervention. Therefore, compliance with patient hand hygiene would have increased.” (p. e6) |
| **Author(s) & Year** | **Study Objective(s)** | **Design** | **Sample** | **Relevant Findings** |
| ^59^Lastinger, Gomez, Manegold, & Khakoo  (2017)  (USA) | To examine the attitudes of adult patients, parents of pediatric patients, and physicians toward a new patient empowerment tool. | Cross-sectional survey | 114 parent surveys completed; 108 surveys from adult patients;  89 surveys from physicians | “Most parents (77.0% for physicians & 81.4% for nurses) & most adult patients (64.8% for physicians & 71.2% for nurses) felt comfortable using the PET to remind health care workers to perform hand hygiene. Of participants who did not feel comfortable, the most common reason was shyness.” (p. 826) |
| ^60^Ong, Tan, Yeo, & Goh  (2017)  (Singapore) | The objectives were: (1) All patients receive the MRSA patient information leaflet on admission or transfer to the ward; (2) All patients are able to verbalize that both patients & healthcare providers should perform hand hygiene to prevent HAIs; (3) All nurses document that the patient education about hand hygiene has been given (p. 23). | Pre-post audit strategy | 54 patients from two orthopedic units | The percentage of patients who demonstrated knowledge about the importance of hand hygiene improved by 48.1%. Patients were able to have more ownership of their wellbeing through encouragement of patient participation. Pre- and post-implementation audits are a viable method to implement change & translate evidence to practice regarding hand hygiene.  Barriers included patient readiness for information & sustained compliance regarding patient education. |
| ^61^Pokrywka, Buraczewski, Frank, Dixon, Ferrelli, Shutt, & Yassin  (2017)  (USA) | To assess patient hand hygiene (PHH) practice; to improve the opportunities for PHH though staff education & patient assistance; as well as to determine if improving PHH opportunities would impact Clostridium difficile (CD) infection (CDI) rates. | Bi-phasic, quasi-experimental, single-center study | Phase 1 (4 medical-surgical units): 97 patients pre- education & 291 patients post-education  Phase 2: 80 patients in March 2015 (after hospital-wide implementation of initiative) & 189 patients in March 2016 | Patient hand hygiene opportunities improved significantly after staff and patient education, and CD standardized infection ratios decreased significantly for 6 months post-intervention, however increased in the last quarter of the year of implementation. PHH opportunities can be increased with education & opportunities for patients to clean their hands. “Sustained PHH requires participation of staff to engage the patient with opportunities, reminders, and encouragement to keep their hands clean.” (p. 963) |
| **Author(s) & Year** | **Study Objective(s)** | **Design** | **Sample** | **Relevant Findings** |
| ^62^Rai, Knighton, Zabarsky, & Donskey  (2017)  (USA) | To determine the impact of a 5 moments of patient hand hygiene educational intervention (this included modeling hand washing when a provider enters the room) on performance of patient hand hygiene. | Randomized trial | 54 patients (28 in the intervention group; 26 in the control group) | “The intervention group used signiﬁcantly more hand sanitizer than the control group overall and for each of the 3 days of monitoring. Of 342 total hand hygiene observations, 160 were in the intervention group and 182 were in the control group. The intervention group performed hand hygiene on entry of personnel signiﬁcantly more often overall than the control group, but the difference was only statistically signiﬁcant on day 1 as performance decreased on days 2 and 3. The percentage of patients who performed hand hygiene on entry of personnel during at least 1 observation was signiﬁcantly higher in the intervention versus control groups.” (p. 552-3) |
| ^63^Sunkesula, Kundrapu, Knighton, Cadnum, & Donskey  (2017)  (USA) | To test the hypothesis that a patient hand-hygiene intervention would reduce new acquisition of hand contamination in hospitalized patients. (p. 595) | Non-blinded parallel randomized trial | 95 patients (47 in the control group; 44 in the intervention group) | “For patients with negative hand cultures on admission, we demonstrated that recovery of healthcare-associated pathogens from hands was significantly reduced in those receiving a patient hand-hygiene intervention versus those receiving standard care.” (p. 596) |
| ^64^Caine, Pinkham, & Noble  (2016)  (USA) | Evaluate hand hygiene rates on a medical surgical unit | Quasi-experimental pre- and post-test design | 161/166 pre  153/166 post/ | 65% of patients reported seeing or hearing staff perform hand hygiene before the intervention compared to 93% after. It was found that adding an auditory cue was significant enough to be reflected in patient feedback and engaged patients. |
| ^65^Cheng, Tai, Li, Chau, So, Wong, Ching, Ng, Ho, Lee, Lee, Wong, & Yuen  (2016)  (China) | Observe the effects of an education campaign targeted to increase patients self-initiated hand hygiene, and a hand hygiene ambassador-initiated directly observed hand hygiene program on compliance. | Descriptive study | 582 patients were observed | The audited compliance of patient-initiated hand hygiene was 37.5%, with 26.9% before meals or medication, 27.5% after using a urinal or bedpan, and 89/7% after using the washroom. Ambassador initiated directly observed rates of hand hygiene were 97.3%, which is significantly higher than patient self-initiated hand hygiene via an education program. |
| **Author(s) & Year** | **Study Objective(s)** | **Design** | **Sample** | **Relevant Findings** |
| ^66^Stewardson, Sax, Gayet-Ageron, Touveneau, Longtin, Zingg, & Pittet  (2016)  (Switzerland) | To assess the effect of enhanced performance feedback & patient participation on hand hygiene compliance in the setting of multimodal promotion. (p. 1345) | Single-centre, cluster randomized controlled trial | 67 wards assigned as:  21=Control;  24=Enhanced performance feedback;  22=Enhanced performance feedback plus patient participation (including, but not limited to, asking providers about handwashing) | There was a 3% difference in the enhanced performance group & a 4% difference in the enhanced performance group plus patient participation when compared with the control (neither clinically significant effect when compared with the control). “Observers witnessed no episodes of patients reminding health-care workers to perform hand hygiene during hand hygiene opportunities before patient contact.” (p. 1351) Overall, in all 3 groups hand hygiene compliance was significantly higher than the baseline. Researchers proposed the improvement may be related to cross-contamination, highlighting the challenges of randomised trials of behaviour change. |
| ^67^von Lengerke, Kroning, Lange, & Lower Saxon Diabetes Outpatient Centres Study Group  (2017)  (Germany) | “This study scrutinizes intentions of patients with type-2-diabetes (T2D) to speak up for HCPs’ hand hygiene during inpatient foot treatment, and whether this motivation increases if they envision encouragement by hospitals inviting them to speak up. Also, differences by hand hygiene knowledge, socio-demographic and diabetes-related factors are examined.” (p.1138). | Cross-sectional questionnaire-survey | 473 patients participated | “N=177 (41%) strongly intended to speak up… Institutional encouragement was associated with an increased rate of strong… and higher mean intention…” (p. 1137). School education and knowledge of hand hygiene practices was associated with differences in intention to speak up. Education is key to patient empowerment. |
| ^68^Busby, Kennedy, Davis, Thompson, & Jones  (2015)  (USA) | Explore patients’ understanding of effective hand hygiene used in the hospital environment. | Descriptive study | 90 adult patients (general medicine) (These patients were assigned to one of three groups: staff washed with: soap & water only; waterless alcohol-based hand cleaner; or a combination of methods) | 90% and 93% in the handwashing only and the sanitizer only group, respectively, indicated caregivers always cleaned their hands. In the combination group, 80% of patients reported staff always cleaned their hands. Some patient comments included: “why are you washing your hands so much?” and “if you wash your hands any more, they’re going to all off” – to which the authors suggest lack of understanding. There were patients who did not notice if staff washed at all (3.3%, 6.7%, & 6.7% in each of the 3 groups). One patient was awakened by the nurse washing her hands, which she verbalized what she was doing as well, yet he still reported not noticing staff wash. |
| **Author(s) & Year** | **Study Objective(s)** | **Design** | **Sample** | **Relevant Findings** |
| ^69^Kim, Nam, Na, Shin, Lee, Kim, Kim, Song, Choe, Park, Bang, Kim, Park, Kim, Oh, & Kim (2015)  (Republic of Korea) | Explore perceptions of patients/families & providers with regard to patient participation in hand hygiene. | Cross-sectional survey | 334 patients/families (teaching hospital) | Significant discrepancy in perceptions regarding patient participation between patients/families and healthcare workers. Approximately 60% of patients/families reported that they usually observed whether providers cleaned their hands. More than 75% of patients and 84% of the families felt they should be aware whether providers wash; and 3/4 of respondents wished to ask providers to clean but approximately 6/10 in both patients and families when intent was examined. |
| ^70^McGuckin & Govednik (2014)  (USA) | Investigation hand hygiene compliance knowledge and perceptions, information sources on compliance rates, and past behaviour in asking providers to wash their hands. | Survey (via Internet) | 1,016 US consumers | 17% had asked a provider to perform hand hygiene. Of those who asked, 57% reported ‘no particular source’ or ‘word of mouth’ as their information source. 24% of those who asked cited, doctors, nurses or other providers or hospital pamphlets as their information source. Of the entire group, 50% believed hand hygiene compliance was 75%, while 19% believed it was 51%-75%. |
| ^71^Le-Abuyen, Ng, Kim, De La Franier, Khan, Mosley, & Gardam  (2014)  (Canada) | Patient observation of provider hand hygiene. | Survey pilot | Patient returned 381 audit cards in family practice health centre | Patients returned 75% of the survey cards (381/507) indicating whether staff had cleaned their hands before physical contact. Patients expressed satisfaction in being part of the process (majority of patient commentary n=74). |
| ^72^Pan, Tien, Hung, Lin, Yang, Yang, Wang, Chang, & Chen  (2013)  (Taiwan) | To assess hand hygiene knowledge and attitudes and intentions about patient empowerment among patients/families and providers. | Cross-sectional survey using questionnaires | 345 patients/family members & 880 providers  (tertiary teaching hospital) (interviewers conducted questionnaire) | 95% of patients/families had positive attitudes regarding patient empowerment, but only 67% had intention to remind providers about handwashing. (Attitude was assessed with the question: Do you think patients or family can help remind providers about hand hygiene? Intention, for patients, was assessed with question: Would you remind providers who do not perform hand hygiene?) |
| ^73^Rogers (2013)  (Canada) | Description of one teaching hospital’s plans to develop hand hygiene resources for patients and family members to encourage them to wash their hands. | Survey  (Survey to understand what patients may or may not know about handwashing) | Current and former patients who are part of the organization’s Virtual Patient Focus Group (nearly 300). | 60 surveys returned. Almost unanimously that they understood the importance of handwashing. Notably, hospital outpatient managers did not think that most patients did know the importance of handwashing. The respondents knew the important times to wash, and ways of washing. The respondents identified that the most effective ways to encourage patient handwashing was: signage; volunteer prompting in kind respectful way, and funneling patients through pathways so that had to wash. |
| **Author(s) & Year** | **Study Objective(s)** | **Design** | **Sample** | **Relevant Findings** |
| ^74^Reid, Moghaddas, Loftus, Stuart, Kotsanas, Scott, & Dendle (2012)  (Australia) | To determine the effectiveness a brochure encouraging patients to ask providers about handwashing, and to determine which provider characteristics affect patients’ willingness to ask. | Prospective pilot program (brochure distributed & follow-up interview done) | 46 patients (program conducted on 7 medical and surgical units in tertiary hospital) | After reading the brochure, all believed that they should have a role in their own safety which included hand hygiene. 93% believed that should be involved in improving hand hygiene. Patients were less willing to ask a doctor to clean their hands than a nurse. Patients reported they would ask doctors to clean their hands 43% of the time, and nurses 67% of the time. The authors note the difference between patients’ wanting a role and willingness to ask. |
| ^75^Ciofi degli Atti, Tozzi, Ciliento, Pomponi, Rinaldi, & Raponi  (2011)  (Italy) | Evaluate perceptions of healthcare workers and parents about hand hygiene and effectiveness of measure for improving hand hygiene adherence. | Cross-sectional study using self-administered questionnaires | 139 provider questionnaires & 236 parents questionnaires (completed in hospital) | Parents reminding provider to perform hand hygiene was perceived at the least useful action for 55.7% of parents. |
| ^76^McGuckin, Waterman, & Shubin (2006)  (USA) | Consumer attitudes about hand hygiene and healthcare-acquired infections. | Survey (telephone) | 1008 participants. | 94% rated environmental cleanliness as very important. 85% considered low infection rates very important in hospital selection. 80% of consumers said they would ask their provider to wash or sanitize his/her hands if the provider explained the important of this to them. |

Table H

***Patient Behaviours*** *– Medication Safety (n=13)*

| **Author(s) & Year** | **Study Objective(s)** | **Design** | **Sample** | **Relevant Findings** |
| --- | --- | --- | --- | --- |
| **Systematic Reviews & Literature Reviews** | | | | |
| ^77^Tobiano, Chaboyer, Teasdale, Raleigh, &  Manias  (2019)  (Australia) | “To synthesise peer-reviewed research evidence concerning patients’ perceptions of how they engage in admission and discharge medication communication, and barriers and enablers to engagement in medication admission and discharge communication.” (p. 87) | Systematic mixed studies review | 15 studies included in review | 3 themes: “In the ﬁrst theme ‘desiring and enacting a range of levels of engagement’, patients displayed medication communication by taking responsibility for sharing accurate medication information, and by seeking out different choices during communication. The second theme ‘enabling patients’ medication communication’ uncovered various strategies to promote patients’ medication communication, including informing and empowering patients, and encouraging family involvement. The ﬁnal theme, ‘barriers to undertaking medication communication’ included challenges enacting two-way information sharing and patients’ preference.” (p. 87) |
| ^78^Kim, Suarez-Cuervo, Berger, Lee, Gayleard, Rosenberg, Nagy, Weeks, & Dy  (2018)  (USA) | To assess how patient and family engagement strategies are implemented and evaluated in the context of medication safety. | Systematic review | 19 studies | Interventions included educational strategies (booklets, learning modules, training program) and medication reconciliation strategies (eMARs, medication reconciliation cards, brings medications into the office).  *[Ladder of engagement used: 1) Inform about healthcare; 2) Inform about engagement; 3) Empower; 4) Partner; 5) Integrate)].* The median level of engagement consisted of informing about engagement with health care providers (level 2). The findings ranged from level 2 to level 4, with level 3 being empowering patients with communication tools and skills, and level 4 being partnering with patients in their care.  In the 11 studies with control groups, 55% reported statistically significant improvement on at least one medication safety outcome.  Key strategies for engaging patients in medication safety are education and medication reconciliation. |
| **Author(s) & Year** | **Study Objective(s)** | **Design** | **Sample** | **Relevant Findings** |
| **Additional Articles of Relevance** | | | | |
| ^79^Wang, Zhang, Zhang, Zhu, & Yan  (2019)  (China) | “To develop and test the psychometric characteristics of the Inpatients' Involvement in Medication Safety Scale.” (p. 1648) | Scale development & testing | “The scale was formulated through literature review, semi‐structured interviews and Delphi expert consultation. A group of 461 inpatients from a tertiary hospital were selected to examine the reliability and validity of the scale.” (p. 1648) | “The scale consisted of three dimensions and 23 items.” (p. 1648)  “The Inpatients' Involvement in Medication Safety Scale has good reliability and validity and can be used to evaluate inpatients' involvement in medication safety.” (p. 1648) |
| ^80^Phipps, Giles, Lewis, Marsden, Salema, Jeffries, Avery, & Ashcroft (2018)  (UK) | To examine ways in which patients might enable ‘mindful organizing’ with medication safety in primary care settings (p. 966) | Qualitative study with focus groups and interviews | 126 participants | Four themes were identified to explain patient behaviour associated with ‘mindful organizing’: knowledge about clinical or system issues, artefacts that facilitate control of medication risks, communication with health-care professionals, and the relationships between patients & the healthcare system. ‘Mindful organization’ can potentially be useful for framing patient involvement. “From a mindful organizing perspective, patient involvement might be conceptualized as a set of interactions between patients and health-care professionals that maintain collective “mindfulness” about safety issues, that is an awareness of potential or impending patient safety hazards and a capacity for acting on such insights.” (p. 966) |
| ^81^Prey, Polubriaginof, Grossman, Masterson Creber, Tsapepas, Perotte, Qian, Restaino, Bakken, Hripcsak, Efird, Underwood, & Vawdrey  (2018)  (USA) | “... to investigate whether an electronic home medication review tool can engage patients in the medication reconciliation process and allow them to contribute information to their home medication lists upon hospital admission.” (p. 1461) | Pilot study | 65 patients participated (36 in the *Before* group; & 29 in the *After* group) | The results showed that both patient groups (those who used the tool before and after hospital admission), had a high willingness to engage in medication reconciliation, and it shows that patients were able to identify important medication discrepancies and changes that may have been missed by healthcare professionals. |
| **Author(s) & Year** | **Study Objective(s)** | **Design** | **Sample** | **Relevant Findings** |
| ^82^Schopf, von Hirschhausen, Farin, & Maun  (2018)  (Germany) | To understand the perceptions of elderly patients & their general practitioners (GPs) regarding communication about polypharmacy & medication safety, as well as empowerment approaches patients and GPs identify  (p. 356) | Exploratory, qualitative study using interviews | 6 patients;  3 general practitioners (GPs) | Three themes included differing medication plans & the possible causes; dialogue concerning medication & whose responsibility is it; & how to support patient’s engagement. GPs felt that patients would not always report or possibly conceal information, yet patients felt they could be open with providers. Patients’ awareness of the significance of their active role needs to be increased, and includes issues such as trusting your GP does not preclude asking questions or seeking information.  “Both GPs and patients could name very few ways in which patients could be supported to become more informed and active in communication concerning poly-pharmacy and medication safety.” (p. 355) |
| ^83^*Garfield, Jheeta, Husson, Lloyd, Taylor, Boucher, Jacklin, Bischler, Norton, Hayles, & Franklin  (2016)  (UK) | To explore hospital inpatients’ involvement with medication safety-related behaviours, facilitators and barriers to this involvement, as well as electronic prescribing impact. | Ethnographic study using observations and interviews (two hospital organizations) | Interviews with 12 patients & 6 patient carers (as well as nurses, doctors & pharmacists); observation during specific events ranged from 30 patients to 226 patients | In 4 of 247 (2%) cases, paper or electronic medication records were shown to patients. “Both healthcare professionals & patients identified that patients’ knowledge & beliefs could affect their involvement. Both groups thought that some patients were more knowledgeable & interested in their medication than others & that some were more assertive & others more passive. Some patients described having ‘blind faith’ in healthcare professionals to manage their medication & did not think that their involvement was necessary. Other patients expressed concern that they may upset healthcare professionals & that their care would be affected if they challenged healthcare professionals.” (p. 9) |
| ^84^Heyworth, Paquin, Clark, Kamenker, Stewart, Martin, & Simon  (2014)  (USA) | Pilot study to test a medication reconciliation (med rec) tool delivered via web portal to improve safety for discharged patients. | Mixed method | 60 patients at Veterans Affairs hospital participated in med rec; 10 participants of the 60, randomly chosen for debrief interview by phone. | Outcomes were clinically important medication discrepancies & potential adverse drug events. Comparisons were made between discharge summaries, pharmacist-reconciled med lists & the patients confirmed list. 51 patient messages were sent (remaining 9 ineligible for varying reasons). Response rate by patients to messages sent was 67% (n=34). Of the patient returned messages (n=34), 26 additional clinically important discrepancies were found among 17 patients. Of the 10 interviewed, 90% said they would use the system again, with emphasis on rapid access to provider. |
| **Author(s) & Year** | **Study Objective(s)** | **Design** | **Sample** | **Relevant Findings** |
| ^85^Macdonald,  Heilemann, MacKinnon, Lang, Gregory, Gurnham, & Fillatre  (2014)  (Canada) | To understand, from the perspective of patients and nurses, patient involvement in medication administration safety. | Constructivist Grounded Theory | Interviews with 24 general medicine patients (1-2 weeks following discharge in their home); 17 registered nurses, & 9 licensed practice nurses. (3 different hospitals – two tertiary & 1 community) | Both patients & providers felt patient role in administering medication could be enhanced. Patient role was, for the most part, confirming to the nurse that they recognized the medications they were about to take were the medications prescribed for them. This was an iterative process and was described as ‘confirming delivery’ as perceived by both staff and patients regarding the role of the hospitalized patient. This included 3 sub-processes: engaging in administering the medication (e.g. the degree to which they participated – such as simply swallowing or questioning); being ‘half out of it’ (mental status) (patients knew that at times they were not capable of being involved), and perceiving nursing staff time (patients perceived staff as always busy). Several reported complete provider trust and did not indicate active involvement. Others reported trust because of past experiences. Participants reported that they checked pill quantity, colour and shape, and would ask why they looked different if they did. All but one patient believed in the need for alert patients to have some role; and the one who didn’t believe there was a role, demonstrated later in the interview that she was tracking and monitoring her pills. |
| ^86^McTier, Botti, & Duke (2013/2015)  (Australia) | Explore patient participation in medication management while hospitalized. | Exploratory, descriptive study (mixed method approach) | Before surgery & pre-discharge patient interviews (n=98); 48 observations (involving 40 nurses) & 2 focus groups with providers n=16). (cardiothoracic unit – tertiary hospital) | All patients had changes to their medications. More patients were able to list & state purpose and side effects of medications pre-admission for surgery than pre-discharge. From observation & focus groups, it was evident nurses did not engage patients in medication management. Patients were observed asking questions and seeking information [e.g. “what is potassium for”? Or re: injection “what is it?”]. |
| ^87^Schwappach, & Wernli (2010c)  (Switzerland) | To understand medication errors in cancer care, and the role patients can play in their prevention. | Literature review | Not applicable | MEDLINE and CINAHL searched for articles in English or German between 1990 and 2008.  Obtained survey studies wherein patients observed, detected and reported errors. Note study by Unruh & Pratt (2006) wherein cancer patients with recurring episodes of care identify errors by comparing previous experiences with current one. Evidence suggests patients engage but do not get support of that engagement. Acknowledge that, generally & with cancer patients, little is known about patients’ attitudes, willingness and effectives to engage. “The crucial element is not to push all patients to engage in all aspects of safety, but to provide those able and willing to participate with the necessary information” (p. 290). |
| **Author(s) & Year** | **Study Objective(s)** | **Design** | **Sample** | **Relevant Findings** |
| ^88^Myhre (2007)  (Canada) | To explore patients’ perceptions, attitudes and beliefs about safety practices during medication administration (including but not limited to patient behaviours and perceptions of their participation and accountability in care). (Master’s thesis). | Non-experimental research design (survey) (28-item questionnaire developed by author) | 201 medical and surgical patients | 56% of patients reported they did not see the nurse wash his her hands prior to medication administration. 61% reported providers checked their ID band prior to medication administration. 51% reported checking their medication “the majority or all of the time”, while 42% reported the “rarely” or “some of the time” check (p. 53). 62% believe there is a shared responsibility between nurse/doctor/patient to ensure safe medication administration. |
| ^89^Wright, Emerson, Stephens, & Lennan  (2006)  (UK) | To determine if there is evidence of the benefit of self-administration medicine programs (SAPs) in relation to risk and resource implications. (Notably, of the nine aims of the review, one addresses whether self-administration medicine programs affect the risks associated with medication administration.) | Literature Review | 51 publications included. | In examining medication errors, of two studies, only one found significantly fewer medication errors in the self-administration program group than in controls. Ten of eleven statistically analyzed studies found SAP participants knew more about certain aspects of their medications. The authors state conclusions are hard to make given limited quality studies and details reported within publications. |

*This study is included in Patient Attitudes table given items of relevance but only counted once in final set count.

Table I

***Patient Behaviours*** *– Surgical Safety (n=3)*

| **Author(s) & Year** | **Study Objective(s)** | **Design** | **Sample** | **Relevant Findings** |
| --- | --- | --- | --- | --- |
| ^90^See, Chang, Chuang, Lai, Peng, Jean, & Wang  (2011)  (Taiwan) | Design and evaluate an educational animation program about safety for patients/family to eliminate wrong-site surgery errors. | Single-group pretest and post-test design | 46 patients & 48 family members | Pre-training (past practice of eliminating wrong-site surgery) score 3.6 (scale 1-4) for both patients & family; No significant increase in post scores (anticipated future practice of eliminating wrong-site surgery) for patients, but family improved significantly. |
| ^91^Boyd & Holroyd (2011)  (UK) | Authors examine patient case related to involvement in perioperative marking. | Opinion paper with single case review | One case | The authors describe a case where a patient undertook marking her non-surgical knee noting it as “not this knee”. The authors support patient involvement in perioperative marking but draw attention to the risk of marking the contralateral side. |
| ^92^++Bergal, Schwarzkopf, Walsh, & Tejwani (2010)  (USA) | Examining patient compliance and reliability in marking the surgical site. | Descriptive study | 200 orthopedic patients | Compliance rate= 68.2%. No patient marked the wrong side. The time between enrollment and surgery was significantly different between those who complied versus those who did not. Differences were also seen with regard to age and primary language. There was no statistical difference on a number of demographic variables including: sex, employment status, level of education. |
| ^93^Jeffrey & Curry  (2010)  (UK) | Case/commentary on patient participation in the surgical pause. | Opinion paper with single case review | 1 patient | The patient, wishing no sedation, asked about the surgical pause during her case, and then aloud, proceeded to identify herself and the nature of her surgery. The author then poses questions as to the appropriateness of this as a standard. |
| ^94^+DiGiovanni, Kang, & Manuel  (2003)  (USA) | Examining patient compliance with instructions to prevent wrong-site surgery. | Prospective, descriptive study | 100 patients | 59 marked the extremity correctly; 37 made no mark; & 4 were partially compliant. Patients who had had a previous related surgical procedure had a significantly higher rate of noncompliance. The authors hypothesize as to why this may be, believing that the majority of individuals probably believe nothing wrong will happen. |

+This study found in Doherty and Stavropoulou (2012) Systematic Review is noted for information but not repeated in final set count.

++=additional study found in Berger, Flickinger, Pfoh, Martinez, & Dy (2014) Systematic Review & detailed for its significance but not part of final set count.

Table J

***Patient Behaviours*** *– Communicating (n=8)*

| **Author(s) & Year** | **Study Objective(s)** | **Design** | **Sample** | **Relevant Findings** |
| --- | --- | --- | --- | --- |
| ^95^Bell, Roche, Mueller, Dente, O’Reilly, Sarnoff Lee, Sands, Talmor, & Brown  (2018)  (USA) | Assess patient and family comfort on speaking up about common ICU concerns, identify patient and family perceived barriers to speaking up and to explore factors associated with patient and family speaking up. | Cohort Survey | 105 (of 125) from the cohort of families from a US academic hospital completed the survey    1050 usable surveys from the cohort of families nationally via the internet | 50-70% of families and participants expressed hesitancy to voice concerns about possible mistakes, mismatched care goals, confusing information, and inadequate hand hygiene.    Common barriers to voicing concerns included not wanting to be label a troublemaker, the team is too busy, not knowing who to talk to, being afraid of seeming like they don’t understand medical concepts, and not wanting to harm the relationship with their HCP.    Families were least comfortable speaking up about hand hygiene and the patients want for more or less aggressive care. |
| ^96^Khan, Spector, Baird,  Ashland, Starmer, Rosenbluth,… on behalf of the Patient and Family Centered I-PASS Study Group  (2018)  (USA) | “To determine whether medical errors, family experience, & communication processes improved after implementation of an intervention to standardize the structure of healthcare provider-family communication on family centered rounds.” (p. 1) | Prospective, multicenter before/after intervention | Patients admitted to study units (3106 admissions, 13171 patient days); 2148 parents or caregivers, 435 nurses, 203 medical students, & 586 residents | The overall rate of medical errors (per 1000 patient days) was unchanged pre-intervention versus post-intervention, but harmful errors (preventable adverse events) decreased by 37.9% post-intervention. Non-preventable adverse events also decreased. (p. 1)  “Top box (e.g., “excellent”) ratings for six of 25 components of family reported experience improved; none worsened. Family centered rounds occurred more frequently…. Family engagement…on rounds improved. Families expressing concerns at the start of rounds…and reading back plans…increased. Trainee teaching and the duration of rounds did not change significantly.” (p. 1) |
| ^97^Lyndon, Wisner, Holschuh, Fagan, & Franck (2017)  (USA) | To describe patients’ perspectives & likelihood of speaking up about safety concerns in the NICU and identify barriers and facilitators to parents speaking up. | Exploratory mixed-methods (parallel convergent) design (interviews, questionnaire, & observations) | 46 parents completed questionnaires, 14 of these were interviewed | Most parents (75%) considered themselves ‘likely’ or ‘very likely’ to speak up in response to lack of hand hygiene. Decisions on whether to speak up were based on knowing the newborn, knowing the team, having a defined pathway for voicing concern, & clinician approachability, availability, friendliness & responsiveness. Despite the severity of the perceived threat, some parents still find it very difficult to speak up about safety concerns. |
| **Author(s) & Year** | **Study Objective(s)** | **Design** | **Sample** | **Relevant Findings** |
| ^98^Dubrovsky, Bishop, Biron, Cunningham-Allard,  DeCivita, Fima, Korah, Marchionni, Proulx, Toman, Tsirgiotis, & Zavalkoff  (2016) (Canada) | The development & implementation of the *We Should Talk c*ampaign at an academic pediatric hospital.  This was a “multimedia campaign was designed to inspire staff, patients and families to effectively communicate to improve patient safety”. (p. 141) | Case study | Montreal Children’s Hospital—McGill University Health Centre | Key factors to success were: being evidence/theory informed, team/skill development, taking the time to define (e.g. problems), organizational alignment, & engaging patients/families (e.g. family advisor on project team).  To track success, key indicators include: (1) “it is easy to speak up if healthcare providers were not observed washing their hands; (2) doctors and nurses were always listening and encouraging families and patients to ask questions; & (3) informing patients/families how to report if they had any concerns about mistakes in their child’s healthcare. To ensure sustainability, the project team successfully integrated these key performance indicators into the hospital’s executive dashboard, as well as collaborated with the hospital’s quality and performance department, to develop and deploy a hospital-wide *We Should Talk* dashboard for frontline teams to track their performance over time.” (p. 144) |
| ^99^**Pinto, Vincent, Darzi, & Davis  (2013)  (UK) | Patient attitudes toward ‘Participate, Inform, Notice, Know (PINK)’ video on patient involvement in safety.  *[Participate: e.g. be involved in decision-making; Inform: e.g. ensure identity confirmed; Notice: e.g. be alert to possible problems & speak up; Know: e.g. if you smoke you put your health at risk; taking some responsibility for your recovery is vital. (p. 30)]* | Qualitative semi-structure interviews | 36 inpatients | Divided opinion on activities such as asking providers about handwashing. Asking about handwashing was seen by some as embarrassing and or that it would negatively impact their relationship with staff. One patient noted that it was part of their (staff) training. Findings suggestive that patients usually ask about treatment. Most indicated they would notify a provider if something was wrong with their care even before the video. Some saw the video as “basic common sense” (p. 31), not adding anything of new value. One patient suggested emphasizing in the video that benefit patients will get by participating. Another suggested having someone watch the video with them so that they could facilitate the messages in the video. |
| ^100^++Rainey, Ehrich, Mackintosh, & Sandall (2013/2015)  (UK) | Examine the experiences and views of patients & their family to determine involvement (speaking up) in promoting safety. | Data set drawn from larger ethnographic study (interviews in home except for one) | 13 patients (long-term health issue experiencing acute illness) & 7 family members | Speaking up influenced by: ability to know/see changes in health; self-monitoring, confidence, trust, culture of healthcare. In hospital, most identified a more passive role related to self-monitoring (e.g. blood sugar levels). Notably, four reported speaking up to prevent medication errors. Relatives identified their advocacy role and being vigilant, expressing worry for those who do not have such support. Some relatives reported speaking up about symptoms, and being reassured when staff took action. Family took a greater role when patients were too ill or cognitively impaired. Many reported trusting the staff who cared for them, and valued long relationships with a trusted hospital/clinician. Family expressed only being comfortable leaving a loved one once they were confident in quality of care. While speaking up about concerns of care limited, on subsequent admissions some had spoken up to request avoiding a previous unit/ward. |
| **Author(s) & Year** | **Study Objective(s)** | **Design** | **Sample** | **Relevant Findings** |
| ^101^Rance,  McCourt, Rayment, Mackintosh, Carter, Watson, & Sandall  (2013)  (UK) | Examines situations in which women felt the need to speak up, and the distress of not being listened to. | Qualitative study (interviews) | 58 postnatal women | 14 women reported raising issues they considered of a safety (urgent) nature. Some described feelings of distress when staff did not respond/listen. The presence of family helped enabling speaking up. |
| ^102^+Entwistle,  McCaughan, Watt, Birks, Hall, Peat, Williams, Wright, for the PIPS (Patient Involvement in Patient Safety) group  (2010)  (UK) | Explore patients’ & family members’ experiences of & views about speaking up about safety issues. | Qualitative study | 71 individual interviews & 12 focus group discussions (experience with certain health conditions or interventions associated with different safety problems or had filed concern with healthcare providers) | Participants had sometimes spoken up about their concerns as they occurred. Speaking up was influenced by: how they assessed its gravity; their concern in light of other’s needs & staff workloads; confidence in their belief about their concern, roles & responsibilities; consequence of speaking up. “Participants expected health professionals and health services to take responsibility for healthcare safety, but generally recognized that there *were* things patients could – and should – do to help keep themselves safe (e.g. telling health professional about current medication and known allergies)” (p. 4). Some found it easier to speak up if they had been encouraged to participate in their care. They wanted to participate without challenging staff or that might be disrespectful. Parents/partners, given their caring responsibility, felt increased obligation to speak up (e.g. is provided of mother speaking up to a new doctor regarding treatment she knew her son needed). |
| ^103^+Davis, Koutantji, & Vincent (2008)  (UK) | Examined: 1) patients’ willingness to question staff about treatment; 2) patients’ willingness to ask factual versus challenging questions; 3) characteristics affecting willingness to ask questions; & 4) impact of doctors’ instructions on willingness to ask. | Cross-sectional study (survey) | 80 surgical patients | Significantly more willing to ask doctors and nurses factual versus challenging questions. Significantly more willing to ask nurses versus doctors challenging questions. Doctor’s instructions increased patient willingness to challenge doctors and nurses. Demographic influences: women, educated patients & employed patients more willing to ask. |
| **Author(s) & Year** | **Study Objective(s)** | **Design** | **Sample** | **Relevant Findings** |
| ^104^Spath  (2007)  (USA) | Guest column – overcoming communication barriers to enable patients in mistake prevention | Not applicable | Not applicable | Authors suggests patients and families have a role to play in healthcare error prevention. She offers that professionals must accept infallibilities, and not feel threatened if patients speak up (e.g. asking about handwashing). She argues that most common barrier in engaging patients in error prevention is patient-practitioner communication. Ineffective communication can limit their ability and willingness to become involved. |
| ^105^Spath  (2003)  (USA) | Opinion piece on the need for providers to give patients a voice in efforts to improve safety. | Not applicable | Not applicable | Author suggests today’s patients are more informed (especially given the Internet) and knowledgeable about health issues/treatment options, but they are more assertive in asking questions. She writes that consumers “share a conviction: *We have the right to participate as much as possible in our health care experience”* (p. 38). She provides 11 actions on how patients can contribute to safety – including: speaking up & asking: if the provider has you confused with someone else; a situation feels unsafe; remind caregivers to mark the site of a procedure. She notes that educating both providers and consumers regarding patient engagement must be done. |

+This study found in Doherty and Stavropoulou (2012) Systematic Review is noted for information but not repeated in final set count.

++This study found in Vaismoradi et al. (2014) Systematic Review is noted for information but not repeated in final set count.

**This study is included in Patient Attitudes table given items of relevance.

Table K

***Patient Behaviours*** *– Patient Handover (n=15)*

| **Author(s) & Year** | **Study Objective(s)** | **Design** | **Sample** | **Relevant Findings** |
| --- | --- | --- | --- | --- |
| **Systematic Reviews & Literature Reviews** | | | | |
| ^106^McCloskey, Furlong, & Hansen (2019)  (Canada) | “The objective of this systematic review was to synthesize the best available evidence on patients’, family members’ and nurses’ experiences with bedside handovers in acute care settings.” (p. 754) | Systematic Review (Qualitative) | 12 papers included | “…five synthesized findings were developed: i) becoming more informed; ii) upholding confidentiality and privacy; iii) varying desire and ability to participate; iv) individualizing patient care; and v) challenges in conducting bedside handovers can be overcome with adaptive practices.” (p. 754) “ Patients also employ various strategies to help make bedside handovers work.” (p. 767) “The link between bedside handover and safety reflects an understanding that patients and family members will take a more active role in their care when they are able to listen to information shared about them, correct inaccuracies, and ask and respond to questions.^39,48,54-56^ A key finding from this study is how the degree to which safety is enhanced varies depending on how bedside hand- overs transpire. When patients listen to what nurses are saying, interject during handovers to correct inaccuracies, pose questions or augment information, safety is enhanced. Conversely, when nurses fail to make a concerted effort to involve patients or to consider unique patient preferences or circumstances, safety may be compromised.” (p. 769) |
| ^107^Tobiano, Bucknall, Sladdin, Whitty, & Chaboyer  (2018)  (Australia) | To clarify the patient’s role in bedside handover, as well as barriers & enhancing strategies. (p. 244). | Systematic mixed-method review | 21 research studies &  25 QI projects  (resulted in 54 articles as some studies/ projects were published in more than 1 article) | Segregated synthesis of research related to patients’ perceptions found contrasting categories: patient-centred handover; & nurse-centred handover. The patients’ role in beside handover is to contribute clinical information related to their care and progress, which may influence safety. There is tension between standardizing handovers and making it predictable for patient participation, while promoting tailored and flexible handovers. (p. 243) “Overwhelmingly, patients wanted to actively participate in handover…viewing it as their right” (p. 251). “Patients had a range of preferences for their level of participation..” (p. 251). “Most frequently patients stated their role was to ask questions during handover…followed by adding information...and preferences….clarifying information…identifying erroneous information…and responding to nurses’ questions”. (p. 251) |
| **Author(s) & Year** | **Study Objective(s)** | **Design** | **Sample** | **Relevant Findings** |
| **Additional Articles of Relevance** | | | | |
| ^108^Callaway, Cunningham, Grover, Steele, McGlynn, & Sribanditongkol  (2018)  (USA) | Identify patient activation scores, patient readmission rates, ad nursing staff satisfaction before and after implementing bedside handoff, the teach-back method, and discharge bundles on an inpatient oncology unit at a large military treatment facility | Quality Improvement Project implementing Plan-Do-Study-Act framework | 49 patients completed the patient activation surveys before implementation and 71 completed the survey after implementation  33 nursing staff completed the satisfaction survey prior to implementation and 32 after implementation | The sample of patients with cancer has high patient activation scores but were not significantly different pre and post implementation. After implementation of three rounds of PDSA cycles, readmission rates decreased from 32% to 25% and staff satisfaction improved. |
| ^109^Malfait, Eeckloo, Lust, Van Biesen, & Van Hecke (2016/2017) (Belgium) | To evaluate the feasibility, appropriateness, meaningfulness, & effectiveness of bedside shift reporting in a minimum of 5 interventions & 5 control wards.  A wide range of variables have been taken into consideration so that the authors can report an in-depth image of the effects of bedside report on nurses & patients, its ability to improve quality of care, communication, & patient participation. | Protocol - controlled, mixed method, longitudinal study design | At least 5 experimental & 5 control wards, with 35 patients per ward. | Not applicable. |
| ^110^Whitty, Spinks, Bucknall, Tobiano, & Chaboyer  (2016/2017)  (Australia) | “This study quantifies and compares the preferences of adult medical patients and nurses for the characteristics of bedside handover”  (p. 743) | Discrete Choice Experiment (DCE) survey | 401 patients;  200 nurses | Handover at the bedside was preferred by nurses and patients. “Being invited to participate, supporting strong two-way communication, having a family member/carer/friend present and having two nurses rather than the nursing team present were most important for patients.” (p. 742) |
| **Author(s) & Year** | **Study Objective(s)** | **Design** | **Sample** | **Relevant Findings** |
| ^111^Becker (2014)  (USA) | Patient perceptions of bedside shift reporting.  (Doctoral Dissertation) | Qualitative case study | 14 inpatients interviewed | Bedside shift report perceived as opportunity to be part of care process. A time to question and clarify information and become informed. Participant reported that he/she liked being part of report so they can hear and possibility even correct staff. |
| ^112^Drach-Zahavy & Shilman (2014/2015)  (Israel) | Quality & scope of patients’ participation in handover process. | Quantitative & qualitative cross-sectional design | 100 handovers – observations; questionnaires; transcripts of handover verbal content (included 100 patients & 100 nurses) | Patients most often initiated communication regarding clarification of ongoing care. Patients view handover viewed as opportunity to get information. An example is given of the patient asking, “what does that mean?” (p. 143). |
| ^113^Jeffs, Beswick, Acott, Simpson, Cardoso, Campbell, & Irwin  (2014)  (Canada) | Explore patients’ experiences and perceptions of bedside nursing handover. | Qualitative study | 45 patient interviews | Themes emerged including: handover was a time to connect with staff; provided opportunity to ask questions, give input on care, and correct information with the nurses. Feeling comfortable to ask and have questions answered was valued. Several described that they were able to identify mistakes, and these could then be addressed. Most patients preferred to be part of them, but some did not see the need (often the latter were long-term-stay patients). Some preferred a more passive role, listening only. |
| ^114^Manias & Watson (2014)  (Australia) | Guest Editorial on patient and family involvement in bedside handover. | Not applicable | Not applicable | Authors note that clinical handover is a major source of communication breakdown. “Health professionals should ask patients and family members about how involved they want to be in bedside handover” (p. 1541). They acknowledge patient/family contribution as vital but as well recognize that some patients may not have an interest in being involved. |
| ^115^Friesen, Herbst, Turner, Speroni, & Robinson (2013)  (USA) | Explore patient perceptions of the **I**ntroduce, **S**tory, **H**istory, **A**ssessment, **P**lan, **E**rror prevention, **D**ialogue (ISHAPED) bedside change-of-shift report process. | Mixed methods - survey questions; Interviews | 93 adult patients & 14 parents who experienced report completed survey; sub-cohort of 16 patients & 6 parents participated in interviews. | Patient noted comfort in hearing what was being communicated, and being able to agree/disagree or add something. Patient comments reflect that patients should not have passive role and can help improve communication. One patient noted they would feel safer if discussion occurs in front of them. |
| **Author(s) & Year** | **Study Objective(s)** | **Design** | **Sample** | **Relevant Findings** |
| ^116^Wildner & Ferri  (2012)  (Italy) | Describe the patient’s bedside handover model. | Action research using  questionnaires (one for patients/family; one for staff) | Hospice inpatients – 15 questionnaires completed by patients/ family members;  Hospice staff – 17 completed by staff | “All patients/family members considered that the bedside handover increased their sense of safety” (p. 219). A significant number of patients did not participate (reasons included patient did not want to as not feeling well) and family members were not always present. |
| ^117^+Flink, Ohlen, Hansagi, Barach, & Olsson (2012b)  (Sweden) | Understanding patients’ perspectives about their participation in the handover process. | Descriptive, qualitative study | 23 patients with chronic diseases were individually interviewed | Handover from primary healthcare to ER: examples of patient participation included showing their medication list from home; providing information on interventions that had helped previously. Patients felt empowered but were sometimes too frail to participate, instead relying on family. Some patients noted they always asked if primary healthcare provider had received information on hospital admission. Others reported being happy with a more passive role. |
| ^106^McCloskey, Furlong, & Hansen (2012)  (Canada) | Systematic Review Protocol: patient, family and nurse experiences with patient presence in patient handover. | Protocol - Studies to include: qualitative & quantitative and discussion/ opinion papers, etc. | Not applicable | See same author team (2019) for completed review.  Protocol source:  McCloskey, R., Furlong, K., & Hansen, L. (2012). Patient, family and nurse experiences with patient presence during hand-off reports within hospitals: A systematic review. JBI Library of Systematic Reviews, 10(28 Supplement). <http://www.joannabriggslibrary.org/jbilibrary/index.php/jbisrir/article/view/300/497> |
| **Author(s) & Year** | **Study Objective(s)** | **Design** | **Sample** | **Relevant Findings** |
| ^118^Flink,  Hesselink, Pijnenborg, Wollersheim, Vernooij-Dassen, Dudzik-Urbaniak, Orrego, Toccafondi, Schoonhoven, Gademan, Johnson, Ohlen, Hansagi, Olsson, Barach, on behalf of the HANDOVER Research Collaborative  (2012a)  (Sweden) | Explore patients’ experiences and perspectives re: handovers between primary care providers and inpatient hospital. | Qualitative secondary analysis | Individual & focus group patient interviews with 90 patients in 5 European countries | “Patients’ participation ranged from being the key actor, to sharing the responsibility with healthcare professional(s), to being passive participants” (p. i89). Active participation necessitated: personal & social characteristics (e.g. being assertive). Some patients had difficulty understanding received information. Family members sometimes took on the role of giving information. Some felt professionals should be fully responsible for the handover. Some did not have any preference for participating or not. |
| ^119^Groene, Orrego, Sunol, Barach, & Groene (2012)  (UK) | Explore the patient’s role in handovers at discharge and the potential additional risks for vulnerable patients. | Qualitative study | Interviews with 12 patients; 6 hospital physicians; 5 hospital nurses; 7 primary care physicians; 4 primary care nurses | Patients role described as “passive conduit function” (p. i70). Patients did not see role as positive – could not understand technical language; could not assess or add to it. Patients believed process to be a responsibility of management/professionals. |
| ^120^McMurray, Chaboyer, Wallis, Johnson, & Gehrke (2011)  (Australia) | Examine patients’ perspectives of participation in bedside shift-to-shift reporting | Descriptive case study | 10 patients who had experienced bedside handover | Patients appreciated being partners in process. Viewed process as opportunity to amend any inaccuracies. Some preferred passive engagement. |

+This study found in Vaismoradi et al. (2014) Systematic Review is noted for information but not repeated in final set count.

Table L

***Patient Behaviours*** *– Diagnostic Imaging/X-ray Imaging (n=2)*

| **Author(s) & Year** | **Study Objective(s)** | **Design** | **Sample** | **Relevant Findings** |
| --- | --- | --- | --- | --- |
| **Systematic Reviews & Literature Reviews** | | | | |
| ^121^McDonald, Bryce, & Graber (2013)  (USA) | Synthesize literature on patient involvement in diagnostic process. | Literature review | Not specifically delineated. | The authors address barriers to patient involvement including health literacy and communication. They provide strategies for patients/families for preventing and detecting diagnostic errors, and questions to ask providers. They acknowledge an imperfect health system, and encourage patients to act as “safety net” (p. ii36) by, for example, keeping and sharing as necessary items such as copies of test results, consultations and discharge summaries. |
| **Additional Articles of Relevance** | | | | |
| ^122^Kim, Jung, Kwon, Noh, & Kim  (2017)  (Korea) | “The purpose of this study was to verify the effectiveness of patient involvement in identifying both patients and the locations before X-ray examinations at orthopedic clinics.” (p.1228) | Pre- and post-test design | Group I: 13617 X-ray orders;  Group II: 12588 X-ray orders | With patient involvement there was a decline in errors in X-ray imaging. There was a significant reduction in errors of left to right (91% reduction), and in information errors (reduction rate 78%). “…the important change in our patient involvement system was the requirement of having multiple medical personnel check the patients using specific closed‐ended questions as well as the involvement of patients themselves playing a more active role in their health care rather than being a passive recipient.” (p. 1229-1230) |

Table M

***Patient Behaviours*** *– Health Literacy & Safety Tips (n=4)*

| **Author(s) & Year** | **Study Objective(s)** | **Design** | **Sample** | **Relevant Findings** |
| --- | --- | --- | --- | --- |
| ^123^Schwappach, Frank, Buschmann, & Babst (2012/2013)  (Switzerland) | Effects of a patient safety advisory on patients’ risk perceptions, perceived behavioural control, performance of safety behaviours, & experience of adverse incidents. | Quasi-experimental intervention study  (Intervention was receiving safety advisory); Questionnaire given at discharge. (Included Yes/No or Agree/Disagree response options to questions such as whether patients asked the provider to mark their surgical site; whether they asked questions about site marking; level of agreement as to whether they know how to protect themselves). | Surgical patients: 218 in control group & 202 in intervention group | Patients in intervention group less likely to feel poorly informed about healthcare error. Perceived behavioural control was lower in control group. Control group underestimated risk for infection compared to intervention group (significant result). Intervention had no effect on performance of safety-related behaviours. And more common behaviours included: informing provider about their medications; and giving provider all information – proactive and challenging behaviours were less frequent. Patients in intervention group less likely to experience unsafe situations. (Notably, healthcare providers were not blinded to the intervention). |
| ^124^*Schwappach, Frank, Koppenberg, Muller, & Wasserfallen (2011)  (Switzerland) | Attitudes toward and experience with safety advisory. | Cross-sectional survey  (All patients admitted to study units received booklet that encourages patient in safety through vigilance, communication & cooperation; Takes 15 minutes to read). (Survey given at discharge) | 1053 patients; 275 healthcare workers at three hospitals | 75% of patients responding reported to have read the entire booklet. 95% of patients agreed that hospital should educate patients how to prevent errors. The survey is based on the Theory of Planned Behaviour, focusing on their intention to engage (& not was they actually did). Overall, patients had a high level of perceived behavioral control & had strong intentions to apply the advice. More than one quarter did notify staff of a potential error. Of 5 recommended actions, the mean scores for acceptance and actual adoption were 6.0, and 4.3 respectively (p<0.001) (actual adoption was highest in asking staff the purpose of a medication & inform staff the medication they usually took). However, 15% of patients strongly disagreed that providers would expect them to apply the recommendations. Fear of negative staff reactions was reported as the main barrier to applying the advised actions. (Advisory created with input from patient/relatives focus groups.). |
| ^125^Weingart,  Morway, Brouillard, Cleary, Eng, Saadeh, Seger, Bates, & Leape  (2009)  (USA) | Investigators and consumers examined patient safety recommendations for quality, benefit and likely patient adherence. | Descriptive, exploratory study | 9 investigators and 22 relatives of the investigators who were also mothers examined advisories and scored them. | 160 distinct patient safety recommendations were identified. Mothers rated potential benefit higher than likely adherence. Mothers’ scores were higher than investigators. Notably, 8 of the mothers’ highly rated items were among the 25 least frequently cited recommendations. |
| **Author(s) & Year** | **Study Objective(s)** | **Design** | **Sample** | **Relevant Findings** |
| ^126^AHC Media [currently known as Relias Media]  (2003)  (USA) | Discussion paper of one organization (University of Washington Medical Center) in developing a tool for patients to understand their role in partnering to create a safe care experience. | Not applicable | Not applicable | After staff committee created first draft of the tool on safety tips, 8 consumers were asked to evaluate it. In the dialogue with consumers, it became evident everyone had a different perspective of patient safety. The site lead suggests it was not until they read the tool that they understood what patient safety was. Consumers identified that certain instructions they were not comfortable doing (e.g. asking about provider handwashing). The developers changed the instructions to have them ask everyone about handwashing, believing they would then become used to the practice. |

*This study included in Patient Attitudes table given items of relevance.

Table N

***Patient Behaviours*** *– Technology (n=3)*

| **Author(s) & Year** | **Study Objective(s)** | **Design** | **Sample** | **Relevant Findings** |
| --- | --- | --- | --- | --- |
| **Systematic Reviews & Literature Reviews** | | | | |
| ^127^Dendere, Slade, Burton-Jones, Sullivan, Staib & Janda  (2019)  (Australia) | To review literature about patient portals connected to an electronic medical record (EMR) in inpatient settings, their role in patient engagement, & their impact on health care delivery to identify factors & best practices for successful implementation of this technology (p. 1) | Systematic review | 58 articles included in the review (19 articles were rated as ‘high’ to ‘very high’ quality) | Patients’ privacy concerns & lack of encouragement from providers were among portal adoption barriers, while information access & patient-provider communication were among facilitators. Several methods were used to train portal users with varying success.  Sociodemographic characteristics & medical conditions of patients were predictors of portal use; some patients wanted unlimited access to their EMRs, personalized health education, and nonclinical information; and patients were keen to use portals for communicating with their health care teams.  In some but not all studies they found that patient portals improved patient engagement; patients perceived some portal functions as inadequate but others as useful; patients and staff thought portals may improve patient care but could cause anxiety in some patients; and portals improved patient safety, adherence to medications, & patient-provider communication but had no impact on objective health outcomes. (p. 1-2) |
| **Additional Articles of Relevance** | | | | |
| ^128^Duckworth, Leung, Fuller, Espares, Couture, Chang, Businger, Collins, Dalal, Fladger, Schnipper, Schnock, Bates, & Dykes  (2017)  (USA) | “The specific aims of this study are to: (a) Describe nursing staff, patient, and informal caregiver perceptions of the personalized safety plan screensaver, including potential benefits, unintended consequences on decision making and workflow, and suggestions for improvement. (b) Identify barriers to use and strategies to overcome these barriers in future iterations of the personalized patient safety screensaver.” (p. 18) | Descriptive study using qualitative semi-structured interviews | 6 patients & 5 informal caregivers (plus formal care providers) in  Phase 1;  6 patients & 2 informal caregivers (plus formal care providers) in  Phase 2 | Themes elicited by end users regarding the safety plan include appreciation of the clinical decision support provided, recognition of workflow efficiency, enthusiasm for the safety-centric content, and recommendations for content and aesthetics. Barriers included lack of awareness, familiarity, agreement, self-efficacy, and outcomes expected.  The author team developed a personalized safety plan screensaver to display the core set of information needed by the care team, including patients and informal caregivers, to engage in safe, efficient, and effective care at the bedside.  “Patients and informal caregivers placed complete faith in the information on the screensavers, not questioning whether the logic or documentation was accurate.” (p. 20) |
| **Author(s) & Year** | **Study Objective(s)** | **Design** | **Sample** | **Relevant Findings** |
| ^129^Oermann, Hamilton, & Shook  (2003)  (USA) | To assess the value of using the Web to teach older adults about their role in preventing medical errors. | Pretest – post-test design  (educational intervention using 5 Web sites that gave information at patient’s role in safety. Post-test completed after intervention). | 26 seniors  (Community centre that served senior citizens) | Of the testing, which included 24 true/false questions, the mean pretest score was 75%, and the mean post-test score was 92% - suggestive that seniors had knowledge of their role in error prevention prior to the educational session, but that learning was enhanced. |

Table O

***Patient Behaviours*** *– Rapid Response Team Activation (n=2)*

| **Author(s) & Year** | **Study Objective(s)** | **Design** | **Sample** | **Relevant Findings** |
| --- | --- | --- | --- | --- |
| **Systematic Reviews & Literature Reviews** | | | | |
| ^130^Gardner & Hampton (2014)  (USA) | Document evidence related to the effect of rapid response teams activated by patients/family on mortality and cardiac arrest on non-intensive care units. | Systematic Review Protocol | Not available | Completed review was not obtained. [Note: Of 15 references provided in the Reference list, only one publication (commentary) is linked specifically with this topic, the remaining references address patient engagement in safety generally, patient-family centred literature, or rapid response teams generally). |
| **Additional Articles of Relevance** | | | | |
| ^131^Guinane, Hutchinson, & Bucknall (2018) (Australia) | To investigate the experiences of patients who had a period of deterioration & received medical emergency team review & to garner patient views & attitudes about the potential use of a patient and family activated escalation system (PFAES). | Qualitative exploratory descriptive design | Purposive sampling for 33 patients who required medical emergency team intervention;  data collected from one private and one public hospital | Patients were comfortable communicating concerns to clinicians but felt they would not “override” their clinician and activate the escalation system   - Participants feared being seen as a burden, as they are overreacting, or as they are not qualified to activate an emergency system - Participants stated that they notified their health care providers at the onset of symptoms and it was up to the HCP to interpret the symptoms and their potential for clinical deterioration - There is trust that the HCP is competent to monitor and manage the patients’ clinical status - Patient lack of medical knowledge creates apprehension in the idea of overriding a nurses’ decision making by using the patient and family activated emergency system   A small portion of public patient participants believed PFAES would be beneficial and a way to fast tract medical care. |

Table P

***Patient Behaviours*** *(n=123) – Ambulatory and Primary Care (n=4)*

| **Author(s) & Year** | **Study Objective(s)** | **Design** | **Sample** | **Relevant Findings** |
| --- | --- | --- | --- | --- |
| **Systematic Reviews & Literature Reviews** | | | | |
| ^132^Trier, Valderas, Wensing, Martin, & Egebart (2015)  (Denmark) | Involving patients in patient safety programmes: A scoping review and consensus procedure by the LINNEAUS collaboration on patient safety in primary care. | Scoping Review | 13 articles & 5 reports – focus on primary care | Weak evidence in support of the effectiveness of patient involvement in patient safety. Most strategies are about speaking up. Barriers included patient characteristics such as lack of education. Patient experience less difficulty participating with a GP than in hospitals. It is easier for patients to complete task not requiring medical or non-confrontational actions. |
| ^133^Martin & Larsen (2012)  (Denmark) | *Patient involvement in Patient Safety: A literature review about European primary care.* | Literature Review | Not applicable. | The Danish Institute for Health Services Research for the Danish Society for Patient Safety and the LINNEAUS EURO-PC project. Examined best practice of patient involvement in patient safety in primary care. They note the literature is “very heterogeneous” (p. 5). They suggest this is an understudied topic. (54 page document) |
| **Additional Articles of Relevance** | | | | |
| ^134^Roter, Wolff, Wu, & Hannawa  (2017)  (USA) | 1) Hypothesise pathways through which an empowered patient-family partnership may effective advance healthcare safety and quality in ambulatory care settings and home.  2) Describe key elements and lessons learned from successful communication intervention designed to empower patients and families to effectively work together with clinicians during ambulatory care. | View point commentary | Family defined as spouses, partners, adult children, relatives, and friends | It is hypothesized that:   - family members contribute to safer med use and fewer preventable adverse ambulatory drugs events by minimising drug miscommunication and misunderstanding in visits when medication is prescribed by recording and clarifying medication-related instructions, being vigilant for side effects, reporting past adverse events, monitoring for side effects at home and taking actions when side effects occur - family contributes to effectiveness of care by facilitating medication related communication during medical visits when the medication is prescribed, giving information to the physician about the patient, and through the provision of social support - family enhances care by providing a history, current medical information, recording physician recommendations, and facilitating patient follow-ups - families contribute to health equity by advocating on patient’s behalf, providing encouragement, facilitating active patient engagement - families contribute to patient-centered care by facilitating clear, informative, and supportive medical visit communication, encouraging patient expression of preferences, and aiding patient in treatment decisions. |
| **Author(s) & Year** | **Study Objective(s)** | **Design** | **Sample** | **Relevant Findings** |
| ^135^Kingston-Riechers,  Ospina, Jonsson, Childs, McLeod, & Maxted  (2010)  (Canada) | *Patient Safety in Primary Care.* | Opinion/  white paper | Not applicable. | Systematic review; key informant interviews and roundtable event (approximately 50 stakeholders including patient representatives). System for reliably documenting adverse events in primary care lacking. Roundtable participants supported findings of literature review and key informants. They agree that unique to primary care is the opportunity for providers to build long-term open & significant relationships with patients over time, and this has implication for safety. |

Table Q

***Patient Behaviours*** *– Newborn Fall Prevention (n=1)*

| **Author(s) & Year** | **Study Objective(s)** | **Design** | **Sample** | **Relevant Findings** |
| --- | --- | --- | --- | --- |
| ^136^Lipke, Gilbert, Shimer, Consenstein, Aris, Ponto, Lafaver, & Kowal  (2018)  (US) | To develop a newborn infant safety bundle & evaluate its efficacy in helping reduce unsafe sleep situations while also preventing newborn falls. (p. 32) | Observational, descriptive safety study  “The bundle included: (a) a parent safety agreement; (b) education, teach-back, and role modeling of safe sleep practices; and (c) implementation of a reporting and debriefing system for infant falls.” (p. 32) | “Preprogram (baseline) data were collected in March 2015 prior to beginning the study. Data were collected during a randomly chosen month per quarter (June 2015–June 2016) using an evaluation instrument developed by the hospital’s clinical taskforce based on unsafe sleep situations reported in the literature.” (p. 35) | “Fourteen percent (n = 23) of babies born at the hospital in March 2015 were found to be exposed to risk-to-fall situations; over half of their mothers were found asleep and still holding the baby. Following bundle implementation, identified unsafe sleep situations during June 2015 to June 2016 have trended down with no reports of an infant fall through May 2017.” (p. 32)  “We found some parents who refused to sign the safety agreement because they felt that by signing the form, an infant fall would become their fault. We identified this as an opportunity for nursing education, as it was reported some nurses were simply presenting the form to parents to sign without offering the education. Parents needed to understand that the safety agreement was intended to be a partnership between the parent and staff to help keep the newborn safe.” (p. 36) |

References

1. Berger Z, Flickinger TE, Pfoh E, Martinez KA, Dy SM. Promoting engagement by patients and families to reduce adverse events in acute care settings: a systematic review. *BMJ quality & safety.* 2014;23(7):548-555.

2. Vaismoradi M, Jordan S, Kangasniemi M. Patient participation in patient safety and nursing input - a systematic review. *Journal of Clinical Nursing.* 2015;24(5-6):627-639.

3. Doherty C, Stavropoulou C. Patients' willingness and ability to participate actively in the reduction of clinical errors: a systematic literature review. *Social Science & Medicine.* 2012;75(2):257-263.

4. Hall J, Peat M, Birks Y, et al. Effectiveness of interventions designed to promote patient involvement to enhance safety: a systematic review. *Quality & safety in health care.* 2010;19(5):e10.

5. Schwappach DLB. Review: engaging patients as vigilant partners in safety: a systematic review. *Medical care research and review: MCRR.* 2010;67(2):119-148.

6. Scobie AC, Persaud DD. Patient engagement in patient safety: Barriers and facilitators. *Patient Saf Qual Healthc.* 2010;7:42-47.

7. Davis RE, Jacklin R, Sevdalis N, Vincent CA. Patient involvement in patient safety: what factors influence patient participation and engagement? *Health expectations : an international journal of public participation in health care and health policy.* 2007;10(3):259-267.

8. Liberatore K. Speaking Up for Safety-It's Not Simple. *Pennsylvania Patient Safety Advisory.* 2018;15(3):37-47.

9. Ringdal M, Chaboyer W, Ulin K, Bucknall T, Oxelmark L. Patient preferences for participation in patient care and safety activities in hospitals. *BMC Nursing.* 2017;16:1-8.

10. Thomas A, Silver SA, Rathe A, et al. Feasibility of a hemodialysis safety checklist for nurses and patients: A quality improvement study. *Clinical Kidney Journal.* 2016;9(3):335-342.

11. Tobiano G, Bucknall T, Marshall A, Guinane J, Chaboyer W. Patients' perceptions of participation in nursing care on medical wards. *Scandinavian Journal of Caring Sciences.* 2016;30(2):260-270.

12. Spruce L. Back to Basics: Patient and Family Engagement. *AORN journal.* 2015;102(1):34-39.

13. Sahlstrom M, Partanen P, Turunen H. Safety as experienced by patients themselves: a Finnish survey of the most recent period of care. *Research in Nursing & Health.* 2014;37(3):194-203.

14. Gillespie BM, Evidence NCfR. Partnering with patients to provide safe health care. *The Queensland nurse.* 2013;32(4):39.

15. Hor SY, Godbold N, Collier A, Iedema R. Finding the patient in patient safety. *Health: an Interdisciplinary Journal for the Social Study of Health, Illness & Medicine.* 2013;17(6):567-583.

16. World Health Organization - Regional Office for Europe. *Exploring patient participation in reducing health-care-related safety risks.*Copenhagen, Denmark. 2013.

17. Birks Y, Hall J, McCaughan D, Peat M, Watt I. Promoting patient involvement in safety initiatives. *Nursing management (Harrow, London, England: 1994).* 2011;18(1):16-20.

18. Weingart SN, Zhu J, Chiappetta L, et al. Hospitalized patients' participation and its impact on quality of care and patient safety. *International journal for quality in health care : journal of the International Society for Quality in Health Care.* 2011;23(3):269-277.

19. Clark PR. *An Emergency Department Patient's Perception of Safety: A Dissertation*. San Antonio:TX, The University of Texas Health Science Center at San Antonio; 2010.

20. Davis R. *An investigation of patients' willingness and ability to participate in safety-related aspects of their healthcare management.* London, England, Imperial College London; 2009.

21. Jorm CM, Dunbar N, Sudano L, Travaglia JF. Should patient safety be more patient centred? *Australian health review : a publication of the Australian Hospital Association.* 2009;33(3):390-399.

22. Marella WM, Finley E, Thomas AD, Clarke JR. Health care consumers' inclination to engage in selected patient safety practices: a survey of adults in Pennsylvania. *Journal of Patient safety.* 2007;3(4):184-189.

23. Entwistle VA. Differing perspectives on patient involvement in patient safety. *Quality & safety in health care.* 2007;16(2):82-83.

24. Lyons M. Should patients have a role in patient safety? A safety engineering view. *Quality & safety in health care.* 2007;16(2):140-142.

25. Coulter A. Patient safety: what role can patients play? *Health expectations : an international journal of public participation in health care and health policy.* 2006;9(3):205-206.

26. Unruh KT, Pratt W. Patients as actors: the patient's role in detecting, preventing, and recovering from medical errors. *International journal of medical informatics.* 2007;76 Suppl 1:S236-244.

27. Entwistle V. Nursing shortages and patient safety problems in hospital care: is clinical monitoring by families part of the solution? *Health expectations : an international journal of public participation in health care and health policy.* 2004;7(1):1-5.

28. Vincent CA, Coulter A. Patient safety: what about the patient? *Quality & safety in health care.* 2002;11(1):76-80.

29. Lawton R, O'Hara JK, Sheard L, et al. Can patient involvement improve patient safety? A cluster randomised control trial of the Patient Reporting and Action for a Safe Environment (PRASE) intervention. *BMJ Quality & Safety.* 2017;26(8):622-631.

30. Sheard L, O'Hara J, Armitage G, et al. Evaluating the PRASE patient safety intervention - a multi-centre, cluster trial with a qualitative process evaluation: study protocol for a randomised controlled trial. *Trials [Electronic Resource].* 2014;15:420.

31. Davis RE, Sevdalis N, Neale G, Massey R, Vincent CA. Hospital patients' reports of medical errors and undesirable events in their health care. *Journal of evaluation in clinical practice.* 2013;19(5):875-881.

32. Hasegawa T, Fujita S, Seto K, Kitazawa T, Matsumoto K. Patients' identification and reporting of unsafe events at six hospitals in Japan. *Joint Commission Journal on Quality & Patient Safety.* 2011;37(11):502-508.

33. Wasson JH, MacKenzie TA, Hall M. Patients use an internet technology to report when things go wrong. *Qual Saf Health Care.* 2007;16(3):213-215.

34. Ball LK, George CA, Duval L, Hedrick NNF. Reducing blood stream infection in patients on hemodialysis: Incorporating patient engagement into a quality improvement activity. *Hemodialysis International.* 2016;20(Supplement 1):S7-S11.

35. Wyer M, Jackson D, Iedema R, et al. Involving patients in understanding hospital infection control using visual methods. *Journal of clinical nursing.* 2015;24(11-12):1718-1729.

36. Seale H, Chughtai AA, Kaur R, et al. Ask, speak up, and be proactive: Empowering patient infection control to prevent health care-acquired infections. *American journal of infection control.* 2015;43(5):447-453.

37. Davis RE, Vincent CA, Murphy MF. Blood transfusion safety: the potential role of the patient. *Transfusion medicine reviews.* 2011;25(1):12-23.

38. World Health Organization. Patients for Patient Safety – Partnerships for Safer Health Care. In. Geneva, Switzerland: World Health Organization; 2013.

39. Ocloo JE. Harmed patients gaining voice: challenging dominant perspectives in the construction of medical harm and patient safety reforms. *Social science & medicine (1982).* 2010;71(3):510-516.

40. Kovacs Burns K. Canadian patient safety champions: collaborating on improving patient safety. *Healthcare quarterly (Toronto, Ont).* 2008;11(3 Spec No.):95-100.

41. Anderson KJ, Bradford NK, Clark JE. Through Their Eyes: Parental Perceptions on Hospital Admissions for Febrile Neutropenia in Children With Cancer. *Journal of Pediatric Oncology Nursing.* 2018;35(5):342-352.

42. Oyesanya TO, Bowers B. Managing Visitors During the Hospital Stay: The Experience of Family Caregivers of Patients With Traumatic Brain Injury. *Journal of Family Nursing.* 2017;23(2):273-298.

43. Rosenberg RE, Rosenfeld P, Williams E, et al. Parents' Perspectives on "Keeping Their Children Safe" in the Hospital. *Journal of nursing care quality.* 2016;31(4):318-326.

44. Sandlin-Leming D. Pediatric patient safety: educating parents. *Journal of perianesthesia nursing : official journal of the American Society of PeriAnesthesia Nurses.* 2010;25(2):116-118.

45. Tarini BA, Lozano P, Christakis DA. Afraid in the hospital: parental concern for errors during a child's hospitalization. *Journal of hospital medicine.* 2009;4(9):521-527.

46. Clarke JN, Fletcher PC. Parents as advocates: stories of surplus suffering when a child is diagnosed and treated for cancer. *Social work in health care.* 2004;39(1-2):107-127.

47. Hurst I. Vigilant watching over: mothers' actions to safeguard their premature babies in the newborn intensive care nursery. *The Journal of perinatal & neonatal nursing.* 2001;15(3):39-57.

48. Alzyood M, Jackson D, Brooke J, Aveyard H. An integrative review exploring the perceptions of patients and healthcare professionals towards patient involvement in promoting hand hygiene compliance in the hospital setting. *Journal of Clinical Nursing (John Wiley & Sons, Inc).* 2018;27(7-8):1329-1345.

49. Butenko S, Lockwood C, McArthur A. Patient experiences of partnering with healthcare professionals for hand hygiene compliance: a systematic review. *JBI Database of Systematic Reviews & Implementation Reports.* 2017;15(6):1645-1670.

50. Butenko S, Lockwood C, McArthur A. The patient/consumer experience of partnering with health care professionals with hand hygiene compliance: a systematic review protocol. *JBI database of systematic reviews and implementation reports.* 2015;13(4):127-140.

51. Davis R, Parand A, Pinto A, Buetow S. Systematic review of the effectiveness of strategies to encourage patients to remind healthcare professionals about their hand hygiene. *The Journal of hospital infection.* 2015;89(3):141-162.

52. Li Y, Liu Y, Zeng L, Chen C, Mo D, Yuan S. Knowledge and practice of hand hygiene among hospitalised patients in a tertiary general hospital in China and their attitudes: a cross-sectional survey. *BMJ Open.* 2019;9(6):e027736.

53. Sande-Meijide M, Lorenzo-González M, Mori-Gamarra F, et al. Perceptions and attitudes of patients and health care workers toward patient empowerment in promoting hand hygiene. *American Journal of Infection Control.* 2019;47(1):45-50.

54. Knighton SC, Dolansky M, Donskey C, Warner C, Rai H, Higgins PA. Use of a verbal electronic audio reminder with a patient hand hygiene bundle to increase independent patient hand hygiene practices of older adults in an acute care setting. *American Journal of Infection Control.* 2018;46(6):610-616.

55. Cheng VCC, Wong S-C, Wong IWY, et al. The challenge of patient empowerment in hand hygiene promotion in health care facilities in Hong Kong. *American Journal of Infection Control.* 2017;45(5):562-565.

56. Desai C, Rezmovitz J, Manson J, Callery S, Vearncombe M. Engaging patients as observers in monitoring hand hygiene compliance in ambulatory care. *Canadian Journal of Infection Control.* 2017;32(3):150-153.

57. Doyle GA, Xiang J, Zaman H, et al. Patient Attitudes and Participation in Hand Co-Washing in an Outpatient Clinic Before and After a Prompt. *Annals of Family Medicine.* 2017;15(2):155-157.

58. Haverstick S, Goodrich C, Freeman R, James S, Kullar R, Ahrens M. Patients' Hand Washing and Reducing Hospital-Acquired Infection. *Critical care nurse.* 2017;37(3):e1-e8.

59. Lastinger A, Gomez K, Manegold E, Khakoo R. Use of a patient empowerment tool for hand hygiene. *American Journal of Infection Control.* 2017;45(8):824-829.

60. Ong AYJ, Tan J, Yeo HL, Goh ML. Patient-centred hand hygiene information in orthopaedics units: an evidence-based implementation project. *International Journal of Evidence-Based Healthcare.* 2017;15(1):22-29.

61. Pokrywka M, Buraczewski M, Frank D, et al. Can improving patient hand hygiene impact Clostridium difficile infection events at an academic medical center? *American Journal of Infection Control.* 2017;45(9):959-963.

62. Rai H, Knighton S, Zabarsky TF, Donskey CJ. A randomized trial to determine the impact of a 5 moments for patient hand hygiene educational intervention on patient hand hygiene. *American Journal of Infection Control.* 2017;45(5):551-553.

63. Sunkesula VCK, Kundrapu S, Knighton S, Cadnum JL, Donskey CJ. A Randomized Trial to Determine the Impact of an Educational Patient Hand-Hygiene Intervention on Contamination of Hospitalized Patient’s Hands with Healthcare-Associated Pathogens. *Infection Control & Hospital Epidemiology.* 2017;38(5):595-597.

64. Caine LZ, Pinkham AM, Noble JT. Be seen and heard being clean: A novel patient-centered approach to hand hygiene. *American Journal of Infection Control.* 2016;44(7):e103-e106.

65. Cheng VCC, Tai JWM, Li WS, et al. Implementation of directly observed patient hand hygiene for hospitalized patients by hand hygiene ambassadors in Hong Kong. *American Journal of Infection Control.* 2016;44(6):621-624.

66. Stewardson AJ, Sax H, Gayet-Ageron A, et al. Enhanced performance feedback and patient participation to improve hand hygiene compliance of health-care workers in the setting of established multimodal promotion: a single-centre, cluster randomised controlled trial. *Lancet Infectious Diseases.* 2016;16(12):1345-1355.

67. von Lengerke T, Kroning B, Lange K. Patients' intention to speak up for health care providers' hand hygiene in inpatient diabetic foot wound treatment: a cross-sectional survey in diabetes outpatient centres in Lower Saxony, Germany. *Psychology, health & medicine.* 2017;22(10):1137-1148.

68. Busby SR, Kennedy B, Davis SC, Thompson HA, Jones JW. Assessing patient awareness of proper hand hygiene. *Nursing.* 2015;45(5):27-30.

69. Kim M-K, Nam EY, Na SH, et al. Discrepancy in perceptions regarding patient participation in hand hygiene between patients and health care workers. *American journal of infection control.* 2015;43(5):510-515.

70. McGuckin M, Govednik J. Patient empowerment begins with knowledge: consumer perceptions and knowledge sources for hand hygiene compliance rates. *American journal of infection control.* 2014;42(10):1106-1108.

71. Le-Abuyen S, Ng J, Kim S, et al. Patient-as-observer approach: an alternative method for hand hygiene auditing in an ambulatory care setting. *American journal of infection control.* 2014;42(4):439-442.

72. Pan S-C, Tien K-L, Hung IC, et al. Patient empowerment in a hand hygiene program: differing points of view between patients/family members and health care workers in Asian culture. *American journal of infection control.* 2013;41(11):979-983.

73. Rogers S. Engaging patients and family members in better hand hygiene practices: a teaching hospital's challenge. *Healthcare quarterly (Toronto, Ont).* 2013;16(4):27-31.

74. Reid N, Moghaddas J, Loftus M, et al. Can we expect patients to question health care workers' hand hygiene compliance? *Infection control and hospital epidemiology.* 2012;33(5):531-532.

75. Ciofi degli Atti ML, Tozzi AE, Ciliento G, Pomponi M, Rinaldi S, Raponi M. Healthcare workers' and parents' perceptions of measures for improving adherence to hand-hygiene. *BMC public health.* 2011;11:466.

76. McGuckin M, Waterman R, Shubin A. Consumer attitudes about health care-acquired infections and hand hygiene. *American journal of medical quality : the official journal of the American College of Medical Quality.* 2006;21(5):342-346.

77. Tobiano G, Chaboyer W, Teasdale T, Raleigh R, Manias E. Patient engagement in admission and discharge medication communication: A systematic mixed studies review. *Int J Nurs Stud.* 2019;95:87-102.

78. Kim JM, Suarez-Cuervo C, Berger Z, et al. Evaluation of Patient and Family Engagement Strategies to Improve Medication Safety. *Patient.* 2018;11(2):193-206.

79. Wang BH, Zhang JJ, Zhang J, Zhu Q, Yan QY. The development and psychometric testing of Inpatients' Involvement in Medication Safety Scale (IIMSS). *J Nurs Manag.* 2019;27(8):1648-1654.

80. Phipps DL, Giles S, Lewis PJ, et al. Mindful organizing in patients' contributions to primary care medication safety. *Health Expectations.* 2018;21(6):964-972.

81. Prey JE, Polubriaginof F, Grossman LV, et al. Engaging hospital patients in the medication reconciliation process using tablet computers. *Journal of the American Medical Informatics Association.* 2018;25(11):1460-1469.

82. Schöpf AC, von Hirschhausen M, Farin E, Maun A. Elderly patients' and GPs' perspectives of patient–GP communication concerning polypharmacy: a qualitative interview study. *Primary Health Care Research & Development (Cambridge University Press / UK).* 2018;19(4):355-364.

83. Garfield S, Jheeta S, Husson F, et al. The Role of Hospital Inpatients in Supporting Medication Safety: A Qualitative Study. *PloS one.* 2016;11(4):e0153721.

84. Heyworth L, Paquin AM, Clark J, et al. Engaging patients in medication reconciliation via a patient portal following hospital discharge. *Journal of the American Medical Informatics Association : JAMIA.* 2014;21(e1):e157-162.

85. Macdonald MT, Heilemann MV, MacKinnon NJ, et al. Confirming delivery: understanding the role of the hospitalized patient in medication administration safety. *Qualitative Health Research.* 2014;24(4):536-550.

86. McTier L, Botti M, Duke M. Patient participation in medication safety during an acute care admission. *Health expectations : an international journal of public participation in health care and health policy.* 2015;18(5):1744-1756.

87. Schwappach DLB, Wernli M. Medication errors in chemotherapy: incidence, types and involvement of patients in prevention. A review of the literature. *European journal of cancer care.* 2010;19(3):285-292.

88. Myhre TA. *Medication safety practices: A patient's perspective*. Lethbridge, Alberta: School of Health Sciences, University of Lethbridge; 2007.

89. Wright J, Emerson A, Stephens M, Lennan E. Hospital inpatient self-administration of medicine programmes: a critical literature review. *Pharmacy world & science : PWS.* 2006;28(3):140-151.

90. See LC, Chang YH, Chuang KL, et al. Animation program used to encourage patients or family members to take an active role for eliminating wrong-site, wrong-person, wrong-procedure surgeries: preliminary evaluation. *Int J Surg.* 2011;9(3):241-247.

91. Boyd M, Holroyd B. Patient involvement in preoperative marking. *British journal of hospital medicine (London, England : 2005).* 2011;72(9):535.

92. Bergal LM, Schwarzkopf R, Walsh M, Tejwani NC. Patient participation in surgical site marking: can this be an additional tool to help avoid wrong-site surgery? *Journal of patient safety.* 2010;6(4):221-225.

93. Jeffrey A, Curry P. Patient participation in surgical pause. *Anaesthesia.* 2010;65(2):217.

94. DiGiovanni CW, Kang L, Manuel J. Patient compliance in avoiding wrong-site surgery. *The Journal of bone and joint surgery American volume.* 2003;85-A(5):815-819.

95. Bell SK, Roche SD, Mueller A, et al. Speaking up about care concerns in the ICU: patient and family experiences, attitudes and perceived barriers. *BMJ Quality & Safety.* 2018;27(11):928-936.

96. Khan A, Spector ND, Baird JD, et al. Patient safety after implementation of a coproduced family centered communication programme: multicenter before and after intervention study. *BMJ (Clinical research ed).* 2018;363:k4764.

97. Lyndon A, Wisner K, Holschuh C, Fagan KM, Franck LS. Parents' Perspectives on Navigating the Work of Speaking Up in the NICU. *JOGNN: Journal of Obstetric, Gynecologic & Neonatal Nursing.* 2017;46(5):716-726.

98. Dubrovsky AS, Bishop A, Biron A, et al. "We should talk" - Moving knowledge into action by learning to engage patients, families, and healthcare staff to communicate for patient safety. *Healthcare Management Forum.* 2016;29(4):141-145.

99. Pinto A, Vincent C, Darzi A, Davis R. A qualitative exploration of patients' attitudes towards the 'Participate Inform Notice Know' (PINK) patient safety video. *International Journal for Quality in Health Care.* 2013;25(1):29-34.

100. Rainey H, Ehrich K, Mackintosh N, Sandall J. The role of patients and their relatives in 'speaking up' about their own safety - a qualitative study of acute illness. *Health Expectations.* 2015;18(3):392-405.

101. Rance S, McCourt C, Rayment J, et al. Women's safety alerts in maternity care: is speaking up enough? *BMJ quality & safety.* 2013;22(4):348-355.

102. Entwistle VA, McCaughan D, Watt IS, et al. Speaking up about safety concerns: multi-setting qualitative study of patients' views and experiences. *Quality & safety in health care.* 2010;19(6):e33.

103. Davis RE, Koutantji M, Vincent CA. How willing are patients to question healthcare staff on issues related to the quality and safety of their healthcare? An exploratory study. *Quality & safety in health care.* 2008;17(2):90-96.

104. Spath PL. Guest Column:  Involve patients in mistake prevention:  How to overcome communication barriers. *Hospital Case Management.* 2007;15(6):92-94.

105. Spath PL. "Can you hear me now?" Providers must give patients a voice in efforts to reduce medical errors. *Hospitals & health networks.* 2003;77(12):36-32.

106. McCloskey R, Furlong K, Hansen L. Patient, family and nurse experiences with patient presence during handovers in acute care hospital settings: A systematic review of qualitative evidence. *JBI Database of Systemativ Reviews and Implementation Reports.* 2019;17(5):754-792.

107. Tobiano G, Bucknall T, Sladdin I, Whitty JA, Chaboyer W. Patient participation in nursing bedside handover: A systematic mixed-methods review. *International Journal of Nursing Studies.* 2018;77:243-258.

108. Callaway C, Cunningham C, Grover S, Steele KR, McGlynn A, Sribanditmongkol V. Patient Handoff Processes: Implementation and effects of bedside handoffs, the teach-back method, and discharge bundles on an inpatient oncology unit. *Clinical Journal of Oncology Nursing.* 2018;22(4):421-428.

109. Malfait S, Eeckloo K, Lust E, Van Biesen W, Van Hecke A. Feasibility, appropriateness, meaningfulness and effectiveness of patient participation at bedside shift reporting: mixed-method research protocol. *Journal of Advanced Nursing (John Wiley & Sons, Inc).* 2017;73(2):482-494.

110. Whitty JA, Spinks J, Bucknall T, Tobiano G, Chaboyer W. Patient and nurse preferences for implementation of bedside handover: Do they agree? Findings from a discrete choice experiment. *Health Expectations.* 2017;20(4):742-750.

111. Becker CA. *Patient perceptions of bedside shift report: A qualitative case study*. Phoenix, Arizona, University of Phoenix; 2014.

112. Drach-Zahavy A, Shilman O. Patients' participation during a nursing handover: the role of handover characteristics and patients' personal traits. *Journal of advanced nursing.* 2015;71(1):136-147.

113. Jeffs L, Beswick S, Acott A, et al. Patients' views on bedside nursing handover: creating a space to connect. *Journal of nursing care quality.* 2014;29(2):149-154.

114. Manias E, Watson B. Moving from rhetoric to reality: patient and family involvement in bedside handover. *International journal of nursing studies.* 2014;51(12):1539-1541.

115. Friesen MA, Herbst A, Turner JW, Speroni KG, Robinson J. Developing a patient-centered ISHAPED handoff with patient/family and parent advisory councils. *Journal of nursing care quality.* 2013;28(3):208-216.

116. Wildner J, Ferri P. Patient participation in change-of-shift procedures: the implementation of the bedside handover for the improvement of nursing quality in an Italian hospice. *Journal of Hospice & Palliative Nursing.* 2012;14(3):216-224.

117. Flink M, Ohlen G, Hansagi H, Barach P, Olsson M. Beliefs and experiences can influence patient participation in handover between primary and secondary care--a qualitative study of patient perspectives. *BMJ quality & safety.* 2012;21 Suppl 1:i76-83.

118. Flink M, Hesselink G, Pijnenborg L, et al. The key actor: a qualitative study of patient participation in the handover process in Europe. *BMJ Quality & Safety.* 2012;21 Suppl 1:i89-96.

119. Groene RO, Orrego C, Sunol R, Barach P, Groene O. "It's like two worlds apart": an analysis of vulnerable patient handover practices at discharge from hospital. *BMJ quality & safety.* 2012;21 Suppl 1:i67-75.

120. McMurray A, Chaboyer W, Wallis M, Johnson J, Gehrke T. Patients' perspectives of bedside nursing handover. *Collegian (Royal College of Nursing, Australia).* 2011;18(1):19-26.

121. McDonald KM, Bryce CL, Graber ML. The patient is in: patient involvement strategies for diagnostic error mitigation. *BMJ quality & safety.* 2013;22 Suppl 2:ii33-ii39.

122. Kim JY, Jung MG, Kwon MH, Noh SO, Kim HJ. Patient involvement in reducing errors during X-ray imaging in an orthopedic outpatient clinic. *Journal of Evaluation in Clinical Practice.* 2017;23(6):1227-1231.

123. Schwappach DL, Frank O, Buschmann U, Babst R. Effects of an educational patient safety campaign on patients' safety behaviours and adverse events. *Journal of Evaluation in Clinical Practice.* 2013;19(2):285-291.

124. Schwappach DLB, Frank O, Koppenberg J, Muller B, Wasserfallen J-B. Patients' and healthcare workers' perceptions of a patient safety advisory. *International journal for quality in health care : journal of the International Society for Quality in Health Care.* 2011;23(6):713-720.

125. Weingart SN, Morway L, Brouillard D, et al. Rating recommendations for consumers about patient safety: sense, common sense, or nonsense? *The Joint Commission Journal on Quality and Patient Safety.* 2009;35(4):206-AP202.

126. AHC Media. Making patients part of the safety effort:  Tip sheet helps reduce medical errors. *Patient Education Management.* 2003;10(8):85-87.

127. Dendere R, Slade C, Burton-Jones A, Sullivan C, Staib A, Janda M. Patient Portals Facilitating Engagement With Inpatient Electronic Medical Records: A Systematic Review. *Journal of medical Internet research.* 2019;21(4):e12779.

128. Duckworth M, Leung E, Fuller T, et al. Nurse, Patient, and Care Partner Perceptions of a Personalized Safety Plan Screensaver. *Journal of Gerontological Nursing.* 2017;43(4):15-22.

129. Oermann MH, Hamilton J, Shook ML. Using the Web to improve seniors' awareness of their role in preventing medical errors. *Journal of nursing care quality.* 2003;18(2):122-128.

130. Gardner J, Hampton MD. The effectiveness of rapid response teams activated by patients or family members of patients admitted to inpatient hospital units: a systematic review protocol. *JBI Database of Systematic Reviews and Implementation Reports.* 2014;12(9):58-68.

131. Guinane J, Hutchinson AM, Bucknall TK. Patient perceptions of deterioration and patient and family activated escalation systems—A qualitative study. *Journal of Clinical Nursing (John Wiley & Sons, Inc).* 2018;27(7-8):1621-1631.

132. Trier H, Valderas JM, Wensing M, Martin HM, Egebart J. Involving patients in patient safety programmes: A scoping review and consensus procedure by the LINNEAUS collaboration on patient safety in primary care. *The European journal of general practice.* 2015;21 Suppl:56-61.

133. Martin HM, Larsen J. Patient involvement in Patient Safety: A literature review about European primary care. *The Danish Institute for Health Services Research for the Danish Society for Patient Safety and the LINNEAUS EURO-PC project.* 2012.

134. Roter DL, Wolff J, Wu A, Hannawa AF. Patient and family empowerment as agents of ambulatory care safety and quality. *BMJ Quality & Safety.* 2017;26(6):508-512.

135. Kingston-Riechers J, Ospina M, Jonsson E, Childs P, McLeod L, Maxted J. Patient safety in primary care. *Edmonton AB: Canadian Patient Safety Institute and BC Patient Safety and Quality Council.* 2010.

136. Lipke B, Gilbert G, Shimer H, et al. Newborn Safety Bundle to Prevent Falls and Promote Safe Sleep. *MCN The American journal of maternal child nursing.* 2018;43(1):32-37.
